# Role of *TIR1/AFB* family genes during grafting in *Carya cathayensis*

**DOI:** 10.3389/fpls.2024.1494579

**Published:** 2024-11-22

**Authors:** Jiaqi Mei, Xiaoyu Tang, Yujie Gu, Huijie Lu, Ying Yang, Qinyuan Shen, Lingwei Yang, Bei Li, Jianfang Zuo, Vijay Pratap Singh, Anket Sharma, Huwei Yuan, Bingsong Zheng

**Affiliations:** ^1^ State Key Laboratory of Subtropical Silviculture, Zhejiang A&F University, Hangzhou, China; ^2^ Plant Physiology Laboratory, Department of Botany, Chaudhary Mahadeo Prasad (C.M.P.) Degree College, University of Allahabad, Prayagraj, India

**Keywords:** auxin, *TIR1/AFB*, Chinese hickory, grafting, Aux/IAA

## Abstract

Auxins play significant roles in plant growth and development. The transporter inhibitor response1/auxin signaling F-box (*TIR1/AFB*) gene family encodes the auxin receptor proteins and plays an essential role in the auxin signaling pathway. Here we identified and characterized the *TIR1/AFB* family in *Carya cathayensis* (Cc) plants (named as *CcTIR1/AFB*). Seven *CcTIR1/AFBs* were identified and further confirmed by cloning. All proteins encoded by these genes conservatively contained two domains, the F-box and leucine-rich repeat (LRR) domains. The *CcTIR1/AFBs* were located in the nucleus. Phylogenetic analysis suggested that CcTIR1/AFBs were evenly scattered in four different subgroups. The cis-acting element analysis indicates that *CcTIR1/AFBs* might be activated by auxin. The spatial and temporal expression of *CcTIR1/AFBs* during grafting suggested that both *CcAFB1* and *CcAFB2* in scions and *CcAFB4* in the rootstocks were significantly upregulated at 3 days after grafting, which indicated the specialization of three *CcAFBs* during grafting. The Y2H assay indicated that three CcAFBs were capable of interacting with CcIAA16, CcIAA27b, and CcIAA29a, among which CcAFB4 interacted strongly with CcIAA1 and CcIAA16. Our study provides the opportunity to understand the potential role of not only *CcTIR1/AFBs* but also special *CcAFBs* (*CcAFB1*, *CcAFB2*, and *CcAFB4*), which is a great aspect to further explore the molecular mechanism during the grafting process.

## 1 Introduction

The auxins play a significant role in the processes of plant development and growth, including embryogenesis, vascular formation, and flower development (Zhao, 2010). The core components of auxin signaling pathway come from three protein families: the commonly acknowledged auxin receptor protein, TIR1/AFB (transporter inhibitor response1/auxin signaling F-box); the transcriptional repressor, Aux/IAA (auxin/indol-3-acetic acid); and the transcription factor, ARF (auxin response factor) (Lavy and Estelle, 2016). TIR1/AFB is one of the key elements of SCF^TIR1/AFB^ complex which serves as the ubiquitin–protein ligase catalyzing the transfer of ubiquitin from E2 (ubiquitin-conjugating enzyme) to the target protein (Dharmasiri et al., 2005; Parry et al., 2009). Aux/IAAs play the role of a transcriptional repressor via forming the heterodimers with ARFs and preventing the interaction between ARFs and DNA sequences (Luo et al., 2018). The ARFs, as the transcription factor, are able to form the homodimer which bind the specific cis-element AuxRE (auxin response element) and activate the expression of genes downstream from the AuxRE (Chandler, 2016). The IAA with high concentration in the nucleus contributes to the formation of TIR/AFB–IAA–Aux/IAA complex and triggers the Aux/IAA degradation, which results in the release of ARFs from ARF–Aux/IAA heterodimer and the activation of gene expression regulated by ARFs. On the contrary, the function of ARFs is repressed at a low concentration of IAA because of the failed formation of the Aux/IAA degradation complex (Guo et al., 2021). Jogawat et al. (2021) have done an in-depth analysis of auxin crosstalk with other hormones along with *TIR1* gene function in plants.

There are six members of the TIR1/AFB protein family which are involved in a variety of development processes and display extensive biological functions in *Arabidopisis*. TIR1 and AFB2, with the ability to interact much stronger with Aux/IAA proteins than AFB1 and AFB3 *in vitro*, are the principal auxin receptors in roots (Parry et al., 2009). AFB3 is involved in auxin signaling modulated by nitrate and participates in the alteration of the root system architecture (Vidal et al., 2010). AFB4 has a significant role in seedlings in response to the elevated temperature, and AFB5 is the principal target of picloram family (Greenham et al., 2011; Prigge et al., 2016). The TIR1/AFB protein contains two critical conserved domains, the F-box domain and the transporter inhibitor response 1 domain (also called leucine-rich repeat (LRR) domain) (Hayashi et al., 2008). The former is capable of interacting with the SPK subunit in the SCF complex, and the latter displayed as a pocket to bind auxin and to interact with Aux/IAA (Kepinski, 2007). However, the reports related to the function of TIR1/AFB protein in the other species are limited.

Plant grafting, an ancient vegetative propagation technique, which connects two cut parts (known as scion and rootstock) originated from either the same or different plant species, is widely used in agriculture (Wang et al., 2017). For plants, grafting contributes to many optimized attributes, like the improvement of tolerance against biotic and abiotic stresses, augmentation of yield, and elongation of the harvest phase (Lee et al., 2010). In *Arabidopsis*, a successful graft union formation starts with necrotic layer development, followed by callus tissue formation and new vascular tissue differentiation within the scion and finished with vascular reconnection between scion and rootstock (Flaishman et al., 2008). Plant hormones such as cytokinin, ethylene, jasmonic acid, and auxin are critical regulators in response to wounding and grafting (Asahina et al., 2011; Iwase et al., 2011). Particularly, auxin plays an important role in vascular development via the TIR1/AFB signaling pathway (Mazur et al., 2020). In *Arabidopsis*, *iaa18* mutant and *tir1afb2afb3* triple mutant are severely incapable of reconnecting the phloem after grafting (Melnyk et al., 2015). In rice, *Ostir1Osafb2* double mutant is no longer capable of generating calli even when being treated with 10 µM of 2,4-dichlorophenoxyacetic acid (Guo et al., 2021). Besides this, *PtrFBL1* and *PtrFBL7*, the homologs of *TIR1/AFBs* in *Populus*, are mainly expressed in cambial and vascular tissues (Shu et al., 2015).

Chinese hickory (*Carya cathayensis* Sarg.) is a well-known economic tree and popular in China for its extremely nutritious nuts, containing high amounts of polyunsaturated fatty acids which aid in the brain development (Huang et al., 2022). However, the long juvenile phase of Chinese hickory seriously represses the enlargement of its industry and the increment of fruit growers’ wealth. Grafting, as an asexual reproduction technology in plant, is capable of shortening the juvenile phase and facilitates the development of the hickory industry efficiently. It is the vascular reconnection between the scion and the rootstock that plays a critical role in avoiding the failure of grafting as reported in previous research (Loupit et al., 2023; Melnyk et al., 2015). A majority of evidence indicates that auxin plays a central role in the progress of plant vascular formation (Sharma and Zheng, 2019). However, knowledge about the involvement of auxin in grafting is still limited. In the current investigation, the *CcTIR1/AFB* gene family, as the auxin receptor, was identified and characterized. Additionally, the potential function of this family during the grafting process in Chinese hickory was further explored in detail. Results of the current investigation may be helpful in further understanding the molecular mechanisms regulated by the *CcTIR1/AFB* gene family during the grafting process.

## 2 Materials and methods

### 2.1 Plant materials

Regarding the grafting union, the rootstock is derived from 2-year-old Chinese hickory trees cultivated for 5 months before grafting in the greenhouse of Zhejiang Agriculture and Forestry University (temperature 25 ± 1°C, 12 h day/12 h night photoperiod, 60%–70% relative humidity, watered once a week), and the scions were collected from the stem of 1-year-old Chinese hickory trees (Yuan et al., 2018).

The conjunction parts of the grafted individuals were sampled at 0, 3, 7, and 14 days (Yang et al., 2022) after grafting, and the sampling time points were determined based on previous research about the changing of morphology during the process of the grafted Chinese hickory’s being successfully reconnected between scions and rootstocks and being alive. The 0-day-after-grafting unions which were sampled just after grafting were taken as the control.

### 2.2 Isolation and cloning of *TIR1/AFB* gene family

The seven *TIR1/AFB* genes were screened and identified from published Chinese hickory genome (Huang et al., 2019). The total RNA as the template for the reverse transcriptase in the subsequence experiments was extracted from the tissue of hickory using TaKaRa MiniBEST Universal RNA Extraction Kit (Takara Bio Inc., Kusatsu, Japan). The cDNA synthesis reaction was performed with the PrimeScript™ II 1^st^ Strand cDNA Synthesis Kit and conducted according to its manufacturer’s protocol. For the first step of that reaction, 1 μg total RNA extracted previously was mixed firstly with 1 μL dNTP mixture (10 Mm each), 1 μL Oligo dT Primer (50 Mm), and the RNase Free dH_2_O up to the volume of 10 μL. Then, the mixture was kept at the condition of 65°C for 5 min and cooled by burying in ice. For the second step, the 10-μL mixture from step one was infused with 4 μL 5×PrimeScript II Buffer, 0.5 μL RNase Inhibitor (40 U/μL), 1 μL PrimeScript II Rtase (200 U/μL), and RNase Free dH_2_O up to the volume of 20 μL in the PCR tube. The PCR tube was put into the thermal cycler and operated with the program as follows: heating at 42°C for 60 min and 70°C for 15 min. The *TIR1/AFB* genes were cloned as per the next several procedures. cDNA was taken as the template for the amplification of target genes with PrimeSTAR Max Premix (2 ×) (Takara Bio Inc., Kusatsu, Japan). The PCR product was purified with FastPure Gel DNA Extraction Mini Kit (Vazyme, Nanjing, China). The purified products were ligated to the cloning vector, pEASY-Blunt Zero, from pEASY-Blunt Zero Cloning Kit (TransGen Biotech, Beijing, China) and transformed into DH5α competent cells of *Escherichia coli* (TransGen Biotech, Beijing, China). The clones grown on selected medium were picked and sent to Sangon Biotech (Shanghai) Co., Ltd. (China) for sequencing.

### 2.3 Sequence analysis of *TIR1/AFB* family genes derived from Chinese hickory

The TIR1/AFB family protein sequences of three other species (*Oryza sativa*, *Populus*, and *Arabidopsis*) were downloaded from Phytozome V13 database (https://phytozome-next.jgi.doe.gov/). Coupled with the seven CcTIR1/AFB protein sequences, multiple sequence alignment was performed with ClustalW, and then the phylogenetic tree of the *TIR1/AFB* family of the four species was constructed based on the neighbor-joining method associated with a bootstrap of 1,000 replicates on the MEGA X software.

The conserved motif distribution was predicted with protein sequences using the MEME Suite set with eight motifs. The exon–intron structure information was acquired from the Chinese hickory genome. Both the motif distribution and the gene structure were visualized with Tbtools (Chen et al., 2020). The protein sequences of TIR1/AFBs were taken to conduct the conserved domain prediction with CDD (conserved domain database) in NCBI and then visualized with R package, ggplot2.

### 2.4 cis-acting element analysis of CcTIR1/AFB promoters

The upstream 2,000-bp sequence of *CcTIR1/AFB* genes, as the promoter of each gene, was acquired from *Carya cathayensis* genome. cis-acting regulatory element analysis was performed for element prediction after submitting the promoter sequences to the Plant-CARE website (http://bioinformatics.psb.ugent.be/webtools/plantcare/html/). The elements related with light, stress, and phytohormone were selected for further analysis. The results were visualized with R package, ggplot2.

### 2.5 Subcellular localization

The *TIR/AFB* family genes ligated to pEASY-Blunt Zero vector were amplified by PCR and purified. The pCambia1300-GFP vector was cut and linearized with two restriction endonucleases, KpnI and XbaI (Takara Bio Inc., Kusatsu, Japan). The amplified fragments were inserted into the pCambia1300-GFP vector by homologous recombination with ClonExpress II One Step Cloning Kit (Vazyme, Nanjing, China). The constructed vector for subcellular localization was transformed into DH5α competent cells of *Escherichia coli* (TransGen Biotech, Beijing, China). The clones were picked and sent to Sangon Biotech (Shanghai) Co., Ltd. (China) for sequencing. The recombinant plasmids were extracted from DH5α and transformed into GV3101 chemically competent cells of *Agrobacterium tumefaciens* (Weidi Bio., Shanghai, China). The tobaccos were planted and cultivated for some 6 weeks based on previous research (Yang et al., 2021). The target genes having been integrated into GV3101 are transiently co-transformed into the tobacco leaves associated with nuclear-localized marker. After cultivation in the dark for 48 h, the tobacco leaves were cut and observed to detect fluorescent signals using a confocal microscope with argon lasers at the wavelength of 488 and 594 nm.

### 2.6 RNA extraction and quantitative RT-PCR

In order to uncover the function of *TIR1/AFB* in the process of growing grafted Chinese hickory, qRT-PCR was performed to study the target gene expression in that process. The scions and the rootstocks of the graft union were separated gently. The conjunction parts of both the scions and rootstocks at each time point (0, 3, 7, and 14 days after grafting) were sampled separately for RNA extraction. In addition, the RNA originated from the scions and rootstocks were extracted with TaKaRa MiniBEST Universal RNA Extraction Kit (Takara Bio Inc., Kusatsu, Japan) and based on its manufacturer’s protocol. Total RNA was extracted. The 0 day after grafting was taken as the control. cDNA synthesis for qRT-PCR was performed using PrimeScript™ RT Master Mix (Perfect Real Time) (Takara Bio Inc., Kusatsu, Japan).

The primers designed for qRT-PCR are designed and listed in **Supplementary Table S1**. cDNA was diluted into 50 ng/μL and used as the template for qRT-PCR amplification. *CcActin*, the *Actin* gene in Chinese hickory, was taken as the reference gene functioning in calculating the relative fold change of *CcTIR1/AFBs* from both the different parts of the graft union and the different time points after grafting. The amplification system was set according to TB Green^®^ Premix Ex Taq™ (Tli RnaseH Plus) (Takara Bio Inc., Kusatsu, Japan). The qRT-PCR procedures were set as follows: 95°C for 3 min, 40 cycles of 95°C for 10 s, and 60°C for 30 s. All of the expression analyses were repeated four times. The relative gene expression of *CcTIR1/AFBs* was calculated based on the 2^−ΔΔCt^ method.

### 2.7 Protein interaction prediction and Y2H assay

The coding sequences of *CcTIR1/AFBs* and *CcIAAs* were acquired from NCBI. The coding sequences were translated into protein sequences and then submitted to the STRING database website (https://cn.string-db.org/). The homologs of those proteins in *Arabidopsis* were matched and selected based on the identity score to construct the predicted protein interaction network.

Y2H assay was performed to test the interaction between CcTIR1/AFBs and CcIAAs. The primer was designed based on the genome data of Chinese hickory. The coding sequences of both *CcTIR1/AFBs* and *CcIAAs* were amplified from the cDNA of Chinese hickory and were inserted into the bait vector pGADT7 and the prey vector pGBKT7, respectively. The two vectors were linearized with EcoRI and BamHI during plasmid construction. The bait (*CcTIR1/AFB*) and prey (*CcIAA*) constructs were co-transformed into the yeast strain Y2Hgold (Weidi Bio., Shanghai, China) using the lithium acetate method according to the manufacturer’s instructions (Clontech, Mountain View, CA, USA). The transformants were selected on the (DDO) SD-Trp/-Leu medium (Coolaber, Beijing, China) and were further confirmed by being spotted on the DDO medium for at least 2 days at 30°C. To examine the interactions between CcTIR1/AFBs and CcIAAs and to uncover the role of IAA in the interaction, the transformants were transferred from SD-Trp/-Leu medium to SD-Trp/-Leu/-Ade/-His (Coolaber, Beijing, China) using a series of concentrations of IAA ranging from 0 to 100 mM and were cultured at 30°C (Prigge et al., 2010).

### 2.8 Statistical analysis

One-way analysis of variance was performed, and multiple comparison was conducted with the Duncan method at the significant level of 0.05 by the R package, agricolae.

## 3 Results

### 3.1 Identification and cloning of *TIR1/AFB* family genes from Chinese hickory

**Figure 1:**
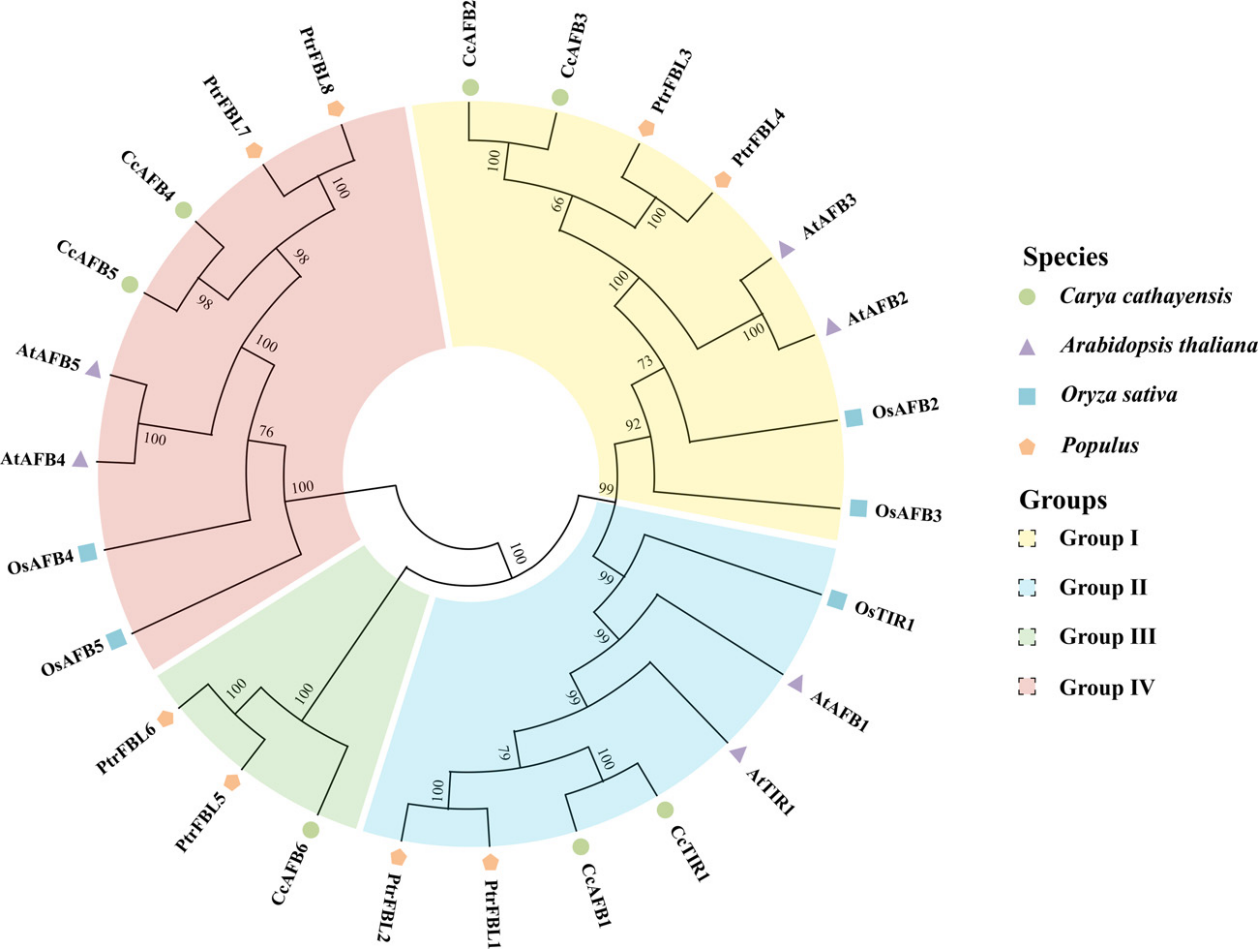
Phylogenetic tree of *TIR1/AFB* derived from *Carya cathayensis*, *Oryza sativa*, *Populus*, and *Arabidopsis*. The phylogenetic tree was created by MEGA X using neighbor-joining method with 1,000 bootstrap replicates. The numbers located in each node indicate bootstrap values. The *TIR1/AFB* family members were divided into four groups. Different species and groups were distinguished with the colored dots or polygons and the colored blocks, respectively. TIR1/AFB, transporter inhibitor response1/auxin signaling F-box. CcTIR1/AFB, AtTIR1/AFB, OsTIR1/AFB, and PtrFBL6 represent the TIR1/AFB family in *Carya cathayensis*, *Arabidopsis*, *Oryza sativa*, and *Populus*, respectively.

Seven *TIR/AFB* sequences were identified totally based on the Chinese genome data. To confirm the accuracy of the sequences, seven pairs of primers used for sequence cloning were designed according to the genome data. The sequencing and alignment results indicated that the sequences cloned from the cDNA of Chinese hickory are identical with those searched from the Chinese hickory genome. The seven *CcTIR1/AFB* genes are named compared to their putative orthologs in *Arabidopsis* based on the phylogenetic relationships (**Figure 1**). The basic information about the *CcTIR1/AFB* sequences, including gene name, gene id, CDS length, and properties of the predicted peptides, is listed in **Table 1**. The CDS lengths of *CcTIR1/AFB* range from 1,716 base pairs (*CcAFB3*) to 1,896 base pairs (*CcAFB4*); the peptide lengths range from 571 amino acids (CcAFB3) to 631 amino acids (CcAFB4) and the molecular weights range from 64.19 to 70.61 kDa. The predicted isoelectric points of CcTIR1/AFBs have a range from 5.42 (CcAFB4) to 6.67 (CcAFB3). As predicted, all the family members of CcTIR1/AFB are localized in the nucleus.

**Table 1:** Sequence information about *Carya cathayensis* transporter inhibitor response1/auxin signaling F-box (*CcTIR1/AFB*) gene family.

Gene name	Gene ID	CDS length (bp)	Peptide length (aa)	MW (Da)	pI	Predicted localization
*CcTIR1*	CCA0981S0065	1,755	584	65,621.97	6.28	Nucleus
*CcAFB1*	CCA0535S0053	1,755	584	65,394.77	6.01	Nucleus
*CcAFB2*	CCA1250S0070	1,722	573	64,610.43	6.28	Nucleus
*CcAFB3*	CCA1539S0035	1,716	571	64,194.08	6.67	Nucleus
*CcAFB4*	CCA0533S0235	1,896	631	70,606.37	5.42	Nucleus
*CcAFB5*	CCA1097S0042	1,878	625	69,196.82	5.85	Nucleus
*CcAFB6*	CCA0729S0024	1,740	579	65,165.38	6.12	Nucleus

CDS, coding sequence; MW, molecular weight; pI, isoelectric point.

**Figure 2:**
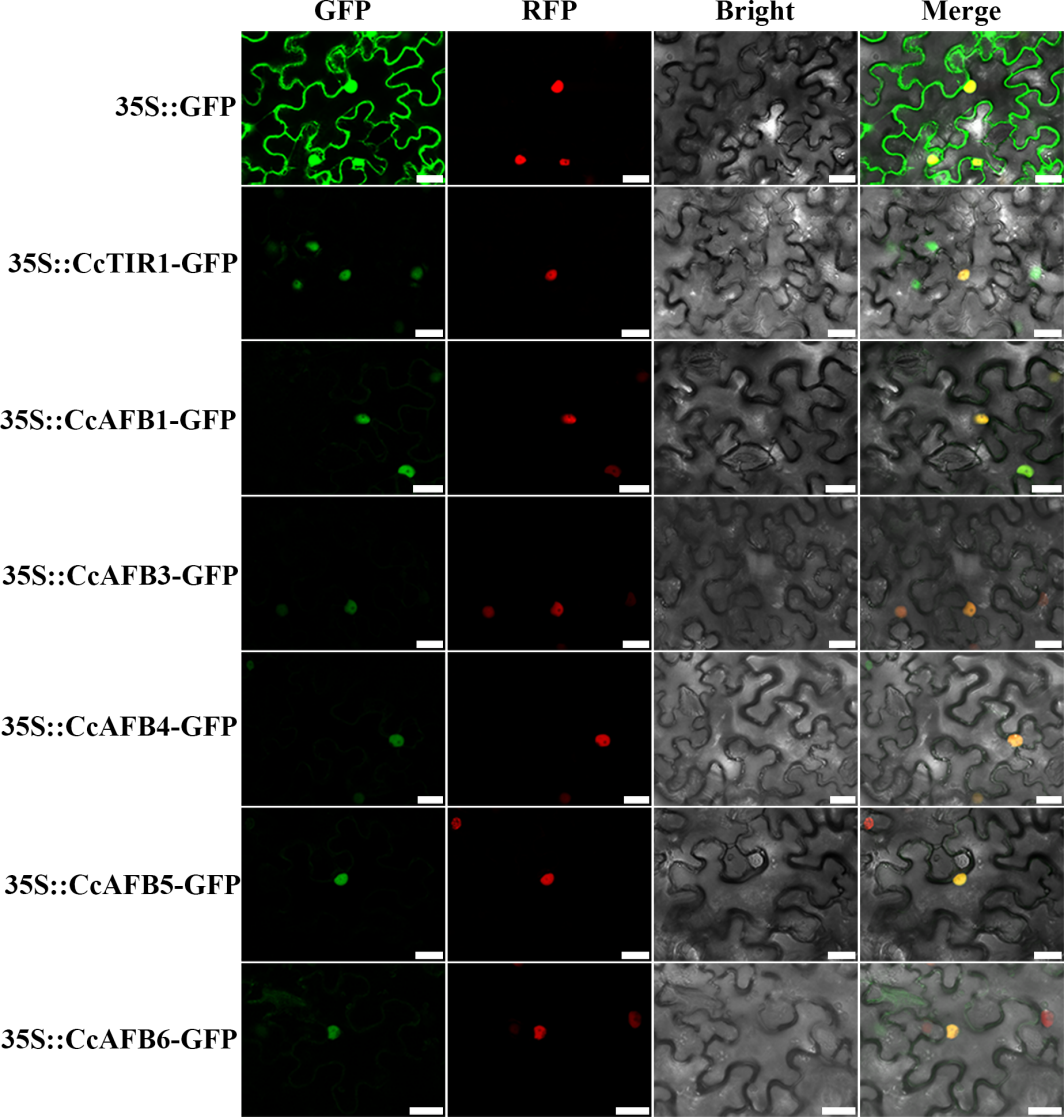
Subcellular localization of CcTIR1/AFB in *Nicotiana tabacum* leaves. The CDS sequences of *CcTIR1/AFBs* are inserted into the pCambia 1300-GFP vector and are under the control of 35S promoter. GFP, GFP fluorescence; RFP, RFP fluorescence; Bright, bright field; Merge, GFP/RFP/bright field overlay. The bar indicates 25 μm. CcTIR1/AFB, *Carya cathayensis* transporter inhibitor response1/auxin signaling F-box; GFP, green florescent protein; RFP, red florescent protein; 35S, CaMV35S promoter.

To explore the localization on which the CcTIR1/AFBs might perform their functions, some of the *CcTIR1/AFBs* were selected randomly and transiently transformed into tobacco leaves. The results showed that the CcTIR1/AFB proteins were located in the cellular nucleus (**Figure 2**).

### 3.2 Phylogenetic relationship of TIR1/AFB proteins derived from *Carya cathayensis*, *Oryza sativa*, *Populus*, and *Arabidopsis*

In order to uncover the phylogenetic organization of *CcTIR1/AFB* and to reveal the phylogenetic relationship of *TIR1/AFB* of *Carya cathayensis* and of other different species, the *TIR1/AFB* family members from three species, *Oryza sativa*, *Populus*, and *Arabidopsis*, were chosen for the phylogenetic tree construction using neighbor-joining method with MEGA X (**Figure 1**). According to the phylogenetic relationships, we divided the *TIR1/AFB* family members into four groups and successively named those as clades I, II, III, and IV. The members of each class were varied from three to eight. The *TIR1/AFBs* from *Carya cathayensis* were scattered in all four groups and always clustered stronger with those from *Populus*. *CcTIR1/AFB* was distributed almost evenly in each class. The number of *CcTIR1/AFB* in clades I, II, III, and IV were two, two, one, and two, respectively. It was noteworthy that only group III contained the family genes from *Carya cathayensis* and *Populus*, but the other groups contained those family genes from all four species.

### 3.3 Gene structure, motif, and domain analysis of CcTIR1/AFB proteins in Chinese hickory

**Figure 3:**
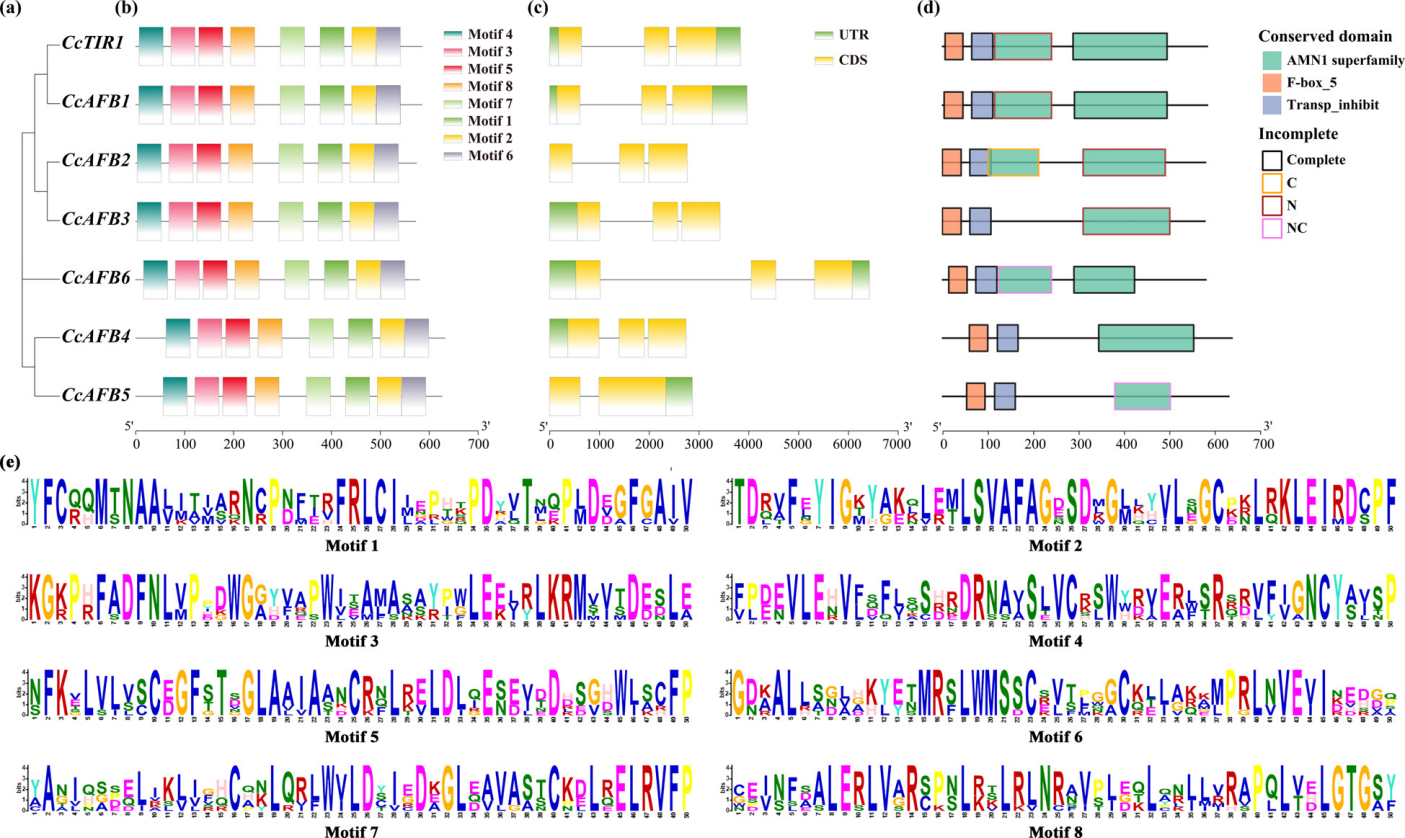
Gene structure, conserved motifs, and conserved domains of CcTIR1/AFB. **(A)** Phylogenetic tree of CcTIR1/AFBs being constructed with the protein sequences. **(B)** Motif distributions of the CcTIR1/AFB proteins generated in MEME Suite. Eight motifs were represented with eight blocks with different colors. **(C)** Exon–intron structure of CcTIR1/AFBs. The UTRs (untranslated region), exons, and introns were represented with green blocks, yellow blocks, and gray lines, respectively. **(D)** Conserved domain of CcTIR1/AFBs. The red, blue, and green blocks indicate the TIR1 protein domain, the F-box domain, and the AMN1 superfamily domain, respectively. The outline of the blocks represented the incomplete degree of the AMN1 superfamily domains. The description of the legends is as follows: Complete, the domain was complete; C, the domain was incomplete in C terminal; N, incomplete in N terminal; NC, incomplete in both N and C terminals. **(E)** Information of the discovered motifs from CcTIR1/AFBs. The size of the letters suggests the conservation degree of the amino acids. CcTIR1/AFB, *Carya cathayensis* transporter inhibitor response1/auxin signaling F-box; UTR, untranslated region; CDS, coding sequence; AMN1, antagonist of mitotic exit network protein 1; Trans_inhibit, transporter inhibitor response 1 domain.

The investigation of the gene structure, the conserved domains, and the motifs of CcTIR1/AFB showed that the protein sequences of CcTIR1/AFBs were similar and conserved. The construction of phylogenetic tree of CcTIR1/AFBs was performed with MEGA X based on neighbor-joining method (**Figure 3A**). Their exon–intron structure was obtained from the Chinese hickory genome. Motif analysis was conducted using the MEME Suite with eight conserved motifs. All eight motifs were detected in every CcTIR1/AFB protein (**Figure 3B**). Except for CcAFB4 which contained two exons and one intron, all of the other six CcTIR1/AFBs contained three exons and two introns (**Figure 3C**). Conserved domain analysis was performed using CCD and visualized with ggplot2. The results showed that all seven CcTIR1/AFBs contained both the F-box domain and the LRR domain (also called transporter inhibitor response 1 domain) (Hayashi et al., 2008; Sheard et al., 2010). Besides that, all the CcTIR1/AFBs contained one to two complete or incomplete AMN1 (antagonist of mitotic exit network protein 1) superfamily domain which was composed of a number of leucine-rich repeats (**Figures 3D, E**) (Wang et al., 2003).

### 3.4 cis-acting element analysis of CcTIR1/AFB promoters

It is extremely vital to explore the *CcTIR1/AFBs* transcriptional regulation and to infer their biological function. Therefore, prediction of the cis-acting elements was conducted by plant-CARE after submitting the upstream 2,000 base pairs of *CcTIR1/AFBs*. All CcTIR1/AFB promoters contained six kinds of cis-acting element of interest, including light response element, temperature response element, stress response element, and three kinds of phytohormone response elements, including auxin response elements, abscisic acid response elements, and MeJA response elements. Each element class contained 12, one, one, two, one, and two kinds of cis-acting elements, respectively.

**Figure 4:**
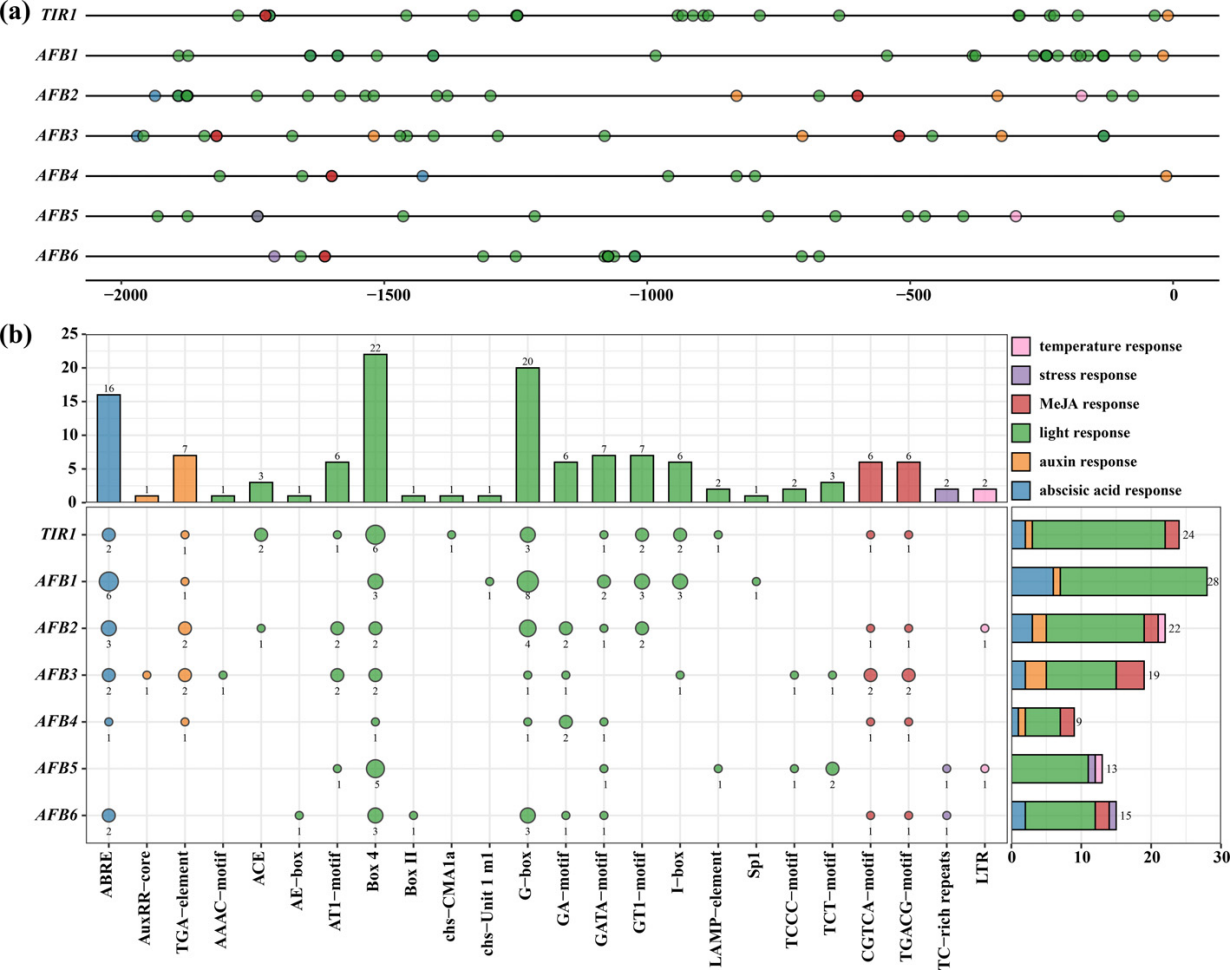
cis-acting element analysis of *CcTIR1/AFB* promoters. The upstream 2,000-bp sequences of *CcTIR1/AFB* genes were identified as the promoters. **(A)** Distribution of cis-acting elements on the promoter of *CcTIR1/AFB*. **(B)** The amounts of cis-acting elements belonging to different response classes on the promoter of each gene. The color of each bubble indicates each responsiveness class. The size of the bubble on the left plot suggests the quantity of each cis-acting element type, and the exact numbers are shown below the bubbles. The bar plot on the right illustrates the relative amounts of each response element on the promoter of each gene. *TIR1/AFB*, *Carya cathayensis* transporter inhibitor response1/auxin signaling F-box. On the x-axis, ABRE to LTR represent different *cis*-acting elements.

The light response elements, as the majority of the cis-acting types of *CcTIR1/AFB* promoters, are scattered evenly on the promoters, suggesting that *CcTIR1/AFB* might play a significant role in the response to light. The stress and temperature response elements were set upstream and downstream of the promoter, respectively. As for the phytohormone response element, the auxin response elements mostly occupy the downstream part of the promoters, but both the abscisic acid response and the MeJA response elements are mostly concentrated on the upstream part (**Figure 4A**).

The promoter of *CcTIR1/AFBs*, from *CcTIR1* to *CcAFB6*, contained 24, 28, 22, 19, nine, 12, and 15 cis-acting elements of interesting, respectively. The *CcTIR1* promoter contains four classes of cis-acting elements of interest, except for the temperature response and the stress response. *CcAFB1* contains the most abscisic acid response elements (ABRE) and light response elements. *CcAFB2* possesses five classes of response types of cis-acting elements except for the stress response. According to the prediction, the temperature and stress response elements only exist in the promoter of *CcAFB2*, *CcAFB5*, and *CcAFB6* and occupy a small part of all *CcTIR1/AFB* cis-acting elements. As for the cis-acting elements of the promoters, Box-4 and G-box are the two most common cis-acting elements belonging to the light response, the number of which are 22 and 20, respectively. ABRE (abscisic acid response elements) follows with a total number of 16. The element classes with the minimum numbers are the temperature response class and the stress response class, which only contained two LTR and two TC-rich repeats, respectively (**Figure 4B**).

### 3.5 The interactions between CcTIR1/AFBs and CcIAAs are regulated by different concentrations of IAA

The *TIR1/AFB* genes play a critical role in the auxin signaling pathway. The interactions of TIR1/AFB proteins with Aux/IAA proteins contribute to the degradation of Aux/IAA, resulting in the release of ARFs which, with the presence of IAA, activate the expression genes involved in a series of biological processes. Based on that, it is necessary to explore the interactions between CcTIR1/AFBs and Cc/IAAs.

**Figure 5:**
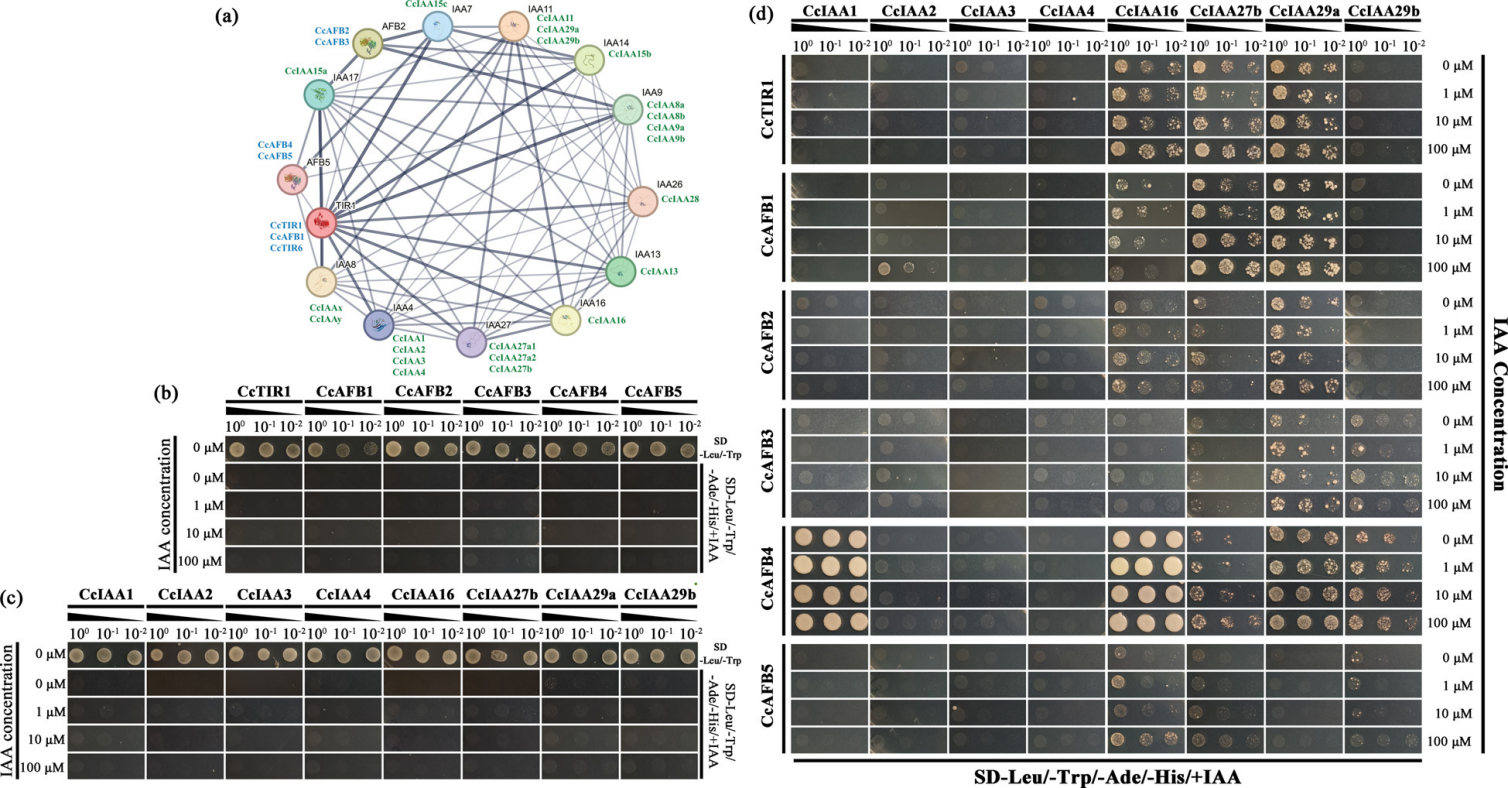
Interactions between CcTIR/AFBs and CcIAAs. **(A)** Prediction of the interaction network of CcTIR/AFBs and CcIAAs. Both the TIR1/AFBs and IAAs from *Arabidopsis thaliana* are shown with black names. CcTIR1/AFBs and CcIAAs are shown with blue and green names, respectively, and were labeled near to the counterpart in *Arabidopsis* based on the sequence similarity. The boldness of the edge lines in the network suggests the confidence of the interactions. **(B)** Identification of the co-transformed yeast (CcTIR1/AFBs in pGADT7 and pGBKT7 empty vector) with DDO (SD-Leu/-Trp) medium and the self-activation test with the QDO associated with different IAA concentrations (SD-Leu/-Trp/-Ade-/His/+IAA). **(C)** Identification of the co-transformed yeast (CcIAAs in pGBKT7 and pGADT7 empty vector) with DDO medium and the self-activation test with the QDO associated with different IAA concentrations. **(D)** Yeast two-hybrid experiment used to identify the 6 CcTIR1/AFBs’ interactions with eight CcIAAs. The CcTIR1/AFBs and CcIAAs were inserted into pGADT7 and pGBKT7, respectively. Diploids containing GAL4-AD-CcTIR1/AFBs and GAL4-BD-CcIAAs were generated by the co-transforming method and were spotted on the QDO selective medium with increasing concentrations of IAA. CcTIR1/AFB, *Carya cathayensis* transporter inhibitor response1/auxin signaling F-box; CcIAA, *Carya cathayensis* auxin/indole-3-acetic acid; IAA, indole acetic acid; SD, synthetic defined medium; Leu, leucine; Trp, tryptophan; Ade, adenine; His, histidine; DDO, double dropout medium; QDO, quadruple dropout medium.

The amino acid sequences of CcTIR1/AFB and of CcIAAs acquired from NCBI were selected to perform the interaction prediction with the online database, STRING. Each Chinese hickory sequence was mapped to the homologs in *Arabidopsis* based on sequence similarity. The *Arabidopsis* homologs were taken to construct the protein interaction network. The most similar Chinese hickory sequences were labeled next to the *Arabidopsis* homologs. The edge boldness indicated the interaction confidence. The predicted interaction network was composed of 14 nodes and 66 edges. There were a variety of strong interactions not only between two gene families but also in the same gene family (**Figure 5A**).

Besides that, six CcTIR1/AFBs (CcTIR1 and CcAFB1–5) and eight CcIAAs (CcIAA1–4, CcIAA16, CcIAA27b, CcIAA29a, and CcIAA29b) were selected randomly to confirm the interactions between CcTIR1/AFBs and Aux/IAA via the Y2H assay. The diploids were generated and confirmed with DDO medium (**Figure 5B**). The diploids transformed only with CcTIR1/AFBs or only with CcIAAs were not able to grow ordinarily on the DDO medium, which suggested that both of them could not activate the reporter genes independently (**Figure 5C**). From CcTIR1/AFBs’ perspective, each of the three CcTIR1/AFBs (CcTIR1, CcAFB1, and CcAFB2) was able to interact with CcIAA16, CcIAA27b, and CcIAA29a. Besides that, CcAFB3 shows the ability to interact with CcIAA27, CcIAA29a, and CcIAA29b. There also existed interactions between CcAFB5 and three CcIAAs (CcIAA16, CcIAA27b, and CcIAA29b). Particularly, CcAFB4 interacted with five CcIAAs (CcIAA1, CcIAA16, CcIAA27b, CcIAA29a and CcIAA29b). The interaction strength between CcAFB4 and both CcIAA1 and CcIAA16 was stronger than the other tested interaction combinations. Meanwhile, from the CcIAAs’ perspective, the results showed that CcIAA2, CcIAA3, and CcIAA4 were incapable of interacting with any of the six TIR1/AFBs. On the contrary, CcIAA16, CcIAA27b, and CcIAA29a could interact with the majority of the six CcTIR1/AFBs. CcIAA29b interacted with half of the selected CcTIR1/AFBs (CcAFB3, CcAFB4, and CcAFB5). CcIAA1 interacted only with CcAFB4, which was associated with strong interaction strength. As the concentrations of IAA were increased, the yeast grew more quickly in the QDO medium, which indicated that the IAA molecule could strengthen the interactions between CcTIR1/AFBs and CcIAAs (**Figure 5D**).

### 3.6 Expression profiles of *CcTIR1/AFB* genes during grafting of Chinese hickory

**Figure 6:**
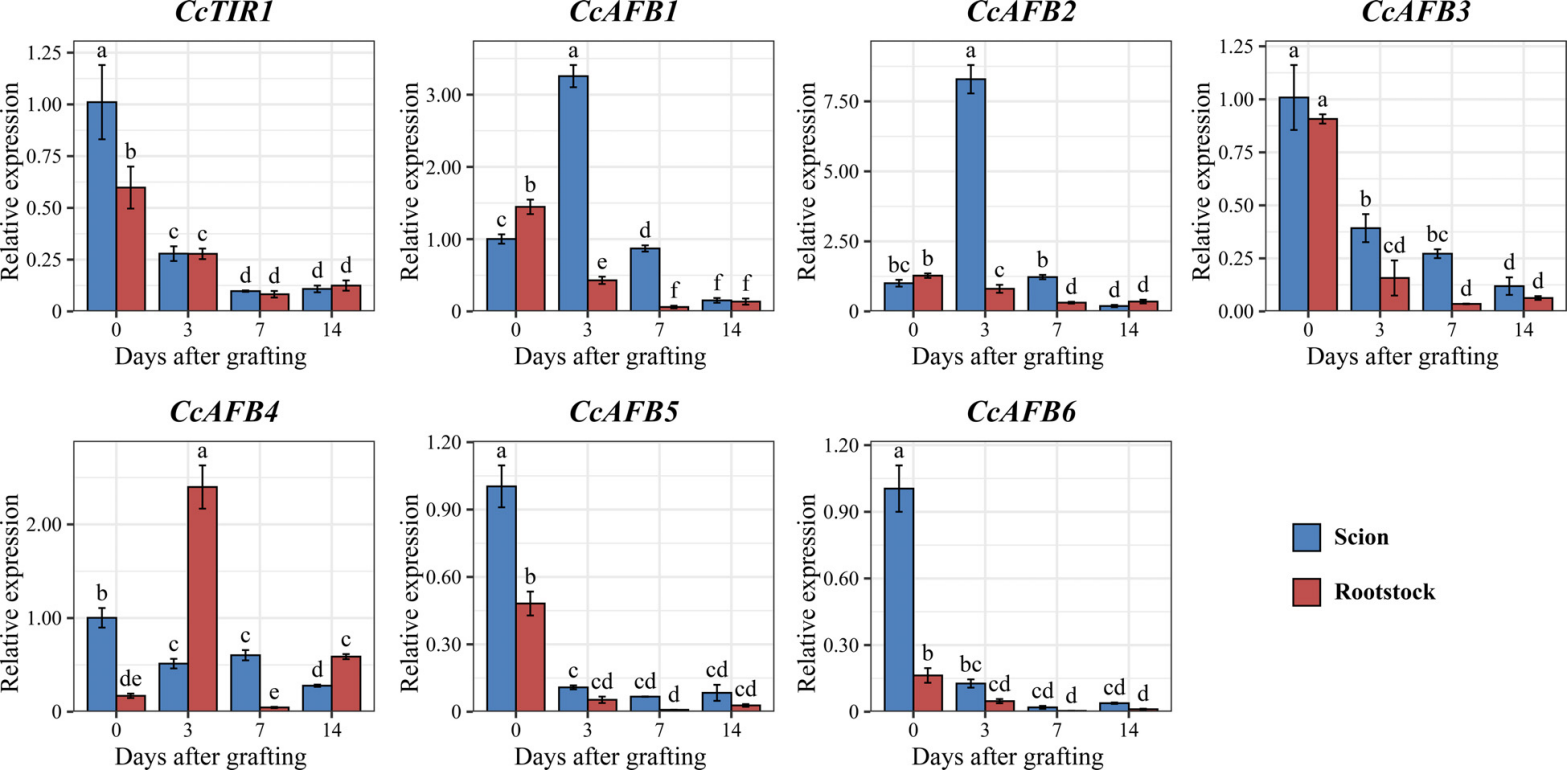
Expression pattern of *CcTIR1/AFB* genes both in scion and in rootstock at different stages (0, 3, 7, and 14 days after grafting). The letters above the error bar display the significant differences between each sample group and are determined by Duncan’s test (*p* < 0.05). CcTIR1/AFB, *Carya cathayensis* transporter inhibitor response1/auxin signaling F-box).

To examine the response of *CcTIR1/AFB* genes during the grafting process of Chinese hickory, the relative expression of *CcTIR1/AFBs*, both at different stages during grafting (0, 3, 7, and 14 days after grafting) and at different parts of the grafting units (the scion and rootstock of Chinese hickory), was quantified using RT-qPCR. It was shown that the expression of most *CcTIR1/AFBs* (*CcTIR1*, *CcAFB3*, *CcAFB5*, and *CcAFB6*), with the highest expression at 0 days after grafting (DAG), was downregulated after grafting both in the scion and the rootstock. Those *CcTIR1/AFBs* were mostly expressed higher in the scion than in the rootstock at the same stage after grafting. Besides that, *CcAFB1* and *CcAFB2* show a similar expression pattern: the expression of *CcAFB1/CcAFB2* is lower in the scion than in the rootstock at 0 DAG. The expression of *CcAFB1/CcAFB2* in the scion was significantly upregulated at 3 DAG and gradually downregulated until 14 DAG and that in rootstock are downregulated from 0 DAG to 14 DAG. The expressions of *CcAFB1/CcAFB2* in both scion and rootstock were decreased to nearly the same low at 14 DAG. Finally, *CcAFB4* displayed a distinct manner compared with the others. The expression of *CcAFB4* was downregulated in the scion but upregulated firstly, downregulated secondly, and upregulated finally in the rootstock. In general, the results suggested that *CcTIR1*, *CcAFB3*, *CcAFB5*, and *CcAFB6* might function similarly and *CcAFB1*, *CcAFB2*, and *CcAFB4* might play a critical role in the Chinese hickory grafting process (**Figure 6**).

## 4 Discussion

Auxins are involved in a series of plant growth and development processes via the auxin signaling pathway (Carrillo et al., 2023). The *TIR1/AFB* family works as the auxin molecule receptor and plays a vital role in that signaling pathway (Ang and Østergaard, 2023). So far, the *TIR1/AFB* families containing all members have been identified in a few plant species, including six members in *Arabidopsis* (Parry et al., 2009), five members in rice (Guo et al., 2021), eight members in *Populus* (Shu et al., 2015), and 18 members in *Brassica juncea* (Cai et al., 2019), which indicates that the *TIR1/AFB* gene family is small in most plant species. The Chinese hickory (*Carya cathayensis*), classified in the Juglandaceae family, is known for its nutritious nuts and economic value (Huang et al., 2022). In our study, seven *CcTIR1/AFBs* were identified based on the genome data and were cloned for further confirmation. The *TIR1/AFB* gene family in Chinese hickory is larger than in *Arabidopsis* and in rice but smaller than in *Populus* and *Brassica juncea*.

The *TIR1/AFB* gene was firstly reported in 1998 and called transport inhibitor response 1 because of the deficiency of auxin transport in the *Arabidopsis* mutant (*tir1*) (Ruegger et al., 1998). Then, *TIR1* and its homologies (*AFBs*) are identified as the auxin receptor (Dharmasiri et al., 2005; Greenham et al., 2011). The TIR1/AFB proteins mainly contained two conserved domains, F-box domain and LRR domain. The former domain is required for TIR1/AFBs to interact with the SKP protein, which facilitates the formation of SCF complex, and the latter containing a number of leucine-rich repeats is required for them to bind auxin and to interact with Aux/IAAs (Yu et al., 2015). In our study, all seven CcTIR1/AFBs contain one F-box domain and one LRR domain on the N terminus of the protein, which is consistent with BjuTIR1/AFBs as described in *Brassica juncea* (Cai et al., 2019).

The ability of TIR1/AFBs to interact with Aux/IAAs, whose strength is enhanced along with the increment of auxin concentration, has been reported in other species with the yeast two-hybrid assay. In *Arabidopsis*, TIR1, AFB1, and AFB2 are able to interact with IAA3, IAA5, IAA7, and IAA8, respectively (Calderón et al., 2012). Besides that, the TIR1 can also interact with IAA1 (Gilkerson et al., 2015) and IAA17 (Nan et al., 2014). In peas, PsTIR1a, PsTIR1b, PsAFB4, and PsAFB6 are able to interact with PsIAA6 (Ozga et al., 2022). In *Brassica napus*, BnTIR1 is capable of interacting with BnIAA7 (Ping et al., 2022). Both the TIR1/AFB family proteins and the Aux/IAA family proteins are mostly located in the cell nucleus and influence the transcription response directly in many other species, such as *Arabidopsis* (Prigge et al., 2020), rice (Guo et al., 2021), and rapeseed (Ping et al., 2022). In our study, it is firstly predicted with Cell-PLoc and further confirmed with the subcellular localization assay that the CcTIR1/AFBs of Chinese hickory are located in the nucleus, which increased the possibilities of spatial interactions between CcTIR1/AFBs and CcIAAs. Given that, the yeast two-hybrid assay associated with different auxin concentrations was conducted. The results show that each of the CcTIR1/AFBs being tested is capable of interacting with some CcIAAs. As the auxin concentration is increased, the interaction of each combination is strengthened. The results also suggest that the CcTIR1/AFBs interact with CcIAAs in an auxin-dependent way in Chinese hickory, which has also been reported in *Arabidopsis* (Yu et al., 2013). CcTIR1, CcAFB1, and CcAFB2 are able to interact with CcIAA16, CcIAA27b, and CcIAA29a, respectively. It is suggested that CcTIR1, CcAFB1, and CcAFB2 might be similar in function based on the Y2H assay. Interestingly, CcAFB4 shows an interaction pattern distinct from the other CcTIR1/AFBs. CcAFB4 shows a strong ability to interact with CcIAA1 and CcIAA16. It is indicated that CcAFB4 might show a different manner in the biological process from the other CcTIR1/AFBs.

Both the scions and the rootstocks are essentially the plant segments cut from different plant individuals (Wang et al., 2017). A successful grafting mostly depends on the adhesion of scions and rootstocks, a process called graft union formation (Gautier et al., 2019). The vascular tissues from both the scion and the rootstock are regenerated and reconnected in that process, which determines the plant survival after being grafted (Melnyk et al., 2018). Auxin and auxin signaling pathway play a critical role in vascular regeneration after grafting—for instance, auxin alters PIN polarity via the Aux/IAA-ARF-dependent pathway to facilitate the regeneration of vascular tissue at the wound (Sauer et al., 2006). *ARF6* and *ARF8* are involved in the regulation of tissue reunion in the *Arabidopsis* stems (Weerasak et al., 2014). The *TIR1/AFB* gene family plays as the auxin receptor and the necessary element in the auxin signaling pathway (Salehin et al., 2015). The TIR1/AFB signaling pathway is essential for vascular regeneration after wounding (Mazur et al., 2020). In order to understand the role of CcTIR1/AFBs during Chinese hickory grafting, their expression patterns in both the scion and the rootstock of Chinese hickory at different time points were analyzed. The four time points were determined according to the morphological changes based on the previous study, such as isolation layer formation at 3 DAG, callus formation at 7 DAG, and vascular reconnection at 14 DAG (Yang et al., 2022). Particularly, both CcAFB1 and CcAFB2 in the scion and CcAFB4 in the rootstock are significantly upregulated at 3 DAG. The special expression pattern indicates the special role of CcAFB1, CcAFB2, and CcAFB4 in Chinese hickory grafting. In *Arabidopsis*, phloem reconnection is severely affected at 4 DAG in the triple mutant, *tir1afb2afb3*, which suggests that *TIR1*, *AFB2*, and *AFB3* are indispensable for vascular regeneration (Melnyk et al., 2015). TIR1/AFBs are capable of interacting with Aux/IAAs and facilitate the degradation of the latter. Aux/IAA might prevent the vascular tissue from generation—for instance, the downregulation of *SlIAA15* contributes to the increasing number of xylem cells in tomato (Deng et al., 2014). In our study, CcIAA16, CcIAA27b, and CcIAA29a are able to interact with CcAFB1, CcAFB2, and CcAFB4. CcIAA1 and CcIAA16 interact with CcAFB4 strongly. The cis-acting element analysis showed that CcAFB1, CcAFB2, and CcAFB4 might be involved in the auxin response process. These results indicate that they are worthy of being studied further in Chinese hickory grafting.

## 5 Conclusion

Chinese hickory is an economic nut tree famous for its nutrient value and is broadly planted in east of China. In our study, seven *CcTIR1/AFB* family genes were identified, cloned, and characterized. The expression pattern analysis and the Y2H assay further indicate the potential role of the *CcTIR1/AFB* family and provide the special *CcAFBs* worthy of further study to explore the molecular mechanism during the grafting process.

## Data Availability

The datasets presented in this study can be found in online repositories. The names of the repository/repositories and accession number(s) can be found in the article/**Supplementary Material**.

## References

[B1] Ang A. C. H. Østergaard L. (2023). Save your TIRs-more to auxin than meets the eye. New Phytol. 238, 971–976. doi: 10.1111/nph.18783 36721296 PMC10952682

[B2] Asahina M. Azuma K. Pitaksaringkarn W. Yamazaki T. Mitsuda N. Ohme-Takagi M.. (2011). Spatially selective hormonal control of RAP2.6L and ANAC071 transcription factors involved in tissue reunion in *Arabidopsis* . Proc. Natl. Acad. Sci. U. S. A. 108, 16128–16132. doi: 10.1073/pnas.1110443108 21911380 PMC3179063

[B3] Cai Z. Zeng D. Liao J. Cheng C. Sahito Z. A. Xiang M.. (2019). Genome-wide analysis of auxin receptor family genes in Brassica juncea var. tumida. Genes 10, 165. doi: 10.3390/genes10020165 30791673 PMC6410323

[B4] Calderón V. I. A. Lee S. Oliveira C. D. Ivetac A. Brandt W. Armitage L.. (2012). A combinatorial TIR1/AFB-Aux/IAA co-receptor system for differential sensing of auxin. Nat. Chem. Biol. 8, 477–485. doi: 10.1038/nchembio.926 22466420 PMC3331960

[B5] Carrillo C. V. P. Hernandez G. J. Mutte S. K. Weijers D. (2023). The birth of a giant: evolutionary insights into the origin of auxin responses in plants. EMBO J. 42, e113018. doi: 10.15252/embj.2022113018 36786017 PMC10015382

[B6] Chandler J. W. (2016). Auxin response factors. Plant Cell Environ. 39, 1014–1028. doi: 10.1111/pce.12662 26487015

[B7] Chen C. Chen H. Zhang Y. Thomas H. R. Frank M. H. He Y. (2020). TBtools: an integrative toolkit developed for interactive analyses of big biological data. Mol. Plant 13, 1194–1202. doi: 10.1016/j.molp.2020.06.009 32585190

[B8] Deng W. Yan F. Liu M. Wang X. Li Z. (2014). Down-regulation of SlIAA15 in tomato altered stem xylem development and production of volatile compounds in leaf exudates. Plant Signal Behav. 7, 911–913. doi: 10.4161/psb.20723 PMC3474683 22836503

[B9] Dharmasiri N. Dharmasiri S. Estelle M. (2005). The F-box protein TIR1 is an auxin receptor. Nature 435, 441–445. doi: 10.1038/nature03543 15917797

[B10] Flaishman M. A. Loginovsky K. Golobowich S. Lev-Yadun S. (2008). *Arabidopsis thaliana* as a model system for graft union development in homografts and heterografts. J. Plant Growth Regul. 27, 231–239. doi: 10.1007/s00344-008-9050-y

[B11] Gautier A. T. Chambaud C. Brocard L. Ollat N. Gambetta G. A. Delrot S.. (2019). Merging genotypes: graft union formation and scion-rootstock interactions. J. Exp. Bot. 70, 747–755. doi: 10.1093/jxb/ery422 30481315

[B12] Gilkerson J. Kelley D. R. Tam R. Estelle M. Callis J. (2015). Lysine residues are not required for proteasome-mediated proteolysis of the auxin/indole acidic acid protein IAA11. Plant Physiol. 168, 708–720. doi: 10.1104/pp.15.00402 25888615 PMC4453792

[B13] Greenham K. Santner A. Castillejo C. Mooney S. Sairanen I. Ljung K.. (2011). The AFB4 auxin receptor is a negative regulator of auxin signaling in seedlings. Curr. Biol. 21, 520–525. doi: 10.1016/j.cub.2011.02.029 21396817 PMC4295904

[B14] Guo F. Huang Y. Qi P. Lian G. Hu X. Han N.. (2021). Functional analysis of auxin receptor *OsTIR1/OsAFB* family members in rice grain yield, tillering, plant height, root system, germination, and auxinic herbicide resistance. New Phytol. 229, 2676–2692. doi: 10.1111/nph.17061 33135782

[B15] Hayashi K. Tan X. Zheng N. Hatate T. Kimura Y. Kepinski S.. (2008). Small-molecule agonists and antagonists of F-box protein-substrate interactions in auxin perception and signaling. Proc. Natl. Acad. Sci. U S A. 105, 5632–5637. doi: 10.1073/pnas.0711146105 18391211 PMC2291130

[B16] Huang C. Li Y. Wang K. Xi J. Xu Y. Si X.. (2022). Analysis of lipidomics profile of *Carya cathayensis* nuts and lipid dynamic changes during embryonic development. Food Chem. 370, 130975. doi: 10.1016/j.foodchem.2021.130975 34507207

[B17] Huang Y. Xiao L. Zhang Z. Zhang R. Wang Z. Huang C.. (2019). The genomes of pecan and Chinese hickory provide insights into *Carya* evolution and nut nutrition. GigaScience 8, giz036. doi: 10.1093/gigascience/giz036 31049561 PMC6497033

[B18] Iwase A. Mitsuda N. Koyama T. Hiratsu K. Kojima M. Arai T.. (2011). The AP2/ERF transcription factor WIND1 controls cell dedifferentiation in *Arabidopsis* . Curr. Biol. 21, 508–514. doi: 10.1016/j.cub.2011.02.020 21396822

[B19] Jogawat A. Yadav B. Lakra N. Singh A. K. Narayan O. P. (2021). Crosstalk between phytohormones and secondary metabolites in the drought stress tolerance of crop plants: a review. Physiologia Plantarum 172, 1106–1132. doi: 10.1111/ppl.v172.2 33421146

[B20] Kepinski S. (2007). The anatomy of auxin perception. Bioessays 29, 953–956. doi: 10.1002/bies.20657 17876776

[B21] Lavy M. Estelle M. (2016). Mechanisms of auxin signaling. Development 143, 3226–3229. doi: 10.1242/dev.131870 27624827 PMC5047657

[B22] Lee J. Kubota C. Tsao S. J. Bie Z. Echevarria P. H. Morra L.. (2010). Current status of vegetable grafting: Diffusion, grafting techniques, automation. Sci. Hortic. 127, 93–105. doi: 10.1016/j.scienta.2010.08.003

[B23] Loupit G. Brocard L. Ollat N. Cookson S. J. Lunn J. (2023). Grafting in plants: recent discoveries and new applications. J. Exp. Bot. 74, 2433–2447. doi: 10.1093/jxb/erad061 36846896

[B24] Luo J. Zhou J. Zhang J. (2018). Aux/IAA gene family in plants: molecular structure, regulation, and function. Int. J. Mol. Sci. 19, 259. doi: 10.3390/ijms19010259 29337875 PMC5796205

[B25] Mazur E. Kulik I. Hajný J. Friml J. (2020). Auxin canalization and vascular tissue formation by TIR1/AFB-mediated auxin signaling in *Arabidopsis* . New Phytol. 226, 1375–1383. doi: 10.1111/nph.16446 31971254 PMC7318144

[B26] Melnyk C. W. Gabel A. Hardcastle T. J. Robinson S. Miyashima S. Grosse I.. (2018). Transcriptome dynamics at *Arabidopsis* graft junctions reveal an intertissue recognition mechanism that activates vascular regeneration. Proc. Natl. Acad. Sci. U S A. 115, E2447–E2456. doi: 10.1073/pnas.1718263115 29440499 PMC5878008

[B27] Melnyk C. W. Schuster C. Leyser O. Meyerowitz E. M. (2015). A developmental framework for graft formation and vascular reconnection in *Arabidopsis thaliana* . Curr. Biol. 25, 1306–1318. doi: 10.1016/j.cub.2015.03.032 25891401 PMC4798781

[B28] Nan W. Wang X. Yang L. Hu Y. Wei Y. Liang X.. (2014). Cyclic GMP is involved in auxin signalling during *Arabidopsis* root growth and development. J. Exp. Bot. 65, 1571–1583. doi: 10.1093/jxb/eru019 24591051 PMC3967089

[B29] Ozga J. A. Jayasinghege C. P. A. Kaur H. Gao L. Nadeau C. D. Reinecke D. M. (2022). Auxin receptors as integrators of developmental and hormonal signals during reproductive development in pea. J. Exp. Bot. 73, 4094–4112. doi: 10.1093/jxb/erac152 35395070

[B30] Parry G. Calderon-Villalobos L. I. Prigge M. Peret B. Dharmasiri S. Itoh H.. (2009). Complex regulation of the TIR1/AFB family of auxin receptors. Proc. Natl. Acad. Sci. U S A. 106, 22540–22545. doi: 10.1073/pnas.0911967106 20018756 PMC2799741

[B31] Ping X. Ye Q. Yan M. Zeng J. Yan X. Li H.. (2022). Integrated genetic mapping and transcriptome analysis reveal the BnaA03.IAA7 protein regulates plant architecture and gibberellin signaling in Brassica napus L. Theor. Appl. Genet. 135, 3497–3510. doi: 10.1007/s00122-022-04196-8 35962210

[B32] Prigge M. J. Greenham K. Zhang Y. Santner A. Castillejo C. Mutka A. M.. (2016). The *Arabidopsis* auxin receptor F-box proteins AFB4 and AFB5 are required for response to the synthetic auxin picloram. G3 (Bethesda). 6, 1383–1390. doi: 10.1534/g3.115.025585 26976444 PMC4856089

[B33] Prigge M. J. Lavy M. Ashton N. W. Estelle M. (2010). Physcomitrella patens auxin-resistant mutants affect conserved elements of an auxin-signaling pathway. Curr. Biol. 20, 1907–1912. doi: 10.1016/j.cub.2010.08.050 20951049

[B34] Prigge M. J. Platre M. Kadakia N. Zhang Y. Greenham K. Szutu W.. (2020). Genetic analysis of the Arabidopsis TIR1/AFB auxin receptors reveals both overlapping and specialized functions. Elife 9, e54740. doi: 10.7554/eLife.54740 32067636 PMC7048394

[B35] Ruegger M. Dewey E. Gray W. M. Hobbie L. Turner J. Estelle M. (1998). The TIR1 protein of Arabidopsis functions in auxin response and is related to human SKP2 and yeast grr1p. Genes Dev. 12, 198–207. doi: 10.1101/gad.12.2.198 9436980 PMC316440

[B36] Salehin M. Bagchi R. Estelle M. (2015). SCF^TIR1/AFB^-based auxin perception: mechanism and role in plant growth and development. Plant Cell. 27, 9–19. doi: 10.1105/tpc.114.133744 25604443 PMC4330579

[B37] Sauer M. Balla J. Luschnig C. Wisniewska J. Reinöhl V. Friml J.. (2006). Canalization of auxin flow by Aux/IAA-ARF-dependent feedback regulation of PIN polarity. Genes Dev. 20, 2902–2911. doi: 10.1101/gad.390806 17043314 PMC1619939

[B38] Sharma A. Zheng B. (2019). Molecular Responses during plant grafting and its regulation by auxins, cytokinins, and gibberellins. Biomolecules 9, 397. doi: 10.3390/biom9090397 31443419 PMC6770456

[B39] Sheard L. B. Tan X. Mao H. Withers J. Ben-Nissan G. Hinds T. R.. (2010). Jasmonate perception by inositol-phosphate-potentiated COI1-JAZ co-receptor. Nature 468, 400–405. doi: 10.1038/nature09430 20927106 PMC2988090

[B40] Shu W. Liu Y. Guo Y. Zhou H. Zhang J. Zhao S.. (2015). A Populus TIR1 gene family survey reveals differential expression patterns and responses to 1-naphthaleneacetic acid and stress treatments. Front. Plant Sci. 6. doi: 10.3389/fpls.2015.00719 PMC4585115 26442033

[B41] Vidal E. A. Araus V. Lu C. Parry G. Green P. J. Coruzzi G. M.. (2010). Nitrate-responsive miR393/*AFB3* regulatory module controls root system architecture in Arabidopsis thaliana. Proc. Natl. Acad. Sci. U S A. 107, 4477–4482. doi: 10.1073/pnas.0909571107 20142497 PMC2840086

[B42] Wang J. Jiang L. Wu R. (2017). Plant grafting: how genetic exchange promotes vascular reconnection. New Phytol. 214, 56–65. doi: 10.1111/nph.14383 27991666

[B43] Wang Y. Shirogane T. Liu D. Harper J. W. Elledge S. J. (2003). Exit from exit: resetting the cell cycle through AMN1 inhibition of G protein signaling. Cell 112, 697–709. doi: 10.1016/S0092-8674(03)00121-1 12628189

[B44] Weerasak P. Sumie I. Masashi A. Shinobu S. (2014). ARF6 and ARF8 contribute to tissue reunion in incised Arabidopsis inflorescence stems. Plant Biotechnol. 31, 49–53. doi: 10.5511/plantbiotechnology.13.1028b

[B45] Yang Y. Huang Q. Wang X. Mei J. Sharma A. Tripathi D. K.. (2021). Genome-wide identification and expression profiles of *ABCB* gene family in Chinese hickory (*Carya cathayensis* Sarg.) during grafting. Plant Physiol. Biochem. 168, 477–487. doi: 10.1016/j.plaphy.2021.10.029 34757298

[B46] Yang Y. Mei J. Chen J. Yang Y. Gu Y. Tang X.. (2022). Expression analysis of *PIN* family genes in Chinese hickory reveals their potential roles during grafting and salt stress. Front. Plant Sci. 13. doi: 10.3389/fpls.2022.999990 PMC9557188 36247577

[B47] Yu H. Moss B. L. Jang S. S. Prigge M. Klavins E. Nemhauser J. L.. (2013). Mutations in the TIR1 auxin receptor that increase affinity for auxin/indole-3-acetic acid proteins result in auxin hypersensitivity. Plant Physiol. 162, 295–303. doi: 10.1104/pp.113.215582 23539280 PMC3641209

[B48] Yu H. Zhang Y. Moss B. L. Bargmann B. O. Wang R. Prigge M.. (2015). Untethering the TIR1 auxin receptor from the SCF complex increases its stability and inhibits auxin response. Nat. Plants.1 3), 14030. doi: 10.1038/nplants.2014.30 PMC4520256 26236497

[B49] Yuan H. Zhao L. Chen J. Yang Y. Zheng B. (2018). Identification and expression profiling of the *aux/iaa* gene family in chinese hickory (*Carya cathayensis* sarg.) during the grafting process. Plant Physiol. Bioch. 127, 55–63. doi: 10.1016/j.plaphy.2018.03.010 29549758

[B50] Zhao Y. (2010). Auxin biosynthesis and its role in plant development. Annu. Rev. Plant Biol. 61, 49–64. doi: 10.1146/annurev-arplant-042809-112308 20192736 PMC3070418

