# Supplementary material for: Role of TIR1/AFB family genes during grafting in Carya cathayensis
Source: Front Plant Sci. 2024 Nov 22;15:1494579. doi: 10.3389/fpls.2024.1494579 (PMC11622252; doi:10.3389/fpls.2024.1494579)
Supplement: Supplementary file 2 [file Table2.docx]

**Coding sequences of TIR1-AFB in related species**

>CcTIR1 CCA0981S0065

ATGCCGAGAATGGGGTACTCGTTCCCGGAGGAGGTGCTGGAGCATGTGTTCTCGTTCATACAGAGCGACGAGGACCGGAACGCGATCTCGGAGGTGTGCAGGTCGTGGCACGATATTGAGCGGTGGAGCAGGAGGCGGGTTTTCGTTGGCAACTGCTACGCCATCAGCCCCACGATGGTGATCCGACGGTTCCCCGAGGTTCGGTCCGTTAAGCTCAAGGGGAAGCCGCACTTTGCTGACTTCAACCTCGTCCCCGAAGACTGGGGAGGCTACGTGGCCCCCTGGATCTCTGCCATGGCCTCAGCCTACCCGTGGCTCGAAGAGATTCGGCTGAAACGTATGGTTATCATGGACGAGAGCTTGGAGCTCATTTCCAAGTCCTTCAAGAACTTCAAGGTCCTCGTGCTGTCGTCATGCGAGGGCTTCAGCACCGACGGCCTCGCCGCCATTGCTGCCAATTGCAGGAATTTGAGAGAACTGGACTTGGGGGAAAGTGAAGTGGAAGACCTGAGTGGGCACTGGCTGAGTCGTTTCCCTGATAACTACACCTCAATGGTGTCCCTTAACATTGCCTGCTTAGGGTGGGAGGTGAGTTTCTCTGCCTTGGAGCGCCTGGTGGGTAGGTGCCCCAACCTGAGGACTCTGCGGCTCAACCGTGCGGTGCCCCTCGACAAGCTTGCAAACCTACTTCGTCGAGCACCTCAGCTTGTTGAGTTGGGCACGGGGGCATACTCATCTGAGTTGCGACCTGATGTATTCTCAAACCTATCAGGAGCTTTCTCTGGATGCAAGGAACTTAAGAGCCTGTCTGGTTTTTGGGATGTGGTTCCAGGCTATCTTCCAGCTGTTTATCCTATCTGCTCCAAGTTAAAATCATTGAACTTGAGCTATGCTAACGTCCAGAGCCCTGAGCTTATCAAGCTAGTTAGCCAATGTCAGAACTTGCAGCGCTTATGGGTTCTGGATTACATTGAAGATGCTGGCCTTGATGCCCTTGCAGCATCTTGCAAGGATCTACGCGAATTGAGGGTATTTCCATCTGATCCGTTTGGACCAGAACCAAATGTGGCATTGACGGAACAGGGCCTTGTTTCTGTTTCTGAAGGCTGTCCTAAGCTCCAGTCGGTTCTCTACTTCTGCCGGCAAATGTCTAATGCAGCCTTAATGACTATTGCCAGGAATCGACCAAACTTTACCAGGTTTCGTCTTTGTATCATTGAGCCCCATACTCCTGATTACCTGACCCTTCAACCACTTGATGTGGGATTTGGAGCCATTGTGGAGCACTGCAAGGATCTACGACGTCTATCCCTTTCTGGTCTTCTCACTGATCGTGTGTTTGAGTACATTGGGACACATGCCAAAAAACTAGAGATGCTCTCCGTGGCTTTTGCTGGAGAAAGTGATTTGGGACTCCATCACATACTGTCAGGTTGTGATAATCTTAGGAAGCTGGAGATTAGGGACTGTCCCTTTGGTGACAAGGCTCTTTTGGCCAATGCTGCGAAGCTGGAGACAATGCGATCCCTTTGGATGTCTTCTTGCTCTGTAAGTTTTGGAGCATGTAAGCTGCTTGGTCAGAAGATGCCAGGGCTTAATGTTGAGGTTATTGATGAGAGGGGACCCCCAGATTCAAGACCGGAAAGCTGCCCCATTGAGAAGCTTTACGTATATAGGACAGTTTCTGGGCGAAGGTTTGACATGCCTGGATTTGTTTGGACAATGGCTGAAAATCCTGCATTGAGGCTTTCTTGA

>CcAFB1 CCA0535S0053

ATGCAGAGAATGGCCTGCTCTTTTCCGGACGAAGTGCTAGAGCATGTGTTCTCGTTCGTACAGTGCGACAATGACCGGAATGCGATCTCGACGGTGTGCAAGTCCTGGTACAAGATCGAACGGTGGAGCAGGAGGCGGGTCTTCGTTGGGAACTGCTACGCGATCAGCCCCGGTATGGTGATCCGACGGTTCCCGGATGTGCGGTCCATCGAACTCAAGGGGAAGCCACACTTTGCCGACTTCAACCTCGTCCCCGACGGATGGGGTGGCTACGTGGCCCCATGGATCTCCGCCATGGCGGCCGCTTACACTTGGCTCGAGGAGATTCGGCTTAAGCGTATGGTCATCACGGACGAGAACTTGGAGCTCATCTCCAAGTCCTTCAAGAACTTTAAGGTCCTCGTGCTGTCCTCCTGCGAGGGCTTCAGCACCGATGGCCTCGCCGCCATTGCTGCCAATTGCAGGAATCTGAGAGAGCTGGACTTGCGGGAGAGCGAAGTGGACGACCGGAGTGGGCACTGGCTCAAGCATTTTCCTGATGACTACACCTCACTGGTGTCCCTTAACATTGCCTGCTTAGGGTGTGAGGTGAGTTTCTCTGCCTTGGAGCGCCTAGTGGGTAGGTGCCCCAACCTGAGGACTCTTCGGCTCAACCGTGCTGTGCCCCTTGACAAGCTTGCAAACCTACTTCGTCGGGCACCTCAACTGGTTGAGTTGGGCACAGGGGCTTACTCGTCTGAGTTGCGACCTGATGTATTCTCCAACCTATCAGGAGCTTTTTCTGGATGCAAGGAACTGAAGAGCCTCTCTGGTTTTTGGGAAGTAGTCCCTGGGTATCTTCCAGCTGTTTACCCTATCTGCTGTAGGTTAACAACATTGAACTTGAGCTATGCTACTATCCAGAGCCCTGATCTCATCAAGCTAGTTGGCCAATGTCAGAATTTGCAGCGCTTATGGGTGCTGGATTACATTGAAGATGCCGGCCTTGATGCCGTTGCAGCATCTTGCAAGGATCTACGAGAATTGAGGGTGTTTCCATCTGATCCATTTGGACCAGAACCTAATGTACCGTTGACAGAACAGGGCCTTGTCTCCATTTCTGAAGGCTGCTCTAAGCTCCAGTCAGTTCTGTACTTTTGCCGTCAAATGTCTAATGCTGCCTTGATGACCATTGCCAGGAATCGACCTAACATGACCAGGTTTCGTCTTTGTATTATTGAGCCCGGAACTCCTGATTACCTTACCCTTCAACCACTTGATGTGGGATTTGGAGCCATTGTTGAGCACTGCAAGGATCTACAACGGCTTTCCCTTGCTGGTCTTCTCACCGATCGTGTGTTTGAGTACATTGGGACTTATGCCAAAAAATTGGAGATGCTGTCTGTGGCCTTTGCTGGAGAAAGTGATTTGGGACTCCATCACGTGCTGTCTGGTTGTGAAAACCTTAGGAAGCTGGAGATTAGGGACTGCCCCTTTGGTGACAAGGCTCTTTTGGCCAATGCTGCAAAGCTGGAGACAATGCGATCCCTTTGGATGTCTTCTTGCTCTGTGACTTTCGGAGCATGTAAGCTACTAGGTCAGAAGATGCCGAGGCTTAATGTAGAGGTTATTGATGAGAGTGGACCCCCAGATTCAAGACCAGAAAGCTGCCCTATCGAGAAGCTTTACATATATAGGACTGTTGCTGGGTCAAGGTTTGACATGCCTGGTTTTGTTTGGACGATTGATGAAGATTCGGCGATGAGGCTTTCTTGA

>CcAFB2 CCA1250S0070

ATGAATTATTTTCCGGACGAGGTTTTAGAGCACGTTTTCGATTTCTTAACGTCACACAGGGACCGGAACGCGGTATCTCTAGTGTGCAGTTTATGGTACAGAGTAGAAAGGTTTAGTAGGCAGAGAGTCTTCATAGGAAATTGTTATGCAATCAGTCCCGAGAAACTGATCACGAGGTTTCCGGGACTCAAGTCGCTAACTTTGAAGGGGAAGCCTCATTTCGCGGACTTCAACTTGGTGCCGTACGATTGGGGCGGTTATGTGGAGCCTTGGATCGAAGCCTTGGCAAAGAGTAGGATTGGTTTGGAAGAGCTTAGGCTTAAGAGGATGATGGTCTCGGATGAGAGCCTCGAGCTTCTTTCGAGGTCTTTCGCCAATTTTAAAACTTTGGTGCTTGTTAGCTGTGAAGGTTTCACCACCAATGGCCTTGCAGCTATAGCTGCTAACTGTAGGTTTCTTAGGGAGTTGGACTTGCAAGAAAATGAAATTGATGACCATAGTGGTCACTGGCTTAGTTGCTTTCCTGACAGCTGCACATCACTCACCTCCCTGAATTTTGCTTGCCTCAAAGGAGAAATTAATTCTGCATCCCTTGAGAGACTTGTGGCAAGATCTCCTAATCTCAGGAGTTTGAGGTTAAACCGTTCTGTGCCTCTTGAGACGCTCCAAAAGATACTGGTGCGAGCACCTTTACTAGTGGATTTAGGGACGGGTTCATATGTCCATGATCCTGATTCTGAGACTTACAATAAACTAAAGACTACCTTTCTGAATTGTAAATCAATCAGAAATTTATCAGGGTTTTTGGAGGTTGCTCCTCGCTGCCTGCCAGCCATTTTCCCTATTTGCTTGAACCTGACCTACTTGAACCTGAGCTATGCTGCAGGGATTCCCGGTTCTGAGCTTATAAAACTAATTGGTCGTTGTGCAAAACTTCAGCGCCTATGGATACTGGATTGTATTGGAGACAAGGGACTAGAAGTTGTGGCTTCCACTTGTAATGAACTGCAGGAATTGAGGGTTTTCCCATCTGATCCCTTAGGGATTGGGCATGATGCCGTAACAGAAAATGGCCTGGTTGCTATATCCATTGGTTGCCCAAAGCTTCATTCATTGCTATACTTCTGTCAGCAGATGACAAATGCTGCTCTCATAACCGTAGCAAGGAACTGTCCAAATTTTATTCGCTTTAGGCTGTGCATACTCGATCCTACCAAACCTGACGCTGTGACCATGCAGCCTTTAGATGATGGTTTTGGAGCAATTGTCCAGTCATGTAAGCTTCTTCGGCGGTTATCACTTACTGGCCTTCTTACTGACCAGGTTTTCCTTTACATTGGGATGTATGCTGAGCAGCTTGAAATGCTCTCTGTTGCATTTGCTGGCAATAGCGACAAGGGAATGCTCTACGTGTTGAACGGGTGCAAGAAGCTTCGCAAGCTTGAGATAATGGACTGCCCCTTTGGTAACAGGGCACTTCTATCGGACGTGGGAAAGTATGAAACAATGCGATCCCTTTGGATGTCGTCCTGTGAAGTAACTCTTTGGGGCTGCAAGACACTTGCAAAGATGATGCCAAGGCTTAATGTGGAGATAATAAACGAACATGATCAGATGGAAATTTGTCCCGAGGATGATGGGCAAAGGGTAGAAAAGATGTACCTGTACCGTACACTGGTTGGGCCAAGGAAAGATGCACCGGAATTTGTGTGGAATTTATAG

>CcAFB3 CCA1539S0035

ATGAACTATTTTCCGGACGAGGTTTTAGAGCACGTGTTCGATTTCGTAGCATCGCATAGGGACCGCAACGCGGTATCTCTGGTGTGCAGATTATGGCATAGAGTAGAAAGGCTTAGTAGGCAGAGAGTGTTCGTCGGAAACTGCTATGCACTCAATCCCGAGATACTGATCGCGAGGTTTCCGGGTCTCAAGTCGCTGACTTTGAAGGGGAAGCCTCATTTTGCGGACTTCAACTTGGTGCCGCATGACTGGGGAGGCTATGTGCAACCTTGGATCGAGGCCTTGTCAAATAGTAGGATTGGCTTGGAAGAGCTTAGACTGAAGAGAATGGTGGTCTCGGATGAGAGCCTCGAGCTTCTTTCGCGGTCTTTCGCGAATTTTAAGTCTTTAGTGCTTGTTAGCTGTGAAGGGTTCACCACCGTTGGCCTTGCAGCTATAGCTGCTAACTGTAGGTTTCTTAGGGAGCTGGACCTGCAAGAAAATGATATTGACGATCATAGTGGCCACTGGCTTAGTTGCTTTCCTGACAGCTGCACATCACTCATCTCCCTGAATTTTGCGTGCCTCAAAGGAGAAATTAATTTAGCAGCGCTTGAGAGACTTGTGGCAAGATCTCCTAATCTCAGGAGTTTGAGGTTAAATCGTGCGGTGCCTCTTGAGACGCTCCAAAATATATTGATGCGAGTGCCTCAACTAGTGGATTTAGGGACAGGATCATATATCCATGATCCTGATTCAGAGACCTACAATAAACTCAAGCATACCATTCTGAAGTGTAAATCAATTAGGAATTTATCAGGGTTTTTGGAGGTTGCTCCTCGCTGCCTGCCAGCCATTTACCCTATCTGCTTGAATTTGACCTCCTTAAACCTAAGCTACGCTGCAGGGATTCATGGTTCTGAGCTTGTAAAGCTAATTCGGCACTGTGCAAAACTTCAGCGCCTGTGGATACTGGATTGTATTGGAGACAAGGGACTAGAAGTCGTAGCTTCCACTTGTAAAGAGTTGCAGGAATTGAGGGTTTTCCCGTCTGATCTACTTGGGGTCGGGCATGCTGCTGTGACAGAAAATGGCCTGGTTGCTATATCTGTTGGTTGCCCAAAGCTTCATTCATTGCTGTACTTCTGCCAGCAGATGACAAATGCTGCTCTCATAGTCGTAGCCAAGAACTGTCCAAATTTTATCCGCTTCAGACTCTGCATTCTTGAACCTACAAAACCTGACCCTGTGACCATGCAGCCTTTAGATGATGGCTTTGGAGCAATTGTCCAGTCATGCAAGCGTCTTCGACGATTGTCGCTATCTGGCCTTCTGACTGACCGGGTTTTCCTTTACATTGGGATGTATGCCGAGCAGCTTGAAATGCTCTCAGTCGCATTTGCTGGGGACAGTGACAAGGGCATGCTTTACGTGTTAAATGGGTGCAAGAAGCTTCGCAAGCTTGAGATCAGGGACTGCCCGTTTGGTGACAAGGCACTTCTAACGGACGTGGGAAAGTATGAAACAATGCGATCCCTTTGGATGTCGTCCTGTGAAGTTACTCTTGGAGGCTGCAAGACACTTGCGAAGGGGATGCCTAGGCTTAATGTGGAGATCATAAATGAACATGATCAGATGGAAATTGGCTCTGAAGAGCAGAGAGTAGAGAAGATGTATCTGTATCGGACACTGGTCGGGCCAAGGAAAGATGCACCGGAATTTGTGTGGACATTGTAG

>CcAFB4 CCA0533S0235

ATGAGAAAGGACCGGACGGAAATGTCCGAAGACGACGATCGGTCCCCTCCCTTGGATCTTCGCGGGGCGGACATCGCCGAGTCCTCCAACAAGACCCGGAACTGTACCGGAGTGTCCGGTTCTGGGGGTCCTGTACCGGGACCCGGTCCCAGTTCCATGGAGTTTCAGGCCCCCTACCCGGACCAAGTGCTCGAGAACGTGCTCGAAAACGTGCTCCAGTTCCTCAGCTCCCGGCGGGACCGTAACGCGGCCTCATTGGTATGCAGGTCGTGGTGGCGCGTGGAGGCGCTCACCCGATCCGACCTCTTCATCGGCAACTGCTACTCGGTCTCACCACGGCGGGCCACGGCCCGGTTCACCCGGGTACGGTCCGTCTCCATCAAGGGAAGGCCGAGGTTCGCGGACTTCAACCTGATGCCGCCCGATTGGGGGGCTCACTTCGCCCCTTGGGTGACGGCCATGGCGTCTGCCTATCCCTGGCTAGAGAAGGTTTACCTAAAGCGCATGTCCGTCACGGACGACGATCTGGCCCTACTTGCCGAGTCCTTCCCCTCCTTCAAAGAGCTCGTTCTCGTCTGCTGCGATGGCTTCGGTACCAGTGGCCTCGCCGTCATCGCTAGCAAGTGCAGACAACTGAAAGTGCTTGATCTGATCGAATCCGAGGTCACGGATGATGAGGTCGATTGGATTGCTTGTTTTCCGGAGAGTGGAGCGACCTGTCTGGAGTCACTGATCTTCGATTGCGTAGAATGTCCAATAAATTTTGATGCATTGGAGAGGCTGGTGGCTAGGTCCCCATCCTTGAAGAAGCTTAGGTTGAATCGTTTTGTTTCGATTGGGCAGCTATATCTCCTGATGGTACGTTCTCCGCAGCTCACACACCTTGGAACTGGCTCTTTTAGTGCACCAGAGGGCATGGCTCAGGGTGAACAAGAACCCGACTTTTTCACTGCCTTTGCTGCTTGCAGATCCTTAGTTTGTCTTTCGGGATTCAGGGAAATCTTGCCAGATTACTTACCAGCCATCCACCCAGTCTGTGCTAATCTTACCTCCCTGAATTTCAGCTATGCGAATATTAATGCAGAGCAACTCAAATTAGTCATATTTCACTGCCACAAACTCCAGATTTTCTGGGTCCTTGATTCAATATGCGATGAAGGACTTCAAGCTGTGGCTTCAACCTGCAAGGACTTGCGTGAGCTTCGTGTTTTCCCTTTTGACGCTCGGGAGGATAATGAGGGCCCTGTTTCTGACTTGGGCCTCCTAGCAATTTCTGAGGGCTGTAGAAAATTACAATCGATTTTGTATTTCTGCCAGCATATGACAAATGCGGCAGTGATAGCCATGTCAAGGAATTGCCCAGATCTTGAGGTTTTCCGTCTCTGTATAATGGGGCGACATCGCCCGGACCGTGTGACCAATGAACCCATGGATGAAGGTTTTGGAGCCATTGTTATGAACTGTAAGAAGCTAACTAGGCTTGCTGTATCCGGTTTACTGACTGATCGTGCTTTCAATTATATTGGAAAATATGGGAAACTGGTTAGAACCCTTTCAGTTGCTTTTGCTGGAGACAGTGACATGGGGCTTAAATATGTGCTAGAGGGCTGCCCTAGACTGCAGAAGCTTGAGATCAGGGATAGCCCATTTGGGGATGCAGCTTTGCGTTCTGGTTTGCATCACTATTACAACATGAGGTTCCTTTGGATGTCTTCATGCAGGTTATTTCCCCAAGGTTGTCAGGAGATTGCTCGAGCATTGCCCCGCCTGGTGGTGGAAGTAATCAGGGATGATGCTATTGAGCACACAGATGAGACTGTTGACATATTGTACATGTATCGGTCTCTTGAGGGCCCCAGGGATGATGCCCCAGGATTTGTGACCATCATGCAATAA

>CcAFB5 CCA1097S0042

ATGAGGAAGGACCGGACTGGAATTTCACAAGACGACGATCGGTACCCGCCGGCGGATCTTCGCGGGGCCGAATCTTCCAACAAGACCCGGAACTGCTGTGGGGTGGGGTCCGTGTCAGGGAGTACTGGGCCGATTTCCATGGAGTTCCAGGCCCCGTGCCCGGACCAAGTGCTGGAGAACGTGCTGGAAAACGTGCTCCAGTTCCTCAGCTCCCGGCGGGACCGGAACGCGGCCTCATTGGTGTGCAAGTCTTGGTGGCGCGTCGAGGCTCTCACCCGATCCGACCTCTACATCGGCAACTGCTACTCAGTCTCACCCAGGAGGGCCATGAACCGCTTCTCTCGTGTCCAGTCCGTATCCATCAAGGGCAGACCGAGGTTCGCGGACTTCAACCTGATGCCGATCGATTGGGGGGCCCACTTCGCCCCGTGGGTGACGGCCATGGCGTCCGCTTATCCTTGGCTGGAGAAGGTGTACCTGAAGCGCATGTCCGTCACGGACGACGATCTGGCCCTCCTCGCCGACTCCTTCCCCTCCTTCAAAGAGCTCGTTATCGTCTGCTGCGATGGCTTCAGTACGAGTGGCCTCGCCCTCGTCGCGAGCGACTGCAGACAACTGAAAGTGCTTGATCTGATCGAATCTGAGGTCACGGATGATGATGTTGATTGGATAGCTTGTTTCCCGGAGAGCGGAGCGACTTGTCTGGAGTCGCTGATTTTTGATTGCGTTGAATGCTCAATTAATTTTGACGCATTAGAGAGTCTGGTGGCTAGGTCCCCGTCCTTGAAGAAGCTTAGGTTGAACTGCAATGTTTCAATTGGGCAGCTATGCCGCCTGATAGTACGAGCTCCGCAGCTCACACACCTTGGAACTGGTTCGTATAGTTCCCCAGACGATGCAGTTCCGGGTGAACATGCTTCGGACCTTCAAAGTGCCCTTTCTGCTTGCAAATCCTTAGTTTGTCTCTCAGGATTCAAGGAAATGTTGCCAGATTACTTACCGGCCATCTACCCTGTCTGTGCTAATCTTACCTCTCTGAATTTTAGCTATGCAAATATTCATGCAGATCAAATTAGATCGGTCATATTTCACTGCCACAATCTCCAGGTATTCTGGGTCCTTGATTCAATACGAGATGAAGGACTTCAAGCTGTGGCTTCAACCTGCAAGGACCTGCGTGAACTTCGTGTTTTCCCTCTCGATGCTCGGGGGGATAATGAGGGCCCTGTTTCTGATGTGGGCCTCCTAGCAATTTCCGAGGGTTGTAGGAAACTGCGATCCATTTTGTATTTCTGCCAGCATATGACAAATGCAGCAGTGATAGCCATGTCAAGGAACTGCCCGGATCTTGAGGTTTTCCGTCTCTGTATAATTGCTCTACATAGCCCTGACCATGTGACGGGGAAACCCATGGATGAAGGTTTTGGAGCCATCGTTATGAACTGTAAGAAGTTAACTCGGCTTGCTGTATCCGGTTTACTGACTGATCAAGCTTTTGGTTATATTGGAAAATATGGGAAATTGCTTCGAACGCTGTCAGTTGCTTTCGCTGGAGATAGTGACATGGGGCTTAAATATGTGCTTGAGGGCTGCCCTAGACTGCAAAAGCTTGAGATCAGAGATAGCCCATTTGGGGATGCAGCTTTGCATTCTGGTTTACATCACTACTACAACATGAGATTCCTCTGGATGTCGTCATGTAGGCTATCTCCCAAAGGTTGTCAGGAGATTGTTCGAGCATTGCCCCGTCTGGTGGTGGAAGTAATCAAGCATGATGTTGCAGTGTGCCCAGATGATACTGTTGACATTTTATACATGTATCGGTCTCTTGAAGGACCAAGGGATGATGCCCCAAAAGTTGTGACCATCTTGCAATAA

>CcAFB6 CCA0729S0024

ATGGATCCTGAGAGGAAGAAGGTTCTGGAACGAGTGGCGATCTCGACGTTCCCGGACGAGGTTCTGGAACGAGTTCTGGTTCAGTTGAAGTCCCACAAAGACCGGAGCTCCGTCTCTTTGGTCTGCCACAGCTGGTACGACGCCGAGCGGTTTTCACGGACCCACGTGTTCATTGCGAATTGCTACTCGGTGACGCCAGAGATCGTGTCCCGGCGTTTCCCGAACATCCGGAGCGTCAGTTTAAAGGGGAAGCCCCGGTTCTCGGACTTCAATCTGGTTCCTCCGAAGTGGGGCGCCGATATTCGGTCTTGGCTGGTGATGTTTGCGGCTAAGTACCCGTTTCTGGAGGAGCTGAGGCTCAAGAGGATGACCGTTACCGATGAGAGCTTGGAGTTTCTGGCTCTTTCTTTTCCGAATTTCAAGGGTCTTTCGCTTTTGAGCTGTGATGGCTTCAGTACTGGCGGGCTCGCAGCGATTGCTACTCACTGCAAGAATTTAACCGAACTGGACATACAGGAGAATGACCTCAACGATAAAAGTGGCAGTTGGTTGAGTTGCTTCCCTGAAAACTTCTCCTCATTGGAAGTACTAAACTTTGCCAATTTAAGTAATGACGTTAATTTTGAAGCCCTTGAGAGACTGGTTAGTAGGTGCAAATCGTTGAAGGTTTTGAAGGTCAATAGAAATATAACCTTGGAACAATTGCCAAGGCTGCTTATTCATGCACCACTATTAACAGAGCTTGGTACTGGCTCATTTTTGCAAGAGCATCCAGCCTGTCATTACTCTGAGCTCGAAAATGCCTTCAACAAATGCATGAATCTAAACACCCTCTCTGGTTTGTCAGAAGTTACTGCCTTGTCTCTCCCAGCTCTATACCCTGCCTGTGCAAATTTGACTTTCTTGAATTTGAGCTGCGCTGCTTTGCAAAGTGATGAACTTGCTAAACTTCTTGTTCATTGCCCATATCTTCGTCGCCTCTGGGTCCTGGACACAGTCGAAGACAAGGGTTTGGAGGCTGTTGGATCCAACTGTCCTCTGCTTGAGGAACTCCGTGTCTTCCCTACTGATCCATTTGACGAGGATACTTTCCATGGAGTGACTGAATCTGGGTTCATTGCTGTCTCTTATGGCTGCAGGAGACTAAGCTATGTTCTCTACTTTTGCCGCCAGATGACTAACGCTGCTGTAAAAACCATTGTGCAGAACTGCCCTGATTTCACTCACTTCAGGCTTTGCATAATGAATCCATGGCAGCCAGATTACCAGACCAATGAGCCTATGGATGAGGCTTTCTGTGCGGTGGTGAAAACTTGCACTAAGCTTCAGAGGCTTGCAGTTTCAGGCTTATTGACTGACCTGACTTTTGAGTACATTGGGAAATATGCCAAGAACTTGGAAACCCTGTCGGTTGCTTTTGCCGGCAGCAGTGATTGGGGTATGCAGTGTGTATTGGGAGGTTGTCCAAAACTGAGGAAACTTGAAATAAGGGACTGCCCATTTGGGAATGCAGCACTGCTATCGGGCTTGGATAAATATGAATCCATGAGATCCCTATGGATGTCAGACTGCAAAGTAACAATGAATGGATGTCGATTGTTGGCAAAAGAGATGCCCAGGTTGAATGTAGAGGTGATAAAGGTTGATGGGAGTAATGAGAGTCAAGCTGATAAAGTTTATGTTTATCGTTCTGTTGCTGGACCAAGAAGGGATGCCCCACCTTTCGTTCTCACCCTCTGA

>AtTIR1

ATGCAGAAGCGAATAGCCTTGTCGTTTCCAGAAGAGGTACTAGAGCATGTGTTCTCGTTTATTCAGCTGGATAAGGATAGGAACTCAGTCTCTCTGGTGT

GCAAGTCATGGTACGAGATCGAGCGGTGGTGCAGGAGGAAAGTCTTCATCGGGAACTGCTACGCCGTGAGTCCAGCGACGGTGATTAGGAGGTTCCCGAA

AGTGAGATCCGTGGAGCTTAAAGGAAAACCTCACTTTGCTGACTTTAATTTGGTACCTGACGGATGGGGAGGTTACGTGTATCCATGGATTGAGGCCATG

TCTTCGTCTTACACGTGGCTTGAAGAGATAAGGCTGAAGAGGATGGTGGTCACCGACGATTGCTTGGAGCTCATAGCCAAGTCTTTTAAGAATTTTAAGG

TTCTTGTGCTTTCTTCCTGCGAAGGCTTCTCCACCGATGGTCTCGCTGCTATCGCTGCCACTTGCAGGAATCTGAAAGAGCTTGACTTACGAGAGAGTGA

TGTTGACGACGTTAGTGGCCACTGGCTTAGCCATTTCCCAGATACATACACTTCTTTGGTATCACTCAATATATCTTGCTTAGCATCTGAGGTCAGTTTC

TCTGCTCTGGAAAGGCTGGTGACTAGGTGTCCCAATCTCAAGTCTCTCAAGCTTAACCGAGCTGTTCCACTTGAAAAATTGGCTACTTTACTTCAAAGAG

CACCTCAATTGGAGGAATTGGGCACTGGTGGGTACACTGCAGAAGTGCGACCAGATGTTTACTCTGGTTTATCTGTAGCGCTCTCTGGGTGCAAGGAATT

GAGGTGCTTATCTGGATTTTGGGATGCTGTTCCTGCCTATCTTCCAGCAGTTTATTCGGTTTGCAGTCGGCTTACAACTTTGAATCTGAGTTATGCAACA

GTCCAGAGCTATGATCTTGTCAAGCTTCTTTGTCAATGCCCTAAACTGCAGCGCCTCTGGGTGCTTGACTACATCGAGGATGCTGGTCTTGAGGTGCTTG

CTTCAACCTGCAAGGACCTACGCGAGCTGAGAGTGTTTCCGTCCGAGCCTTTTGTCATGGAACCAAATGTGGCATTGACGGAACAGGGGCTTGTCTCCGT

CTCCATGGGCTGTCCAAAACTCGAGTCGGTTCTCTACTTCTGCCGTCAAATGACCAATGCTGCATTGATAACCATTGCTAGGAACCGTCCCAACATGACT

CGCTTCCGTTTGTGCATCATTGAGCCAAAAGCCCCAGACTATCTGACTCTAGAGCCACTGGATATTGGATTTGGAGCCATAGTAGAGCACTGCAAGGATC

TCCGTCGCCTCTCTCTATCTGGCCTCTTGACCGACAAGGTTTTTGAATACATTGGGACATATGCCAAGAAGATGGAAATGCTCTCAGTGGCATTTGCAGG

AGACAGTGACTTAGGCATGCATCATGTTTTGTCCGGGTGCGATAGCTTGAGGAAACTAGAGATAAGGGACTGCCCGTTTGGAGACAAGGCGCTTTTGGCC

AATGCTTCAAAGCTGGAGACAATGCGATCCCTTTGGATGTCTTCTTGTTCCGTGAGTTTTGGAGCCTGCAAGTTACTAGGACAGAAGATGCCAAAGCTGA

ATGTGGAAGTCATCGATGAACGGGGTGCACCGGACTCGAGACCAGAGAGCTGCCCTGTTGAGAGAGTCTTCATATACCGAACAGTGGCTGGTCCTCGATT

TGACATGCCTGGCTTCGTCTGGAACATGGACCAAGACTCAACAATGAGGTTTTCCAGGCAAATCATTACTACTAACGGATTATAA

>AtAFB3

ATGAATTATTTCCCAGACGAGGTTATAGAGCACGTGTTTGACTTCGTAGCTTCTCACAAAGACAGGAACTCGATATCTCTGGTCTGCAAATCATGGCACA

AGATCGAGAGGTTTAGTAGGAAGGAAGTGTTCATCGGAAACTGCTACGCGATTAACCCGGAGAGGTTGATCAGGAGGTTTCCATGTCTCAAATCCTTAAC

TTTAAAAGGGAAGCCTCATTTTGCAGACTTCAACTTGGTTCCTCATGAATGGGGAGGTTTCGTGCATCCTTGGATTGAAGCTTTGGCTAGAAGCCGTGTG

GGACTTGAGGAGCTGAGGTTGAAGCGGATGGTTGTAACAGATGAAAGCTTGGACCTTCTTTCACGTTCTTTTGCAAATTTCAAGTCTTTGGTTCTTGTTA

GCTGTGAAGGGTTTACCACTGATGGCTTAGCTTCCATTGCCGCTAATTGCAGGCATCTTCGTGAGCTGGACTTGCAAGAGAATGAGATTGATGATCATAG

AGGTCAATGGCTGAACTGTTTTCCAGATAGCTGCACTACTCTTATGTCGTTGAATTTCGCTTGCCTTAAAGGAGAGACCAATGTTGCTGCTTTAGAAAGG

CTTGTTGCTAGGTCACCAAACCTGAAGAGCTTGAAGTTAAACCGTGCAGTACCGCTTGACGCACTCGCAAGGTTAATGAGTTGTGCGCCGCAGCTAGTGG

ACTTAGGAGTAGGGTCTTATGAGAATGAGCCAGATCCTGAATCTTTTGCAAAACTCATGACTGCCATTAAGAAATACACATCGTTAAGGAGCTTGTCTGG

CTTTTTAGAGGTTGCTCCACTCTGCCTCCCAGCGTTCTACCCAATTTGCCAAAACCTTATCTCTTTGAACCTCAGCTATGCAGCTGAAATCCAAGGCAAC

CACCTCATTAAGCTTATTCAGCTTTGCAAGAGACTTCAACGATTATGGATATTGGATAGTATTGGTGACAAAGGACTTGCGGTTGTCGCTGCCACATGTA

AAGAGTTACAAGAGCTTAGAGTTTTTCCCTCTGATGTACATGGTGAAGAAGATAACAACGCATCTGTGACTGAGGTTGGACTAGTCGCCATTTCCGCAGG

TTGCCCTAAACTTCATTCGATTCTGTACTTCTGCAAACAGATGACAAACGCAGCGCTCATAGCCGTGGCCAAAAACTGTCCAAACTTCATCCGGTTCAGG

CTATGCATTCTCGAGCCACACAAACCTGACCACATTACATTTCAATCACTGGACGAGGGCTTTGGTGCAATCGTACAAGCTTGCAAGGGTCTAAGACGGC

TCTCTGTCTCCGGTCTCTTAACCGATCAAGTCTTTCTCTACATCGGTATGTACGCGGAACAGCTCGAGATGCTTTCGATAGCTTTTGCGGGGGACACTGA

CAAAGGAATGCTCTATGTGTTGAATGGATGCAAAAAAATGAGGAAGCTGGAGATAAGGGACAGTCCTTTTGGGAACGCTGCGCTTCTTGCTGACGTGGGT

AGGTACGAAACAATGCGATCCCTTTGGATGTCGTCTTGTGAAGTAACACTCGGTGGCTGCAAGAGGCTCGCGCAGAATTCGCCACGGCTTAACGTAGAGA

TCATCAACGAGAATGAGAATAATGGGATGGAACAGAATGAAGAAGATGAAAGAGAGAAGGTTGATAAACTTTACCTCTACCGAACAGTGGTTGGGACTAG

AAAAGATGCACCACCATATGTTAGGATTCTTTAG

>AtAFB2

ATGAATTATTTCCCAGATGAAGTAATAGAGCATGTATTCGACTTTGTAACATCTCACAAAGACAGGAATGCTATATCTCTTGTATGCAAATCATGGTACA

AGATTGAAAGATACAGTAGGCAAAAGGTTTTCATTGGAAACTGTTATGCCATTAATCCAGAGAGGTTGCTTCGGAGATTCCCATGTCTAAAGTCTTTGAC

TTTGAAAGGAAAACCTCATTTTGCGGATTTCAATTTGGTTCCTCATGAATGGGGAGGTTTTGTGCTACCTTGGATTGAGGCTTTGGCTAGAAGCCGTGTA

GGACTTGAAGAGCTTAGGTTGAAGAGGATGGTTGTTACTGATGAGAGTCTTGAGCTGCTTTCTCGTTCTTTTGTCAATTTTAAGTCTTTGGTCCTTGTTA

GCTGTGAAGGTTTTACCACTGATGGTCTTGCCTCTATTGCCGCTAATTGCAGGCATCTTCGGGATCTTGATTTGCAAGAGAATGAAATCGATGATCATAG

AGGTCAATGGTTAAGTTGTTTCCCAGACACTTGCACGACTCTTGTCACGCTAAACTTTGCGTGCCTCGAAGGAGAAACTAATCTGGTTGCTCTAGAGAGG

CTTGTTGCTAGGTCTCCAAACCTAAAGAGTCTGAAGCTAAATCGTGCAGTACCGTTAGATGCACTCGCAAGGTTAATGGCGTGTGCGCCGCAGATAGTTG

ACTTAGGAGTAGGGTCTTATGAGAATGACCCAGATTCCGAGTCTTACTTGAAACTCATGGCTGTCATAAAGAAATGCACCTCGTTGAGGAGTTTGTCGGG

TTTTCTAGAGGCTGCTCCTCACTGTCTCTCAGCTTTCCACCCAATATGTCATAACCTCACCTCCTTGAATCTTAGTTACGCAGCTGAGATTCATGGTAGC

CACCTTATTAAGCTTATTCAGCATTGCAAGAAACTTCAGCGGTTATGGATTTTGGATAGTATAGGTGACAAAGGGCTTGAAGTTGTAGCTTCTACATGTA

AAGAGTTACAAGAGCTTAGGGTTTTTCCATCTGATTTACTCGGTGGAGGCAACACAGCTGTGACCGAAGAAGGTCTAGTTGCCATCTCGGCAGGCTGCCC

TAAGCTCCACTCTATACTCTACTTCTGCCAACAAATGACAAACGCAGCTCTCGTAACCGTTGCCAAGAACTGTCCAAATTTCATCCGTTTCCGACTCTGC

ATCCTCGAGCCAAACAAACCCGATCACGTCACATCTCAACCTCTAGACGAAGGCTTTGGAGCAATCGTCAAAGCCTGCAAGAGCCTGAGAAGGCTTTCTC

TCTCAGGTCTCCTTACAGACCAAGTCTTCCTCTACATCGGAATGTACGCGAATCAGCTCGAGATGCTCTCCATAGCCTTTGCAGGAGATACAGACAAAGG

CATGCTATATGTGTTGAATGGTTGCAAAAAGATGAAGAAACTAGAGATAAGGGATAGTCCGTTTGGGGACACGGCGCTTCTTGCTGATGTGAGCAAGTAT

GAAACAATGCGATCCCTTTGGATGTCTTCATGTGAAGTCACACTCAGTGGATGCAAAAGGCTCGCAGAGAAAGCGCCATGGCTCAATGTAGAGATCATAA

ACGAGAATGATAATAACCGGATGGAAGAAAACGGACACGAGGGGAGGCAGAAAGTGGATAAGTTGTATCTGTACCGGACTGTGGTTGGGACAAGAATGGA

TGCGCCGCCATTTGTGTGGATTCTCTAA

>AtAFB1

ATGGGTCTCCGATTCCCACCTAAGGTGTTGGAACATATCCTCTCCTTCATTGATTCCAACGAGGACCGGAACTCTGTTTCTCTGGTCTGCAAGTCATGGT

TTGAGACAGAACGGAAGACTAGGAAGCGAGTCTTTGTCGGAAACTGTTACGCGGTCAGTCCTGCTGCGGTTACACGACGGTTCCCGGAGATGAGATCTTT

GACTTTGAAGGGGAAGCCACACTTCGCTGACTATAATCTCGTTCCTGATGGTTGGGGTGGTTATGCTTGGCCGTGGATTGAAGCTATGGCGGCGAAAAGT

TCGTCTCTTGAAGAGATCAGAATGAAGAGGATGGTGGTGACTGATGAGTGCTTAGAGAAAATTGCTGCTTCGTTTAAGGATTTTAAAGTCCTTGTGTTGA

CTTCTTGTGAAGGTTTCTCTACTGATGGTATCGCTGCTATTGCAGCTACTTGCAGGAACTTGAGAGTGTTGGAACTACGAGAGTGTATTGTTGAAGATTT

AGGAGGAGATTGGCTTAGCTATTTTCCAGAGAGTTCAACTTCTTTGGTCTCTCTTGACTTCTCTTGTTTAGATTCTGAGGTTAAAATCTCGGATTTAGAG

CGTCTTGTGAGCAGATCTCCAAACTTGAAGTCTTTGAAGTTGAATCCAGCTGTGACTCTAGATGGACTCGTTAGCTTACTTCGTTGTGCTCCACAACTGA

CTGAGCTCGGCACAGGTTCTTTCGCAGCTCAATTGAAACCTGAAGCGTTTTCAAAGTTATCAGAAGCTTTTTCAAACTGTAAGCAACTTCAGAGCTTATC

TGGTCTCTGGGATGTCCTCCCTGAATATCTTCCAGCTCTTTATTCTGTCTGTCCTGGTCTTACCTCGTTGAACTTGAGCTACGCTACTGTCCGAATGCCT

GATCTTGTTGAGCTTCTTAGGCGATGCTCGAAACTGCAGAAGCTATGGGTGATGGACTTGATTGAGGACAAAGGTCTTGAAGCTGTTGCCTCATATTGCA

AGGAACTGCGAGAACTGAGGGTGTTTCCATCTGAGCCAGATCTTGATGCAACCAACATACCTCTGACGGAACAAGGCCTGGTCTTTGTGTCTAAAGGCTG

TCGAAAGCTTGAGTCTGTTCTCTACTTCTGTGTCCAGTTCACAAACGCAGCTTTGTTTACCATAGCAAGAAAACGTCCGAATCTCAAGTGCTTCCGTCTC

TGTGTGATAGAGCCATTTGCTCCTGATTACAAAACAAATGAGCCACTTGATAAAGGATTCAAAGCCATAGCTGAGGGATGCAGGGATCTTCGACGGCTCT

CCGTCTCTGGTCTTCTCTCTGACAAGGCCTTCAAATACATTGGGAAACATGCCAAGAAGGTTAGGATGCTATCAATAGCATTTGCTGGGGACAGTGATTT

GATGCTTCATCACTTGTTGTCGGGCTGTGAGAGTTTAAAGAAGCTTGAGATACGAGACTGCCCTTTTGGAGACACTGCACTACTGGAGCACGCTGCCAAG

CTAGAGACCATGCGATCCCTTTGGATGTCATCTTGCTTTGTAAGTTTTGGTGCTTGCAAGCTTCTAAGTCAGAAAATGCCAAGGCTCAATGTCGAAGTCA

TTGATGAACATCCTCCAGAGTCAAGACCTGAGAGCTCTCCAGTTGAGAGGATATACATATACAGAACAGTCGCAGGACCGAGAATGGATACGCCTGAATT

TGTGTGGACGATACACAAGAATCCTGAGAATGGAGTTTCACATCTAGCCATAAAGTAA

>AtAFB4

ATGACAGAAGAAGATAGCTCAGCTAAAATGTCAGAGGATGTTGAGAAATATCTCAACTTAAATCCACCTTGCTCCTCCTCCTCCTCTTCTTCCTCCGCCG

CTACATTCACGAACAAGTCTCGAAATTTCAAATCTTCTCCCCCGCCGTGTCCAGATCATGTCCTTGAGAACGTTTTAGAGAACGTGCTTCAGTTCCTCAC

TTCCAGATGCGATCGCAACGCAGTCTCATTGGTCTGCAGATCGTGGTATCGCGTCGAGGCTCAGACTCGATTAGAGGTTTTTATTGGAAACTGTTACTCG

CTCTCTCCTGCTCGGCTTATTCACCGGTTCAAGCGTGTTAGGTCTCTTGTGCTTAAAGGGAAACCTAGGTTTGCTGATTTTAATCTCATGCCTCCTAATT

GGGGAGCTCAATTCTCTCCTTGGGTTGCTGCTACAGCTAAGGCTTATCCTTGGCTCGAGAAGGTTCATTTGAAGCGTATGTTTGTTACGGATGATGATTT

GGCTCTTCTTGCTGAGTCGTTTCCTGGGTTCAAAGAGCTTACTTTGGTCTGCTGTGAAGGTTTTGGGACTAGTGGTATTGCTATTGTTGCTAACAAATGC

AGGCAGCTAAAGGTCCTTGATTTGATGGAGTCAGAAGTCACAGATGATGAGTTGGATTGGATTTCTTGTTTTCCTGAGGGTGAAACTCATCTGGAGTCTT

TGTCTTTTGACTGTGTTGAATCCCCTATCAATTTCAAGGCATTGGAGGAGCTCGTGGTTAGGTCACCATTCTTGAAGAAACTTAGAACGAACAGGTTTGT

TTCCCTTGAAGAGCTGCATCGACTAATGGTTCGAGCGCCGCAGTTAACGAGTCTTGGGACGGGGTCATTTAGTCCAGACAATGTGCCTCAGGGAGAACAA

CAACCGGATTATGCAGCTGCTTTTCGTGCTTGTAAATCCATAGTTTGTCTCTCAGGATTCAGGGAATTTAGACCGGAATACCTCCTAGCCATCTCTTCAG

TTTGTGCTAATCTCACCTCTCTTAACTTCAGTTATGCTAACATTTCTCCTCACATGCTCAAGCCCATCATAAGCAACTGTCACAATATCCGAGTCTTCTG

GGCTCTTGACTCGATACGTGATGAAGGACTACAGGCAGTGGCTGCCACATGCAAGGAGCTCCGTGAGCTTCGGATTTTCCCTTTTGATCCTCGTGAAGAC

AGTGAAGGTCCTGTCTCGGGAGTAGGCCTCCAAGCAATTTCAGAGGGCTGTAGGAAACTGGAATCTATCCTGTACTTTTGCCAGAATATGACCAATGGAG

CTGTGACAGCCATGTCGGAGAACTGCCCGCAGCTTACTGTGTTTAGACTTTGCATAATGGGTCGCCATAGGCCTGACCACGTGACAGGAAAGCCAATGGA

CGATGGATTTGGTGCCATTGTTAAAAACTGCAAGAAGCTAACCCGACTTGCAGTATCAGGGTTACTAACAGATGAAGCTTTTAGCTATATAGGAGAATAT

GGGAAATTGATCCGTACGCTATCTGTAGCGTTTGCTGGGAACAGTGACAAGGCTCTGAGATACGTTCTTGAGGGTTGTCCTAAACTACAAAAGCTTGAGA

TCAGGGACAGTCCCTTTGGAGATGTTGGATTGCGCTCTGGTATGCATCGGTATTCCAATATGAGGTTTGTTTGGTTGTCGTCATGTCTCATATCCCGTGG

AGGCTGCAGGGGTGTTTCTCATGCTCTGCCTAATGTAGTCGTGGAAGTATTTGGAGCCGATGGTGATGATGACGAAGACACTGTCACTGGGGATTATGTT

GAGACATTGTACTTGTATCGATCCCTTGATGGCCCAAGGAAGGATGCTCCAAAGTTTGTAACAATTTTATGA

>AtAFB5

ATGACACAAGATCGCTCAGAAATGTCTGAAGATGACGATGACCAACAATCTCCACCGTTGGATCTACCCTCTACCGCCATAGCTGATCCTTGCTCATCTT

CCTCTTCACCAAACAAATCTCGTAACTGTATCTCAAATTCTCAAACTTTCCCTGACCATGTTCTCGAAAACGTACTTGAGAACGTTCTTCAGTTCCTAGA

TTCAAGATGTGACCGTAACGCTGCTTCTCTAGTTTGCAAATCTTGGTGGCGTGTTGAAGCTTTGACTCGATCTGAGGTTTTTATTGGTAACTGTTACGCT

CTTTCTCCGGCGAGGTTGACTCAGAGATTCAAGCGTGTTAGGTCTCTTGTGCTGAAAGGGAAACCTAGGTTTGCTGATTTCAATCTCATGCCTCCTGATT

GGGGTGCTAATTTTGCTCCTTGGGTTTCTACTATGGCTCAAGCTTATCCTTGTCTTGAGAAAGTTGATTTGAAGAGGATGTTTGTTACTGATGATGATTT

AGCTCTTCTTGCTGACTCTTTTCCTGGGTTTAAAGAGCTTATCTTGGTTTGTTGTGAAGGTTTTGGTACTAGTGGTATCTCTATTGTTGCCAACAAGTGC

AGAAAGCTGAAAGTGCTTGATTTGATTGAGTCTGAGGTCACGGATGATGAAGTGGATTGGATCTCTTGTTTCCCTGAGGATGTAACTTGTTTGGAGTCTT

TAGCTTTTGACTGTGTGGAAGCTCCTATCAATTTTAAGGCGCTTGAGGGTCTTGTTGCTAGGTCACCGTTCTTGAAGAAACTTAGGCTAAACAGGTTTGT

GTCTCTTGTGGAGCTACATCGTCTGCTACTTGGAGCTCCACAGCTTACTAGTCTTGGGACTGGTTCATTTAGCCATGATGAGGAACCTCAGAGTGAGCAA

GAACCAGATTATGCTGCTGCATTTCGTGCTTGTAAATCTGTAGTTTGCTTGTCAGGGTTTAGAGAGTTGATGCCGGAGTATCTTCCAGCTATCTTTCCGG

TGTGCGCTAATCTCACCTCCCTGAACTTCAGTTATGCTAACATTTCTCCTGACATGTTCAAGCCCATCATACTCAATTGCCACAAACTCCAGGTGTTCTG

GGCCCTTGATTCAATATGTGATGAAGGACTACAGGCAGTTGCAGCCACTTGCAAGGAACTCCGTGAACTCAGGATCTTCCCTTTTGATCCTCGGGAAGAC

AGTGAAGGTCCTGTCTCTGAATTAGGCCTCCAAGCAATCTCCGAGGGTTGTAGGAAACTAGAATCTATTCTCTACTTTTGCCAGCGCATGACTAATGCCG

CTGTGATAGCCATGTCAGAGAACTGTCCAGAGCTTACTGTGTTTAGGCTGTGCATAATGGGTCGACATAGGCCTGACCATGTAACAGGAAAGCCTATGGA

CGAGGGATTTGGTGCCATTGTTAAAAACTGCAAGAAGCTAACTCGCCTTGCAGTGTCGGGATTGCTGACAGATCAAGCTTTTAGGTATATGGGTGAGTAT

GGGAAATTGGTCCGTACGCTTTCAGTAGCTTTTGCAGGGGACAGTGACATGGCTCTGAGACATGTCCTAGAAGGTTGCCCTAGACTGCAGAAACTTGAGA

TAAGGGACAGTCCCTTTGGAGATGTTGCATTACGGTCTGGTATGCATCGCTATTACAACATGAGGTTTGTTTGGATGTCAGCATGTAGCTTGTCTAAGGG

ATGCTGCAAGGATATTGCACGAGCAATGCCGAATCTAGTTGTGGAAGTAATTGGATCGGATGATGATGATGACAATAGGGATTATGTCGAGACTTTATAC

ATGTATCGGTCTCTTGATGGTCCAAGGAATGATGCACCAAAGTTCGTCACGATTTTATAG

>OsTIR1 (LOC_Os05g05800).dna (1785 bp)

ATGGGGCGCGGCGGCTCGCGCGCGGCGTGCGCCGCCGCGGCGCCGCCGTGGCACTCGCTCCCGGACGAGGTCTGGGAGCA

CGCCTTCTCCTTCCTCCCCGCCGCCGCGGACAGGGGCGCCGCGGCGGGGGCGTGCAGCTCGTGGCTCCGCGCCGAGCGCC

GGTCGCGCCGCCGCCTCGCCGTCGCCAACTGCTACGCCGCCGCGCCGCGGGACGCCGTCGAGCGGTTCCCGTCCGTGCGC

GCCGCCGAGGTCAAGGGCAAGCCCCACTTCGCCGACTTCGGCCTCGTCCCCCCCGCCTGGGGCGCCGCCGCGGCGCCGTG

GATCGCCGCCGCCGCCGACGGGTGGCCGCTGCTCGAGGAGCTCAGCTTCAAGCGCATGGTCGTCACCGACGAGTGCCTCG

AGATGATCGCCGCGTCCTTCAGGAACTTCCAGGTGCTCCGCCTCGTCTCCTGCGACGGCTTCAGCACCGCGGGCCTCGCC

GCCATTGCTGCCGGTTGCAGACACCTAAGAGAACTTGACCTGCAAGAGAACGAGATTGAGGATTGTTCTATTCATTGGCT

CAGCCTCTTCCCGGAATCGTTCACTTCTCTAGTAACTCTAAACTTTTCATGCTTAGAGGGGGAGGTCAATATCACTGTAC

TTGAACGGTTAGTGACCAGATGTCACAACCTGAAGACTCTTAAGCTCAACAATGCTATCCCCCTTGACAAGCTTGCTAGC

CTCCTTCATAAGGCTCCTCAGCTAGTTGAACTCGGAACTGGCAAATTCTCTGCTGATTACCATTCCGATCTGTTTGCAAA

GCTGGAGGCGGCGTTTGGAGGTTGTAAAAGCTTGAGAAGGCTTTCTGGGGCTTGGGATGCTGTTCCAGATTATCTGCCAG

CATTCTATTGTGTATGTGAAGGCCTCACATCACTTAATCTGAGTTATGCTACTGTGCGAGGTCCTGAGCTCATCAAATTC

ATTAGTAGATGCAGAAATTTGCAACAATTATGGGTGATGGACCTCATTGAGGATCATGGTTTAGCTGTTGTGGCATCATC

TTGCAATAAACTTCAAGAGTTGCGGGTCTTCCCTTCTGACCCTTTTGGTGCAGGATTCTTGACTGAAAGAGGTCTTGTTG

ATGTCTCTGCAAGTTGTCCAATGTTGGAGTCAGTGCTCTACTTCTGCAGACGGATGACAAATGAGGCACTTATTACCATT

GCAAAGAACCGTCCCAACTTCACTTGCTTCCGCCTATGCATCCTTGAGCCACACACTCCAGACTACATCACACGGGAGCC

TCTTGATGCAGGTTTCAGCGCCATTGTGGAGTCATGCAGGGGCCTTAGGCGTCTCTCTATCTCAGGCCTTCTCACAGATC

TTGTGTTTAAATCCATTGGGGCACATGCTGATCGTCTTGAGATGCTTTCAATCGCCTTCGCTGGGAACAGCGACTTGGGC

CTGCATTACATCCTCTCAGGCTGCAAGAGCCTGAAGAAACTGGAGATCAGGGACTGCCCATTTGGTGATAAGCCATTGCT

GGCGAACGCAGCAAAGCTGGAGACAATGCGATCCCTTTGGATGTCGTCGTGCTTGTTGACCCTGGGCGCATGCCGACAGC

TTGCACGCAAGATGCCCCGCCTTAGTGTGGAGATCATGAACGATCCTGGAAGGTCATGCCCCTTGGATTCGCTTCCGGAT

GAAACACCTGTTGAGAAACTGTACGTCTACCGGACGATCGCAGGTCCAAGGTGTAGAATGAAAATTCACAGCCTTTTTCA

GGACATGGAAATGCATGGACTCTAG

>OsAFB2 (LOC_Os04g32460).dna (1728 bp)

ATGACGTACTTCCCGGAGGAGGTGGTGGAGCACATCTTCAGCTTCCTGCCGGCGCAGCGCGACCGCAACACGGTCTCGCT

CGTCTGCAAGGTGTGGTACGAGATCGAGAGGCTGAGCCGCCGCGGCGTCTTCGTGGGCAACTGCTACGCCGTGCGCGCCG

GCCGCGTCGCCGCGCGGTTCCCCAACGTGCGGGCGCTCACGGTGAAGGGGAAGCCCCACTTCGCCGACTTCAACCTCGTG

CCCCCCGACTGGGGCGGCTACGCGGGGCCGTGGATCGAGGCGGCCGCGAGGGGATGCCACGGCCTGGAGGAGCTCAGGAT

GAAGCGGATGGTGGTGTCCGACGAGAGCCTCGAGCTGCTGGCTCGCTCGTTCCCGCGGTTCAGGGCTCTTGTTCTTATCA

GCTGCGAGGGGTTCAGCACTGACGGGCTAGCCGCCGTCGCGAGCCATTGCAAGCTTCTGAGGGAGTTGGATTTGCAGGAA

AATGAAGTGGAGGATCGAGGGCCTAGGTGGCTTTCCTGCTTCCCTGATTCCTGCACATCACTTGTCTCATTGAATTTTGC

CTGCATCAAAGGGGAGGTTAATGCTGGTTCACTGGAGAGACTTGTTAGCAGGTCCCCAAACCTGCGGAGTTTGAGGCTGA

ATCGATCTGTATCGGTAGATACACTTGCAAAGATACTACTGCGTACCCCTAACTTGGAGGATTTGGGGACAGGGAATTTG

ACAGATGACTTCCAAACTGAGTCCTACTTTAAGCTTACCAGTGCTCTGGAGAAATGCAAGATGTTGAGGAGTTTGTCTGG

ATTCTGGGATGCTTCTCCTGTTTGCCTGTCATTTATCTACCCCCTGTGTGCTCAACTGACAGGATTGAACTTGAGCTATG

CCCCCACACTTGATGCTTCTGACCTTACAAAAATGATTAGCCGCTGTGTGAAGCTCCAACGCCTTTGGGTACTGGATTGT

ATCTCGGACAAAGGCTTGCAAGTGGTGGCCTCCAGTTGCAAAGACTTGCAAGAACTCAGGGTATTTCCATCAGATTTCTA

CGTAGCTGGTTATTCTGCAGTGACAGAGGAGGGACTTGTTGCAGTATCCTTGGGCTGTCCAAAACTGAACTCACTACTGT

ACTTCTGTCACCAAATGACTAATGCTGCACTAGTTACTGTCGCCAAGAACTGTCCAAATTTCACACGATTCAGACTTTGT

ATTCTTGAGCCAGGGAAGCCTGATGTTGTGACAAGCCAACCATTAGATGAAGGCTTTGGAGCTATTGTTCGTGAGTGCAA

GGGATTACAACGTTTGTCAATATCTGGTCTTCTCACAGACAAAGTTTTCATGTATATTGGGAAATATGCAAAACAACTTG

AGATGCTTTCTATAGCATTTGCTGGTGACAGTGATAAGGGTATGATGCATGTTATGAATGGATGCAAGAATTTAAGGAAA

CTGGAGATAAGAGATAGCCCGTTTGGTGATGCTGCACTCTTGGGGAATTTTGCTAGGTACGAGACAATGCGATCCCTTTG

GATGTCATCTTGCAATGTCACGTTAAAGGGGTGCCAAGTCCTTGCGTCAAAGATGCCGATGCTCAATGTTGAGGTCATAA

ATGAGCGGGATGGTAGCAATGAAATGGAGGAAAACCATGGAGATCTGCCTAAAGTGGAGAAATTATATGTGTACCGCACA

ACTGCTGGGGCGAGGGATGATGCACCAAATTTTGTTAAAATCCTATAG

>OsAFB3 (LOC_Os11g31620).dna (1707 bp)

atggtgttcttcccggaggaggtggtggagcacatccttgggtttctagcgtcgcaccgcgaccgcaacgcggtgtcgct

ggtgtgccgggagtggtaccgcgtcgagcgcctcagccgccgctcggtgctcgtgcgcaactgctacgcggcgcgcccgg

agcgcgtgcacgcgcgcttccccggcctgcgctcgctgagcgtgaaggggaggccgcgctttgtccccgcggggtggggt

gccgcggcgcggccatgggtggccgcgtgcgtcgccgcgtgccctggcctcgaggagctccggctgaagcggatggttgt

cactgatgggtgcctcaagctgctcgcttgctcattccccaatttgaagtcgctcgtccttgttggctgtcaggggttca

gcactgatgggcttgctactgtcgctaccaattgcagatttatgaaggaactggacttacaagagagtctggtggaggat

cgagactctcgttggcttggatgttttcccaagccttctacattactagaatccttgaatttttcttgcttgaccgggga

ggtgaatagtcctgcattggaaatactggttgcaaggagtccaaatcttagaagcttgagattgaaccgttcagttccac

ttgatgttttggccagaattctttgtcgcagacctaggctggtggatttatgtacaggatcttttgtacgaggcaatatt

gttggtgcatatgctgggctattcaatagttttcaacattgcagtttgctgaagagtttatctgggttttgggatgctac

aagcttgtttattccagtgattgctcctgtttgcaagaatctaacgtgcttgaaccttagctctgctccaatggtcagga

gtgcttatcttattgaatttatttgtcaatgcaagaaactccaacaattgtgggtgttagatcacattggtgatgaagga

ttgaaaattgtagcctcatcctgtatacagctccaagagttgagagtatttcctgcgaatgcaaatgccagagcaagcac

tgtgacagaggaagggctagttgccatatctgcaggctgtaacaagttacagtctgtgctctatttttgccaacggatga

caaactctgcactgattactgtcgcaaagaactgcccacgattcacgtccttcagactgtgtgttcttgatccaggatca

gcagacgctgtgacagggcagccattggatgagggttatggggcaatcgtacagtcatgcaaaggccttagacggctatg

tttgtctggacttctcacagacacagtgttcctctacattggcatgtatgctgagaggctggagatgctttctgtagcat

ttgcaggagataccgacgatggcatgacctacgtgctcaatggctgcaaaaatctcaagaagctggaaatcagggacagt

ccttttggtgacagcgcgcttcttgcaggcatgcatcagtacgaggcaatgcgctcactctggttgtcgtcgtgtaatgt

caccctggggggttgcaagtctcttgcagcaagcatggcaaacctcaacatcgaggtcatgaatagagcagcaagtatca

atgaggcagacaatgctaatgatgcaaagaaggtgaagaagttgtatatttacaggacagttgctggaccgaggggtgat

gcgcctgaattcatctcaacgttctaa

>OsAFB4 (LOC_Os02g52230).dna (2004 bp)

ATGAGCACTTCCCCCTCCTGCTCCTCCTCCTCGCCGATCCCCCAATCCCTAACCCTAGCCTCCACCTCCTCCTCCTCCTC

ATCCTCCGGGATGCGCGATGCGGGGGAGGGGTCGGACTCGCCGCCGTCGGAGATGTCGGAGGATGGGTCAGGAGGGAGCG

GGGACGGGGACGGGGACGGGGACGGGGGAGGGGGAGGCGGGGACAGGTGGATGCCGGATCTGAGGGGAGGGAACGGCGGC

GGCGGCGGAGGAGGCGGAGGGGGAGGGAGGTGGGCGCCGCCGGACCAGGTGCTGGAGAACGTGCTGGAGAGCGTGCTGGA

GTTCCTGACGGCGGCGCGGGACCGGAACGCGGCGTCGCTGGTGTGCCGGTCGTGGTACCGCGCCGAGGCGCAGACGCGGC

GGGAGCTGTTCATCGGCAACTGCTACGCGGTGTCGCCGCGCCGCGCCGTGGAGCGGTTCGGAGGGGTGCGCGCCGTGGTG

CTCAAGGGGAAGCCGCGGTTCGCGGACTTCAGCCTCGTGCCCTACGGCTGGGGCGCCTACGTCTCCCCCTGGGTCGCCGC

GCTCGGCCCCGCCTACCCGCACCTCGAGCGCATCTGCCTCAAGCGCATGACCGTCTCCAACGACGACCTCGCGCTCATCG

CCAAGTCATTCCCGCTGTTCAAGGAGCTGTCGCTGGTGTGCTGCGATGGGTTCAGCACGCTAGGCCTCGCCGCCATCGCC

GAGCGGTGCCGGCATCTCCGTGTGCTGGATCTGATTGAAGACTATATTGACGAGGAGGAGGATGAGCTAGTGGATTGGAT

CTCCAAGTTCCCGGAGTCCAACACGTCGCTGGAGTCACTTGTGTTTGATTGTGTTAGTGTCCCATTCAACTTTGAGGCCC

TGGAGGCGCTTGTTGCACGCTCACCAGCTATGCGCCGGTTGCGAATGAATCACCATGTGACAGTAGAGCAATTGCGCCGT

CTAATGGCAAGGGCTCCCCAGCTCACACACCTTGGTACTGGTGCATTCCGTTCTGAGCCAGGCCCTGGTGGTGCTCTGTC

TGTTACTGAGCTTGCTACATCTTTTGCGGCATCTAGGTCTCTGATTTGTTTGTCAGGTTTCCGGGATGTCAATCCAGAAT

ACCTCCCAGCAATCCACCCAGTCTGCGCTAATCTCACTTCCCTTAATTTTAGCTTTGCAAACCTAACTGCTGAGGAGCTC

ACACCGATTATTCGCAACTGCGTCCGTCTTCGCACTTTCTGGGTTCTAGATACAGTGGGTGATGAAGGCCTTCGGGCTGT

GGCTGAGACATGCTCAGATCTTCGTGAGCTGCGAGTTTTTCCTTTCGATGCCACTGAGGATTCTGAGGGATCGGTTTCAG

ATGTTGGTCTTCAGGCAATCTCGGAAGGGTGCCGGAAGCTTGAATCAATTCTCTACTTTTGCCAGCGCATGACAAATGCA

GCAGTAATTGCTATGTCCAAGAACTGTTCTGACCTGGTAACATTCCGTCTTTGTATTATGGGGCGACACCGCCCTGATCG

GATCACTGGGGAGCCCATGGATGATGGTTTTGGGGCAATTGTGATGAACTGCAAGAAGCTCACTAGACTTTCAGTCTCTG

GTCTGCTCACTGATAAGGCGTTTGCATACATTGGAAAATATGGGAAACTAATAAAGACACTGTCTGTTGCCTTCGCTGGA

AATAGTGACATGTCTCTCCAATCTGTGTTTGAAGGATGCACTAGGTTGCAAAAGCTTGAGGTCAGAGATAGTCCTTTTAG

TGATAAGGGATTGCTCTCTGGCCTGAGCTATTTTTACAACATGAGGTTCTTATGGATGAATTCATGCAGGCTAACCATGA

GGGGTTGTAGAGATGTAGCTCAGCAAATGCCTGACTTGGTGGTTGAAGTGATGAAGGATCATCTTGATGATGAAGGGGAG

ATGGAGACTGTTGATAAACTGTACTTGTATCGATCACTGGCAGGAGCAAGGAATGATGCACCTTCATTTGTCAACATCTT

GTAG

>OsAFB5 (LOC_Os03g08850).dna (1812 bp)

ATGTCCGAGGAGGACGACGACCAGCCGCCGCCGCTGCCGGCGCAGAAGCGGCCGCGCGCGTCGCCGCCGCCGGACCAGGT

GCTCGACAACGTCCTCGAGACGGTGCTCCAGTTCCTCGACTCGGCGCGGGACCGGTGCGCGGCGTCGCTGGTGTGCCGCT

CGTGGAGCCGGGCCGAGTCCGCCACCCGCGCCTCCGTCGCCGTCCGCAACCTCCTCGCCGCGTCCCCGGCGCGCGTCGCG

CGACGCTTCCCGGCCGCGCGGCGCGTCCTCCTCAAGGGCCGCCCGCGCTTCGCCGACTTCAACCTCCTCCCGCCAGGCTG

GGCCGGCGCCGACTTCCGCCCCTGGGCAGCCGCCGTCGCCGCCGCCGCGTTCCCCGCGCTCGCCTCCCTCTTCCTCAAGC

GCATCACCGTCACCGACGACGACCTGGACCTCGTCTCCCGCTCCCTCCCCGCCTCCTTCCGCGACCTCTCGCTCCTCCTC

TGCGACGGCTTCTCCTCCGCTGGCCTCGCATCCATCGCTTCCCATTGCAGGGGGCTGCGAGTGCTCGATGTGGTTGACTG

CGAGATGAACGACGACGACGACGAGGTGGTGGACTGGGTGGCGGCGTTCCCGCCGGGGACGACCGACCTCGAATCGCTCT

CCTTCGAGTGCTACGTCCGGCCGGTGTCCTTCGCCGCGCTCGAGGCGCTCGTGGCGCGCTCGCCGCGCCTCACCCGCCTG

GGCGTCAACGAGCACGTGTCGCTGGGGCAGCTGCGCCGGCTCATGGCGAACACGCCTCGCCTGACGCACCTCGGCACCGG

AGCGTTCCGGCCGGGGGACGGCCCCGAGGATGTGGGGCTCGACATCGAGCAGATGGCGTCCGCGTTCGCGTCCGCTGGCC

GGACGAACACGCTGGTTTCGCTGTCTGGCTTCCGCGAGTTCGAGCCGGAGTACCTGCCCACCATTGCCGCCGTGTCCGGC

AACCTAACGAACCTCGACTTCAGCTATTGCCCGGTCACTCCCGATCAATTCCTGCCCTTCATCGGGCAATGCCACAACCT

TGAGAGACTATATGTGCTTGATTCGGTGCGTGACGAGGGGCTCCAGGCCACGGCGAGGACTTGCAAGAAGCTCCAGGTTC

TCCATGTGCTTCCATTGAACGCACTTGAGGATGCCGATGAGCTGGTGTCGGAGGTCGGGCTTACTGCCATTGCTGAGGGC

TGCCGAGGGCTCCGTTCGACGCTTTACTTCTGCCAGAGTATGACCAACGCTGCGGTGATCGCCATTTCTCAAAATTGCGT

GGACCTTAAGGTATTCCGGTTATGCATAATGGGACGTCACCAGCCTGACCATGTGACTGGGGAGCCCATGGATGAAGGGT

TTGGTGCCATTGTTAGGAACTGCAGCAAGCTTACTAGGCTCTCCACATCTGGACACCTGACTGATCGAGCTTTCGAGTAC

ATTGGCAAGTATGCCAAGTCGCTCCGGACGCTCTCTGTTGCGTTCGCTGGAGACAGCAATCTGGCGTTGCAACACATCCT

CCAGGGGTGCTCGAAGCTGGAGAAGCTGGAGATAAGGGATTGCCCATTTGGGGATGCTGGCCTCCTCTCCGGAATGCACC

ATTTCTATAACATGCGGTTCCTCTGGATGTCAGGTTGCAACCTTACGCTGCAAGGTTGCAAGGAGGTCGCACGGAGGCTA

CCAAGATTGGTGGTGGAGCTGATAAATAGCCAGCCTGAGAACGAAAGGACCGACAGCGTGGACATCTTATACATGTATCG

GTCGCTTGAAGGGCCAAGAGAGGATGTACCACCATTCGTGAAGATCCTATAA

>PtrFBL1 (Potri.014G134800)

ATGTTGAGAAAGGCGAATTCGTTCCCGGAGGAAGTGCTACAGCATGTGTTGTCTTTTATCACAAACGACAAAGACAGGAACGCCGTATCGCTGGTGTGTAAGTCGTGGTACGAGATAGAGAGGTGGTGTAGGAAGAGGATATTTGTAGGTAACTGTTATGCGGTGAGGCCGGAGATGGTTATAAGGAGGTTTCCGGAGCTCAGATCGGTGGAGCTCAAAGGAAAGCCGCACTTTGCGGACTTTAGTTTGGTGCCTGACGGTTGGGGAGGTTGTGTTTACCCTTGGATCGCGGCGTTGGCTACTGCGTATCCGTGGTTGGAAGAGATTAGTTTGGAGAGGATGGTGGTTTCAGATGAGAGTTTGAAGGTGATTGCTAAGAGTTTTAAGAATTTTAAGGTTTTGGTGCTTTCGTCTTGTGAGGGTTTCTCTACTGATGGACTTGCTGCCGTTGCGGCCAATTGCAGGAATCTGAGGGGGCTGGATTTGCGAGAGAGTGAGGTGGATGATCCAAGTGGGCAGTGGTTGAGCCGCTTTCCTGACTCGTTTACATCACTCGCGTCTCTTAATATTTCCTGCTTGGGGGCTGAGGTTAGTTTCTCAGCTCTGGAGCGCCTGGTAGGCCGGTGTCCTGATCTGAAGACTCTTCGGCTCAACCATGCTGTGCCCCTTGACAAGCTTGCCAATCTTCTTCGCGGTGCACCACAACTGGTTGAATTGGGCACAGGTGCTTACTCAGCCGAGTTGCAGCCTGATGTATTCTCAAACCTGGCAGGAGCTTTTTCTGGGTGTAAAGAACTGAGGAGCCTATCTGGGTTTTGGAATGTTTTTCCAGGTTACCTTCCAGCAGTTTATCCTGTTTGTTCTGGCTTAACATCACTCAACTTGAGGTATGCTAATATACAAGGCGCTGATCTCATTAAGCTTGTCAGTCAATGCCCAAGTTTGCAGCGCTTATGGGTGCTGGATTACATTGAAGACATTGGACTCGAAGCTCTTGCAGCATGTTGCAAGGACCTGACAGAGTTGAGGGTGTTTCCATCTGACCCATATGGTGCGGAACCAAATGTATCCTTGACAGAAAGGGGCCTTGTCTCTGTCTCTGAAGGCTGTCCCAAGCTTCATTCAGTTCTGTACTTTTGCCGTCAAATGACTAATGCTGCCCTAGTTACAATAGCAAAGAACCGTCCAAGTATGACTTGCTTTCGTCTTTGTATTATTGAACCACGGGCTCCTGATTATCAAACACTCCAGCCTCTGGATTTGGGTTTTGGAGCCATTGTTGAAAACTACAAGGATCTCCGGCGTCTTTCCCTCTCAGGTCTATTGACTGATCGCGTGTTTGAGTACATTGGAACTTATGCCAAGAAGTTAGAGATGCTATCTGTGGCATTTGCTGGGGATAGTGATCTGGGACTCCACCATGTGTTGTCTGGGTGCGAAAAGCTTTGCAAACTAGAGATAAGGGATTGTCCCTTTGGTGACAAAGCTCTTTTGGCCAATGCTGCAAAGCTGGAGACAATGCGATCCCTTTGGATGTCTTCTTGCTCTGTGAGTTTCAGAGCATGTAAGCTGCTGGGTCAGAAGATGCCTAGACTCAATGTTGAAGTTATTGATGAGAGGGGACCTCCAGATTTGAGGCCGGAAAGCTGCCCTGTTGAGAAGCTTTATATATACAGAACCATTGCGGGGCCTAGGTTTGACATGCCTGGATTTGTTTGGACAATGGATGAAGATTCTGTGTCAAGGTTTTCTTGA

>PtrFBL2 (Potri.002G207800)

ATGCCGAACAAGGCGAGTACGTTCCCGGAGGAAGTGCTAGAGCATGTGCTCTCGTTTATTACAAACGACAAAGACAGGAACGCCGTATCGGTGGTGTGCAAGTCGTGGTACGAGATAGAGCGGTGGTGCAGGAAGAGAATATTTGTTGGTAACTGTTATGCGGTGAGGCCTGATATGGTTATAAGGAGGTTTCCGGAGCTAAGATCGGTGGAGCTCAAAGGGAAGCCACACTTTGCGGACTTTAATTTGGTGCCTGACGGTTGGGGAGGTTACTTTTACCCATGGATAGCGGCGTTGGCTACCGCCTATCCTTGGTTGGAAGAGATTAGGTTGAAGAGGATGGTGATTTCCGACGAGAGTTTGGAGTTTATTGCTAAGAGTTTTAAAAATTTTAAGGTTTTGGTGCTTTCGTCTTGTGAGGGTTTCTCTACGGATGGACTTTCTGCCATTGCAGCCGATTGCAGGAATCTGAGGGAGCTGGATTTACGAGAGAGTGAGGTGGATGATCCAAGTGGGCAGTGGTTGAACAGTTTTCCTGACTCGTTTACATCACTGGTCTCTCTTAATATTTCCTGCTTAGGGGCTGAGCTGAGTTTCTCAGCTCTGGAGCGCCTGGTTGGCCAGTGTCCTGATCTAAAGAATCTTCAGCTCAACCATGCTGTGCCCGTTGAGAGGCTTGCTAAACTTATTCGCCAAGCACCACAACTTGTTGAATTGGGCACAGGTGAGTTCTCGGCTAAGTTGCAGCCTGAAATCTTCTCAAACCTGGCTGGAGCTTTTTCTGTGTGTAAAGAACTGAGGAGTCTATCTGGGTTTTGGGATGTAAATCCAGCTTACCTTCCAGCAGTTTATCCTGTCTGTTCTGGCTTAACATCACTGAACTTGAGATATGCCAATATACAAAGCGCTGATCTCATTAAACTTGTCAGTCAATGTTCGAATCTGCAGCGTTTATGGGTGCTGGATTACATTGAAGACGTTGGACTTGAAGCTCTTGCAGCATGTTGCAAGGACCTGACAGAGTTGAGGGTGTTTCCATCTGACCCATTTGCTGCAGAACCAAATGTATCCTTGACAGAAAGGGGCCTTGTCTCTGTCTCTGAAGGCTGTCCTAAGCTTCAGTCAGTTCTGTACTTTTGCCGTCAAATGACTAATGCTGCTCTAGTTACAGTAGCAAAGAACCGTCCAAGCATGACCTGCTTCCGTCTTTGTATTATTGAACCTCAGGCTCCTGATTACCAAACACTTCAGCCTCTGGATTTGGGTTTTGGAGCCATTGTTGAAAACTACAAGGATCTCCGGCGTCTTTCCCTCTCAGGTCTACTGACTGATCGTGTGTTTGAGTACATTGGAACTTATGGAAAAAAAATAGAGATGCTATCTGTGGCATTTGCTGGCGATAGTGATCTGGGACTCCACCATGTGCTGTCTGGGTGTGAAAGGCTTTGCAAACTTGAAATTAGGGACTGTTCCTTTGGGGATAAGGCTCTTTTGGCCAATGCTGCAAAGCTGGAGACAATGCGATCCCTTTGGATGTCTTCTTGCTCAGTGAGTTTTGGAGCATGTAAGCTGCTAGGTCAGAAGATGCCTAGGCTCAATGTTGAAGTTATTGATGAGAGGGGACCTCCAGAATCGAGGCCGGAAAGCTGCCCTGTTGAGAAGCTTTACATATACAGAACCATTGCAGGACCTAGGCTTGACATGCCTGGATTTGTTAGGACTATGGATGCAGATTCTGTGTCAAGGTTTTGTTGA

>PtrFBL3 (Potri.001G323100)

ATGAATTATTTCCCTGATGAAGTATTAGAGCATATTTTCGATTTTGTAACATCACAAAGAGACAGGAACTCAGTGTCTCAAGTGTGTAAACCATGGTACAAAATCGAAAGTACTAGCAGGCAAAAGGTTTTTGTAGGGAATTGTTATGCAATTAGTCCTGAGAGAGTGATTGAGAGGTTTCCAGGTTTGAAATCTATCACTTTGAAAGGAAAGCCTCATTTTGCTGATTTTAATTTGGTTCCTCATGATTGGGGAGGCTTTGTTTATCCATGGATTGAAGCTTTTGCAAGGAATAATATGGGGTTAGAGGAGCTCAAGTTGAAGAGGATGATAATATCCGATGAGTGCTTGGAGCTGATTTCAAGGTCTTTTGCCAATTTCAAGTCCTTGGTTCTTGTTAGTTGTGAAGGCTTCAGCACTGATGGCCTTGCTGCTATTGCTTCTAATTGTAGGTTTCTGAGGGAGCTGGACCTGCAAGAAAATGATGTCGAGGATCATAGAGGCCATTGGCTTAGCTTCTTTCCTGACACTTGTACATCTCTTGTATCACTTAATTTTGCATGTCTCAAAGGAGATGTCAATTTAGCAGCTCTTGAGAGACTTGTAGCTAGATCTCCTAATCTGAGGAGTTTGAGGTTAAATCATGCCGTGCCACTTGATATACTTCAAAAAATATTGATGAGAGCACCTCATTTAGTGGACTTGGGTGTAGGGTCTTACGTGCATGATCCAGATTCTGAGACCTATAATAAATTAGTGACTGCTCTTCAAAAGTGTAAGTCAGTCAAGAGTTTGTCAGGGTTTCTGGAGGCTGCTCCTCAATGCCTATCGGCTTTTCATTTAATTTGCCCGAACCTGACTTCCTTGAACCTAAGCTATGCTCCAGGAATTCATGGTACTGAGCTCATAAAGCTAATTCGTCACTGCAGGAAACTCCAGCGCTTATGGATACTGGACTGCATTGGAGATGAAGGACTAGAAGTTGTAGCTTCCACTTGCAAACATTTGCAGGAAATAAGGGTCTTTCCTTCTGATCCATTTGTTGGGAATGCAGCTGTGACTGAAGTGGGCTTGGTTGCTCTTTCAAGTGGTTGCCGCAACCTTCACTCAATCCTATACTTCTGTCAGCAGATGACCAATGCAGCCCTCATAACTGTAGCTAAGAACTGCCCCAATTTTACCCGTTTCAGGTTGTGCATCCTTGACCCCACAAAACCGGACGCTGATACCAATCAGCCATTGGATGAAGGTTTTGGGGCTATTGTTCACTCATGCAAGGGGCTCAGGCGGTTGTCAATGTCTGGTCTGCTGACTGATCAAGTTTTCCTCTACATTGGAATGTATGCCGAGCAGCTTGAAATGCTTTCTATTGCATTTGCTGGGGACACTGACAAGGGAATGCAGTATCTATTGAATGGTTGCAAGAAACTTCGCAAGCTTGAGATAAGGGACTGCCCTTTTGGTAATGCAGCACTTTTAATGGACGTGGGAAAGTATGAAACAATGCGATCCCTTTGGATGTCATCCTGCGACATTACCCTTGGAGGCTGCAAGTCCCTTGCGAAGAAGATGCCAAGGCTCAATGTGGAGATCATAAATGAAAGTGACCAGATGGATATTACCGCTGATGATGGGCAAAAGGTAGAGAAGATGTTCTTGTATCGGACTTTGGCAGGGCGAAGGAAAGATGCACCAGAGTTCGTGTGGACTTTATAG

>PtrFBL4 (Potri.017G061600)

ATGAATTATTTCCCAGATGAAGTATTAGAGCATATTTTCGATTTTGTAACATCACAAAGAGACAGGAACTCAGTTTCTCAAGTATGTAAACCATGGTACAAGATCGAAAGTAGTAGCAGGCAGAAGGTTTTTGTAGGGAATTGTTATGCAATTAGTCCTCAGAGAGTGATTGAGAGGTTTCCTGGTTTAAAATCTATTACTTTGAAAGGAAAGCCTCATTTTGCTGATTTTAATTTGGTTCCAAATGATTGGGGAGGCTTTGTTTATCCATGGATTGAAGCTTTTGCAAGGAATAGTGTGGGGTTAGAGGAGCTCAAGTTGAAGAGGATGATAATATCCGATGAGTGCTTGGAGCTCATTTCAAGGTCTTTTCCCAATTTCAAGTCCTTGGTTCTTGTTAGTTGTGAAGGCTTCACTGCTGATGGCCTTGCTGCTATTGCTTCTAATTGTAGGTTTCTTAGGGAGCTGGACCTGCAAGAAAACGATGTTGAAGATCATAGAGGCCATTGGCTTAGCTGCTTTCCTGATACCTGTACATCTCTTGTATCACTTAATTTTGCATGTCTCAAAGGTGAGGTCAATGTAGCAGCACTTGAGAGACTTATAGCAAGATCTCCTAATCTGAGGAGTTTGAGGTTAAATCATGCAGTGCCACTTGATGTACTTCAAAAAATATTGATCCGAGCACCTCATTTAGTGGACTTGGGTGTAGGGTCTTATGTAAATGATCCAGATTCTGAGACCTATAATAAATTAGTGATGGCTATTCAAAAGTGTATGTCAGTCAAGAGTTTGTCAGGATTTCTGGAGGTTGCTCCCCACTGCCTATCAGCTTTTCACTTAATTTGCCCGAACTTGACTTCCTTGAACTTAAGTTATGCTCCAGGAATTCATGGTGCTGAGCTCATAAAGCTAATTCGCCACTGCATGAAACTCCAGCGCTTATGGATACTGGACTGCATTGGAGACCAAGGACTAGAAGTTGTTGCTTCCACTTGCAAGGATTTGCAGGAAATAAGGGTCTTTCCTTCTGATCCACACGTTGGGAATGCAGCTGTGACAGAAGTGGGCTTGGTCGCTCTTTCTAGTGGTTGCCGTAAGCTCCACTCAATTCTATACTTCTGTCAGCAGATGACCAATGTAGCCCTCATTACTGTAGCTAAGAACTGCCCCAACTTTACCCGCTTCCGGTTGTGCATCCTTGACCCCACAAAACCAGACGCTGTGACCAATCAGCCATTGGATGAAGGTTTTGGGGCTATTGTTCATTCATGCAAGGGTCTCAGGCGGTTGTCAATGACTGGCCTGCTGACTGATAAAGTTTTCCTCTACATTGGAATGTATGCCGAGCAGCTTGAAATGCTTTCTATTGCATTTGCTGGGGACACTGACAAGGGAATGCAGTATCTGTTGAATGGTTGCAAAAAGCTTCGCAAGCTAGAGATAAGGGACTGCCCTTTTGGTAATGCAGCGCTTTTAATGGACGTGGGAAAGTATGAAACAATGCGATCCCTTTGGATGTCATCGTGCGAAGTTACCCTTGGAGGCTGCAAGTCCCTTGCGAAGAAGATGCCAAGGCTCAATGTGGAGATCATTAATGAAAATGATCAGATGGATGCTAGTGCTGATGATAGGCAAAAGGTAGAGAAGATGTTCCTATATCGGACTTTGGCGGGGCGAAGGGAAGATGCACCAGAGTTCGTTTGGACTTTATAG

>PtrFBL5 (Potri.004G033900)

ATGGGTCCAAATCCAAAGATGAAAAGGGAATTTCTTGACTCAACAAGGTCATCACCATTCCCAGATGAAGTACTGGAACGAGTTCTTTCACTCTTGAAATCACACAAAGATCGTAGCGCAGTGTCTTTAGTATGCAAGGACTGGTACAACGCAGAAAGCTGGTCAAGAACTCATGTTTTTATAGGGAACTGTTATTCAGTCTCTCCTGAAATTGTTGCACGTAGATTTCCAATAATTAAGAGTGTTACTCTTAAAGGGAAGCCAAGATTTTCTGATTTTAATCTAGTGCCTGAAAATTGGGGAGCTGATGTTCATCCTTGGCTTGTAGTTTTTGCAACGAAGTATCCGTTTTTGGAAGAGCTGAGGCTTAAGAGAATGGCTGTTAGTGATGAAAGTTTGGAGTTTTTGGCTGTTAATTTTCCTAATTTTAAGGTTCTTTCTCTGTTGAGCTGTGATGGGTTTAGTACTGATGGGCTTGCTGCTATTGCCACTCATTGCAAGAGTTTGACTCAGCTTGACATACAAGAGAATGGCATTGATGACAAGAGTGGTGGTTGGTTAAGCTGCTTCCCTGAAAACTTCACATCATTGGAAGTACTAAACTTTGCCAATCTGAATACTGATGTCAATTTCGATGCACTTGAGAGACTTGTAAGTAGGTGCAAGTCACTAAAGGTTCTGAAGGTCAACAAAAGTATTTCCTTGGAACACCTACAAAGGCTGTTAGTTTGTGCTCCACAATTAACAGAGCTTGGCACTGGTTCATTCACACCAGAGCTCACAACTCGTCAGTATGCGGAGCTTGAAAGTGCATTTAACCAGTGCAAGAATTTACATACCCTTTCAGGTTTATGGGAGGCAACAGCACTATATCTACCAGTTCTGTACCCTGTCTGTTCAAACTTGACTTTCTTAAACCTGAGCTATACTTTTCTGCAAAGTCTTGAACTTGCTAGTCTTCTCCGTCAATGTCCACGACTTAGACGCCTCTGGGTCCTGGACACTGTGGGGGACAAAGGGCTGGAGGCTGTTGGATCCAACTGTCCATTGCTTGAGGAGCTCCGTGTCTTCCCTGCCGATCCCTTTGATGAGGAAATTATCCACGGGGTGACTGAAGCAGGGTTTGTTGCTGTCTCTTATGGATGTCGAAGACTCCACTATGTTCTCTACTTTTGCCGGCAGATGACTAATGCTGCAGTAGCAACCATTGTGCAGAACTGCCCTGATTTCACCCACTTTCGTCTTTGCATAATGAATCCAGGCCAACCGGATTACTTGACAAATGAACCTATGGACGAGGCTTTTGGGGCTGTGGTGAGGACTTGCACTAAACTACAGAGGCTTTCTGTTTCAGGTCTCTTGACAGACCTGACATTTGAATATATTGGGCAGTATGCCAAAAATCTGGAAACTCTGTCTGTGGCTTTTGCGGGCAGCAGTGATAGGGGGATGCAGTGTGTGCTAGAAGGTTGTCCAAAGTTGAGGAAACTTGAGATAAGGGACTGCCCATTTGGAAATGCAGCACTTCTTTCAGGTTTGGAGAAGTACGAGTCTATGAGGTCACTCTGGATGTCAGCCTGCAATGTGACAATGAATGGCTGTCGGTTATTGGCAAGGGAGATGCCCAGATTGAATGTTGAAGTAATGAAAGAGGATGGAAGTGATGACTCTCAGGCTGATAAAGTTTATGTTTACCGTTCTGTTGCGGGGCCAAGAAGGGATGCTCCACCTTGTGTACTCACTCTCTCAGGTTTATAA

>PtrFBL6 (Potri.011G042400)

ATGGATTCGAATCCAAAGATGAGAAAGGAATTTCTTGACTCAACAAGATCATCTCTATTTCCAGATGAAGTACTGGAACGAGTTCTTTCACTCTTGAAATCACACAAAGATCGTAGCGCAGTCTCTTTAGTATGCAAGGATTGGTACAACGCAGAAAGCTGGTCAAGAACTCATGTTTTTATAGGAAACTGTTATTCAGTCTCTCCTGAAATTGTCGCACGTAGATTCCCAAGAATTAAGAGTGTGACTCTCAAAGGGAAGCCGAGATTTTCTGATTTTAATCTGGTCCCTGAAAACTGGGGAGCTGATGTTCATCCTTGGTTTGTAGTTTTTGCTGCAAAGTATCCCTTTTTGGAAGAGTTAAGGCTTAAGAGAATGGCTGTTAGCGATGAAAGTTTGGAGTTTTTGGCTCTTAATTTTCCTAATTTCAAGGTTCTTTCTCTTTTGAGCTGTGATGGGTTTAGTACTGATGGGCTTGCTGCTATTGCCACTCATTGCAAGAATTTGACTCAGCTTGATATACAAGAGAATGGCATTGATGACAAGAGTGGCAACTGGTTAAGCTGCTTCCCTGAAAACTTCACATCATTGGAAGTACTAAACTTTGCCAACTTGAACACTGATGTCAATTTTGATGCACTTGAGAGGCTTGTAAGTCGGTGCAAATCACTGAAGGTTCTGAAGGCCAACAAAAGTATTTCCTTGGAACAACTACAAAGGCTGCTGGTTTGTGCTCCACAGTTAACAGAGCTTGGTACTGGTTCATTCATGCCAGAGCTTACAGCTCGCCAGTACGCTGAGCTTGGAAGTTCATTTAACCAGCTGAAGAATTTAAATACCCTTTCTGGTTTATGGGAGGCAACAGCACCATACTTACCAGTTCTCTATCCTGCCTGTACAAACTTGACTTTCTTAAACTTGAGCTATGCCTTTCTGCAAAGTATTGAACTTGCGAGTCTTCTTTGTCAATGTCCACGTCTTCGACGCCTCTGGGTCTTGGACACTGTGGGGGACAAAGGTTTAGAGGCTGTTGGATCTAACTGTCCATTGCTTGAGGAGCTCCGTGTCTTCCCTGCAGATCCCTTTGATGAGGAAGTTATCCATGGGGTGACTGAAGCTGGGTTTCTTGCTGTGTCTTACGGATGTCGGAGACTCCACTATGTTCTCTACTTTTGCAGGCAGATGACTAATGCTGCAGTAGCCACCATTGTGCAGAACTGCCCAGATTTCACCCACTTCCGTCTCTGCATAATGAATCCAGGCCAACCAGATTACTTGACAAACGAACCCATGGATGAAGCTTTTGGGGCGGTAGTGAGGACATGCACTAAACTACAGAGGCTTTCTGTTTCAGGTCTCTTGACTGACCTGACATTCGAGTATATTGGGCAGTATGCCAAAAATCTGGAAACTCTGTCAGTGGCTTTTGCTGGCAGTAGTGATAGGGGGATGCAGTGCATGCTGGAAGGTTGTCCAAAGTTGAGGAAACTTGAGATAAGGGACTGCCCATTTGGAAATGCAGCACTTCTTTCAGGTTTGGAGAAATACGAGTCAATGAGGTCACTTTGGATGTCAGCCTGCAATGTGACAATGAATGGCTGTCGGGTATTGGCAAGGGAGATGCCCAGATTGAATGTAGAGGTAATGAAAGAGGATGGGAGTGATGATTCTCAGGCTGATAAAGTTTATGTTTATCGTTCTGTTGTGGGACCAAGAAGAGATGCCCCGCCTTGTGTACTGACTCTCTCAGGTTTATAA

>PtrFBL7 (Potri.005G159300)

ATGATCACCAACAAAAAGCCTAGATCATCAGATACTGACTCTAATTACATGAGAGACGATCGAACTGAAATGTCCGAAGACGACGACAGATCTCCTCCCTCCAACTCAATCACCCATGATTCTAGCCCAACCCGGACCTGCACCCCCGGGCCCGGGTCGGGTTCATCTTCAGTCCCCGAATACTTAGCTCCGTACCCGGACCAAGTCCTTGAAAACGTCTTAGAAAACGTTCTCTGGTTCTTGACCTCACGTAAGGACCGAAACGCTGCGTCATTGGTTTGTAGGTCATGGTACCGGGTCGAGGCTCTGACCCGATCCGATTTGTTCATCGGTAACTGCTACGCGGTGTCTCCAAAGCGAGCCATGTCGCGGTTTACCCGAATCAGGTCGGTGACGCTGAAAGGGAAGCCAAGGTTTGCTGATTTTAACCTGATGCCGCCTAATTGGGGAGCCCACTTCGCGCCTTGGGTCTCTGCTATGGCAATGACTTACCCTTGGTTAGAGAAGGTTCATTTGAAGAGGATGTCAGTGACGGATGATGATCTGGCTTTGCTTGCGGAGTCGTTTTCGGGATTCAAAGAGCTCGTGCTTGTTTGTTGTGATGGGTTTGGTACTAGTGGACTTGCTATTGTCGTTAGCAGGTGCAGGCAACTCAAAGTGCTTGATCTGATTGAATCAGAGGTATCAGATGATGAAGTGGATTGGATTTCGTGTTTTCCAGATACCGAAACATGCCTCGAATCCCTGATTTTTGATTGTGTAGATTGTCCCATTGATTTTGATGCACTGGAGAGGCTGGTGGCTAGGTCCCCATCACTTAAGAAACTTAGGCTGAACCGATATGTTTCGATTGGACAACTTTACCGTCTAATGGTTCGAGCTCCACACCTCACACATCTTGGGACAGGCTCTTTTAGCCCATCAGAGGATGTAGCTCAGGTTGAACAGGGACCGGATTATGCTTCTGCGTTTGCTGCTTGCAAATCCTTAGTCTGCCTATCTGGATTCAGGGAACTCATTCCAGATTACTTGCCTGCAATAAACCCTGTATGTGCCAATCTCACTTCACTGAACTTTAGTTATGCAGAAGTTAGTGCAGAACAGCTCAAACCAATCATAAGCAATTGCCACAAGCTTCAGATTTTCTGGGTTCTTGATTCAATATGCGATGAAGGACTACAGGCTGTGGCTGCAACATGCAAGGAGCTACGAGAGCTTCGGGTCTTCCCTGTTGACCCTAGGGAGGACATTGAGGGCCCTGTTTCTGAAGTGGGCCTCCAAGCAATTTCAGAGGGTTGCAGGAAGCTTCAATCTATTTTGTATTTTTGCCATCGGATGACAAATGCTGCTGTTGTAGCTATGTCAAAGAACTGCCCAGACCTTGTGGTCTTCCGTCTCTGCATAATGGGGCGTCACCAGCCTGATCATGTCACTGGAGAACCTATGGATGAAGGATTTGGAGCCATTGTCAAGAATTGCAAGAAGCTCACTCGACTCGCGGTATCTGGTTTATTGACTGATAGAGCTTTTGCTTATATTGGAAAATATGGGAAAATTGTAAGGACCCTATCGGTTGCTTTTGCTGGAGATAGTGACATGGGGCTGAAATATGTGCTTGAGGGTTGTCCCAGATTGCAGAAGCTTGAGATTAGGGACAGTCCATTCGGGGATGCAGCTCTACTTTCTGGTCTGCACCACTATTACAATATGAGATTCCTTTGGATGTCTGCTTGCAAGTTGTCTCGCCAAGGCTGCCAACAGATTGCTCAAGCGTTGCCTCGGCTGGTGGTGGAAGTGATTAAGCATGAAGATAATGTGGACGTGGATGAGTATGTTGATACGTTGTATATGTACCGGTCTCTTGAAGGGCCAAGAGATGATGCGCCTATATTTGTTTCCATCTTGTAG

>PtrFBL8 (Potri.002G102700)

ATGATCACCAACAAAAAGCCTAGATCATCAGACACTGACTCTAATTATATGAGAGACGATCGAACTGACATGTCAGAAGACGACGACCGATCTCCGCCGTCGGATTCAATCGCCAACGACTCTTGCCCAACACGGACCTGCACTCCTGGGTCTGGGTCGGGTTCGTCTTCCATCCCCGAATACTCAGCTCCATACCCGGACCAAGTCCTCGAAAACGTCTTAGAAAACGTTCTCTGGTTCTTAACCTCACGTAAGGACCGAAACGCTGCGTCATTGGTTTGTAGGTTATGGTACCGGGTCGAGGCTATGACCCGATCCGATTTGTTTATCGGTAACTGCTACGCGGTGTCTCCAGAACGCGCTACGTCGCGGTTTACCCGAATCCGTTCGGTGACGCTAAAAGGAAAGCCAAGGTTTGCTGATTTTAACCTGATGCCGCCTAATTGGGGAGCCCACTTCGCGCCTTGGGTCTCTGCTATGGCAAAGGCTTACCCTTGGTTAGAGAAGATTCATTTGAAGAGGATGTCAGTGACGGATGATGATCTGGCTTTGCTTGCGGAGTCATTTTCGGGGTTCAAAGAGCTCGCACTTGTTTGCTGTGATGGGTTTGGTACTAGTGGACTGGCTGTTGTGGCTAGTAAGTGCAGGCAACTCAAAGTGCTTGATCTGATTGAATCAGAAGTATCGGATGATGAAGTGGATTGGATTTTGTGTTTTCCAGATACCGAAACATGTCTTGAATCCCTGATTTTAGATTGTGTAGAATGTCCCATTGATTTTGATGCACTGGAGAGGCTGGTGACTAGGTCCCCATCACTTAAGAAACTTAGGCTAAACAGGTTCGTTTCAATTGGGCAACTATACCGTTTAATGGTTCGAGCACCGCAGCTCACACATCTTGGGACAGGCTCATTTAGCCAATCGGAGGATGTGGCTCAAGGTGAACTGGAACTAGATTATGGCTCTGCGTTTGCTGCTTGCAAATCATTAGTTTGCCTATCTGGATTCAGGGAAATCATTCCAGATTATTTGCCTGCAATATACCCCGTCTGCGCCAATCTCACTTCACTGAACTTTAGTTATGCAAATATCAGCGCAGAACAGCTCAAACCAATTATAAGCAATTGCCACAAGCTTCAGACTTTCTGGGTTCTTGATTCAATATGCGATGAAGGACTTCAGGCTGTGGCTACAACTTGCAAGGAACTACGTGAGCTTCGGGTTTTCCCTTTTGAAGCTAGGGAGGACATCGAGGGCCCTGTTTCTGAAGTGGGCCTCCAAGCGATTTCAGAGGGTTGCAGGAAGCTCCAATCTATTTTGTATTTTTGCCCGCGGATGACAAATGCTGCTGTTATAGCTATGTCAAAGAACTGCCCAGACCTTGTGGCCTTCCGTCTCTGCATAATGGGACTCCACCAGCCTGATCATGTCACTGGAGAACCTATGGATGAAGGGTTTGGAGCCATTGTCATGAATTGCAAGAAGCTCACTCGACTTGCAGTATCTGGTTTATTGACTGATAGAGCTTTTGCTTACATTGGAAAATATGGGAAGATTGTAAGGACATTATCGGTTGCTTTTGCTGGTGATAGTGACATGGGGTTGAAGTATGTGCTTGAGGGCTGTCCCAAATTACAGAAGCTTGAGATTAGAGACAGTCCATTCGGGGATGCAGCTCTACTTTCTGGTCTGCACCACTATTACAATATGAGATTCCTTTGGATGTCCGCTTGCAAGTTGTCCCATCAGGGATGCCAACAGATTGCTCAAGCATTGCCTCACCTGGTGGTGGAAGTGATTAAGCATGAAGATAATGTGGACATGGATGAGTATGTTGATACATTGTACATGTATCGGTCTCTTGCAGGGAGAAGACATGATGTGCCACGATTTGTTTCCATCTTGTAA
