# Supplementary material for: Role of TIR1/AFB family genes during grafting in Carya cathayensis
Source: Front Plant Sci. 2024 Nov 22;15:1494579. doi: 10.3389/fpls.2024.1494579 (PMC11622252; doi:10.3389/fpls.2024.1494579)
Supplement: Supplementary file 3 [file Table3.docx]

**Protein sequences of TIR1-AFB in related species**

>CcTIR1 CCA0981S0065

MPRMGYSFPEEVLEHVFSFIQSDEDRNAISEVCRSWHDIERWSRRRVFVGNCYAISPTMV

IRRFPEVRSVKLKGKPHFADFNLVPEDWGGYVAPWISAMASAYPWLEEIRLKRMVIMDES

LELISKSFKNFKVLVLSSCEGFSTDGLAAIAANCRNLRELDLGESEVEDLSGHWLSRFPD

NYTSMVSLNIACLGWEVSFSALERLVGRCPNLRTLRLNRAVPLDKLANLLRRAPQLVELG

TGAYSSELRPDVFSNLSGAFSGCKELKSLSGFWDVVPGYLPAVYPICSKLKSLNLSYANV

QSPELIKLVSQCQNLQRLWVLDYIEDAGLDALAASCKDLRELRVFPSDPFGPEPNVALTE

QGLVSVSEGCPKLQSVLYFCRQMSNAALMTIARNRPNFTRFRLCIIEPHTPDYLTLQPLD

VGFGAIVEHCKDLRRLSLSGLLTDRVFEYIGTHAKKLEMLSVAFAGESDLGLHHILSGCD

NLRKLEIRDCPFGDKALLANAAKLETMRSLWMSSCSVSFGACKLLGQKMPGLNVEVIDER

GPPDSRPESCPIEKLYVYRTVSGRRFDMPGFVWTMAENPALRLS

>CcAFB1 CCA0535S0053

MQRMACSFPDEVLEHVFSFVQCDNDRNAISTVCKSWYKIERWSRRRVFVGNCYAISPGMV

IRRFPDVRSIELKGKPHFADFNLVPDGWGGYVAPWISAMAAAYTWLEEIRLKRMVITDEN

LELISKSFKNFKVLVLSSCEGFSTDGLAAIAANCRNLRELDLRESEVDDRSGHWLKHFPD

DYTSLVSLNIACLGCEVSFSALERLVGRCPNLRTLRLNRAVPLDKLANLLRRAPQLVELG

TGAYSSELRPDVFSNLSGAFSGCKELKSLSGFWEVVPGYLPAVYPICCRLTTLNLSYATI

QSPDLIKLVGQCQNLQRLWVLDYIEDAGLDAVAASCKDLRELRVFPSDPFGPEPNVPLTE

QGLVSISEGCSKLQSVLYFCRQMSNAALMTIARNRPNMTRFRLCIIEPGTPDYLTLQPLD

VGFGAIVEHCKDLQRLSLAGLLTDRVFEYIGTYAKKLEMLSVAFAGESDLGLHHVLSGCE

NLRKLEIRDCPFGDKALLANAAKLETMRSLWMSSCSVTFGACKLLGQKMPRLNVEVIDES

GPPDSRPESCPIEKLYIYRTVAGSRFDMPGFVWTIDEDSAMRLS

>CcAFB2 CCA1250S0070

MNYFPDEVLEHVFDFLTSHRDRNAVSLVCSLWYRVERFSRQRVFIGNCYAISPEKLITRF

PGLKSLTLKGKPHFADFNLVPYDWGGYVEPWIEALAKSRIGLEELRLKRMMVSDESLELL

SRSFANFKTLVLVSCEGFTTNGLAAIAANCRFLRELDLQENEIDDHSGHWLSCFPDSCTS

LTSLNFACLKGEINSASLERLVARSPNLRSLRLNRSVPLETLQKILVRAPLLVDLGTGSY

VHDPDSETYNKLKTTFLNCKSIRNLSGFLEVAPRCLPAIFPICLNLTYLNLSYAAGIPGS

ELIKLIGRCAKLQRLWILDCIGDKGLEVVASTCNELQELRVFPSDPLGIGHDAVTENGLV

AISIGCPKLHSLLYFCQQMTNAALITVARNCPNFIRFRLCILDPTKPDAVTMQPLDDGFG

AIVQSCKLLRRLSLTGLLTDQVFLYIGMYAEQLEMLSVAFAGNSDKGMLYVLNGCKKLRK

LEIMDCPFGNRALLSDVGKYETMRSLWMSSCEVTLWGCKTLAKMMPRLNVEIINEHDQME

ICPEDDGQRVEKMYLYRTLVGPRKDAPEFVWNL

>CcAFB3 CCA1539S0035

MNYFPDEVLEHVFDFVASHRDRNAVSLVCRLWHRVERLSRQRVFVGNCYALNPEILIARF

PGLKSLTLKGKPHFADFNLVPHDWGGYVQPWIEALSNSRIGLEELRLKRMVVSDESLELL

SRSFANFKSLVLVSCEGFTTVGLAAIAANCRFLRELDLQENDIDDHSGHWLSCFPDSCTS

LISLNFACLKGEINLAALERLVARSPNLRSLRLNRAVPLETLQNILMRVPQLVDLGTGSY

IHDPDSETYNKLKHTILKCKSIRNLSGFLEVAPRCLPAIYPICLNLTSLNLSYAAGIHGS

ELVKLIRHCAKLQRLWILDCIGDKGLEVVASTCKELQELRVFPSDLLGVGHAAVTENGLV

AISVGCPKLHSLLYFCQQMTNAALIVVAKNCPNFIRFRLCILEPTKPDPVTMQPLDDGFG

AIVQSCKRLRRLSLSGLLTDRVFLYIGMYAEQLEMLSVAFAGDSDKGMLYVLNGCKKLRK

LEIRDCPFGDKALLTDVGKYETMRSLWMSSCEVTLGGCKTLAKGMPRLNVEIINEHDQME

IGSEEQRVEKMYLYRTLVGPRKDAPEFVWTL

>CcAFB4 CCA0533S0235

MRKDRTEMSEDDDRSPPLDLRGADIAESSNKTRNCTGVSGSGGPVPGPGPSSMEFQAPYP

DQVLENVLENVLQFLSSRRDRNAASLVCRSWWRVEALTRSDLFIGNCYSVSPRRATARFT

RVRSVSIKGRPRFADFNLMPPDWGAHFAPWVTAMASAYPWLEKVYLKRMSVTDDDLALLA

ESFPSFKELVLVCCDGFGTSGLAVIASKCRQLKVLDLIESEVTDDEVDWIACFPESGATC

LESLIFDCVECPINFDALERLVARSPSLKKLRLNRFVSIGQLYLLMVRSPQLTHLGTGSF

SAPEGMAQGEQEPDFFTAFAACRSLVCLSGFREILPDYLPAIHPVCANLTSLNFSYANIN

AEQLKLVIFHCHKLQIFWVLDSICDEGLQAVASTCKDLRELRVFPFDAREDNEGPVSDLG

LLAISEGCRKLQSILYFCQHMTNAAVIAMSRNCPDLEVFRLCIMGRHRPDRVTNEPMDEG

FGAIVMNCKKLTRLAVSGLLTDRAFNYIGKYGKLVRTLSVAFAGDSDMGLKYVLEGCPRL

QKLEIRDSPFGDAALRSGLHHYYNMRFLWMSSCRLFPQGCQEIARALPRLVVEVIRDDAI

EHTDETVDILYMYRSLEGPRDDAPGFVTIMQ

>CcAFB5 CCA1097S0042

MRKDRTGISQDDDRYPPADLRGAESSNKTRNCCGVGSVSGSTGPISMEFQAPCPDQVLEN

VLENVLQFLSSRRDRNAASLVCKSWWRVEALTRSDLYIGNCYSVSPRRAMNRFSRVQSVS

IKGRPRFADFNLMPIDWGAHFAPWVTAMASAYPWLEKVYLKRMSVTDDDLALLADSFPSF

KELVIVCCDGFSTSGLALVASDCRQLKVLDLIESEVTDDDVDWIACFPESGATCLESLIF

DCVECSINFDALESLVARSPSLKKLRLNCNVSIGQLCRLIVRAPQLTHLGTGSYSSPDDA

VPGEHASDLQSALSACKSLVCLSGFKEMLPDYLPAIYPVCANLTSLNFSYANIHADQIRS

VIFHCHNLQVFWVLDSIRDEGLQAVASTCKDLRELRVFPLDARGDNEGPVSDVGLLAISE

GCRKLRSILYFCQHMTNAAVIAMSRNCPDLEVFRLCIIALHSPDHVTGKPMDEGFGAIVM

NCKKLTRLAVSGLLTDQAFGYIGKYGKLLRTLSVAFAGDSDMGLKYVLEGCPRLQKLEIR

DSPFGDAALHSGLHHYYNMRFLWMSSCRLSPKGCQEIVRALPRLVVEVIKHDVAVCPDDT

VDILYMYRSLEGPRDDAPKVVTILQ

>CcAFB6 CCA0729S0024

MDPERKKVLERVAISTFPDEVLERVLVQLKSHKDRSSVSLVCHSWYDAERFSRTHVFIAN

CYSVTPEIVSRRFPNIRSVSLKGKPRFSDFNLVPPKWGADIRSWLVMFAAKYPFLEELRL

KRMTVTDESLEFLALSFPNFKGLSLLSCDGFSTGGLAAIATHCKNLTELDIQENDLNDKS

GSWLSCFPENFSSLEVLNFANLSNDVNFEALERLVSRCKSLKVLKVNRNITLEQLPRLLI

HAPLLTELGTGSFLQEHPACHYSELENAFNKCMNLNTLSGLSEVTALSLPALYPACANLT

FLNLSCAALQSDELAKLLVHCPYLRRLWVLDTVEDKGLEAVGSNCPLLEELRVFPTDPFD

EDTFHGVTESGFIAVSYGCRRLSYVLYFCRQMTNAAVKTIVQNCPDFTHFRLCIMNPWQP

DYQTNEPMDEAFCAVVKTCTKLQRLAVSGLLTDLTFEYIGKYAKNLETLSVAFAGSSDWG

MQCVLGGCPKLRKLEIRDCPFGNAALLSGLDKYESMRSLWMSDCKVTMNGCRLLAKEMPR

LNVEVIKVDGSNESQADKVYVYRSVAGPRRDAPPFVLTL

>AtTIR1

MQKRIALSFPEEVLEHVFSFIQLDKDRNSVSLVCKSWYEIERWCRRKVFIGNCYAVSPAT

VIRRFPKVRSVELKGKPHFADFNLVPDGWGGYVYPWIEAMSSSYTWLEEIRLKRMVVTDD

CLELIAKSFKNFKVLVLSSCEGFSTDGLAAIAATCRNLKELDLRESDVDDVSGHWLSHFP

DTYTSLVSLNISCLASEVSFSALERLVTRCPNLKSLKLNRAVPLEKLATLLQRAPQLEEL

GTGGYTAEVRPDVYSGLSVALSGCKELRCLSGFWDAVPAYLPAVYSVCSRLTTLNLSYAT

VQSYDLVKLLCQCPKLQRLWVLDYIEDAGLEVLASTCKDLRELRVFPSEPFVMEPNVALT

EQGLVSVSMGCPKLESVLYFCRQMTNAALITIARNRPNMTRFRLCIIEPKAPDYLTLEPL

DIGFGAIVEHCKDLRRLSLSGLLTDKVFEYIGTYAKKMEMLSVAFAGDSDLGMHHVLSGC

DSLRKLEIRDCPFGDKALLANASKLETMRSLWMSSCSVSFGACKLLGQKMPKLNVEVIDE

RGAPDSRPESCPVERVFIYRTVAGPRFDMPGFVWNMDQDSTMRFSRQIITTNGL

>AtAFB3

MNYFPDEVIEHVFDFVASHKDRNSISLVCKSWHKIERFSRKEVFIGNCYAINPERLIRRF

PCLKSLTLKGKPHFADFNLVPHEWGGFVHPWIEALARSRVGLEELRLKRMVVTDESLDLL

SRSFANFKSLVLVSCEGFTTDGLASIAANCRHLRELDLQENEIDDHRGQWLNCFPDSCTT

LMSLNFACLKGETNVAALERLVARSPNLKSLKLNRAVPLDALARLMSCAPQLVDLGVGSY

ENEPDPESFAKLMTAIKKYTSLRSLSGFLEVAPLCLPAFYPICQNLISLNLSYAAEIQGN

HLIKLIQLCKRLQRLWILDSIGDKGLAVVAATCKELQELRVFPSDVHGEEDNNASVTEVG

LVAISAGCPKLHSILYFCKQMTNAALIAVAKNCPNFIRFRLCILEPHKPDHITFQSLDEG

FGAIVQACKGLRRLSVSGLLTDQVFLYIGMYAEQLEMLSIAFAGDTDKGMLYVLNGCKKM

RKLEIRDSPFGNAALLADVGRYETMRSLWMSSCEVTLGGCKRLAQNSPRLNVEIINENEN

NGMEQNEEDEREKVDKLYLYRTVVGTRKDAPPYVRIL

>AtAFB2

MNYFPDEVIEHVFDFVTSHKDRNAISLVCKSWYKIERYSRQKVFIGNCYAINPERLLRRF

PCLKSLTLKGKPHFADFNLVPHEWGGFVLPWIEALARSRVGLEELRLKRMVVTDESLELL

SRSFVNFKSLVLVSCEGFTTDGLASIAANCRHLRDLDLQENEIDDHRGQWLSCFPDTCTT

LVTLNFACLEGETNLVALERLVARSPNLKSLKLNRAVPLDALARLMACAPQIVDLGVGSY

ENDPDSESYLKLMAVIKKCTSLRSLSGFLEAAPHCLSAFHPICHNLTSLNLSYAAEIHGS

HLIKLIQHCKKLQRLWILDSIGDKGLEVVASTCKELQELRVFPSDLLGGGNTAVTEEGLV

AISAGCPKLHSILYFCQQMTNAALVTVAKNCPNFIRFRLCILEPNKPDHVTSQPLDEGFG

AIVKACKSLRRLSLSGLLTDQVFLYIGMYANQLEMLSIAFAGDTDKGMLYVLNGCKKMKK

LEIRDSPFGDTALLADVSKYETMRSLWMSSCEVTLSGCKRLAEKAPWLNVEIINENDNNR

MEENGHEGRQKVDKLYLYRTVVGTRMDAPPFVWIL

>AtAFB1

MGLRFPPKVLEHILSFIDSNEDRNSVSLVCKSWFETERKTRKRVFVGNCYAVSPAAVTRR

FPEMRSLTLKGKPHFADYNLVPDGWGGYAWPWIEAMAAKSSSLEEIRMKRMVVTDECLEK

IAASFKDFKVLVLTSCEGFSTDGIAAIAATCRNLRVLELRECIVEDLGGDWLSYFPESST

SLVSLDFSCLDSEVKISDLERLVSRSPNLKSLKLNPAVTLDGLVSLLRCAPQLTELGTGS

FAAQLKPEAFSKLSEAFSNCKQLQSLSGLWDVLPEYLPALYSVCPGLTSLNLSYATVRMP

DLVELLRRCSKLQKLWVMDLIEDKGLEAVASYCKELRELRVFPSEPDLDATNIPLTEQGL

VFVSKGCRKLESVLYFCVQFTNAALFTIARKRPNLKCFRLCVIEPFAPDYKTNEPLDKGF

KAIAEGCRDLRRLSVSGLLSDKAFKYIGKHAKKVRMLSIAFAGDSDLMLHHLLSGCESLK

KLEIRDCPFGDTALLEHAAKLETMRSLWMSSCFVSFGACKLLSQKMPRLNVEVIDEHPPE

SRPESSPVERIYIYRTVAGPRMDTPEFVWTIHKNPENGVSHLAIK

>AtAFB4

MTEEDSSAKMSEDVEKYLNLNPPCSSSSSSSSAATFTNKSRNFKSSPPPCPDHVLENVLE

NVLQFLTSRCDRNAVSLVCRSWYRVEAQTRLEVFIGNCYSLSPARLIHRFKRVRSLVLKG

KPRFADFNLMPPNWGAQFSPWVAATAKAYPWLEKVHLKRMFVTDDDLALLAESFPGFKEL

TLVCCEGFGTSGIAIVANKCRQLKVLDLMESEVTDDELDWISCFPEGETHLESLSFDCVE

SPINFKALEELVVRSPFLKKLRTNRFVSLEELHRLMVRAPQLTSLGTGSFSPDNVPQGEQ

QPDYAAAFRACKSIVCLSGFREFRPEYLLAISSVCANLTSLNFSYANISPHMLKPIISNC

HNIRVFWALDSIRDEGLQAVAATCKELRELRIFPFDPREDSEGPVSGVGLQAISEGCRKL

ESILYFCQNMTNGAVTAMSENCPQLTVFRLCIMGRHRPDHVTGKPMDDGFGAIVKNCKKL

TRLAVSGLLTDEAFSYIGEYGKLIRTLSVAFAGNSDKALRYVLEGCPKLQKLEIRDSPFG

DVGLRSGMHRYSNMRFVWLSSCLISRGGCRGVSHALPNVVVEVFGADGDDDEDTVTGDYV

ETLYLYRSLDGPRKDAPKFVTIL

>AtAFB5

MTQDRSEMSEDDDDQQSPPLDLPSTAIADPCSSSSSPNKSRNCISNSQTFPDHVLENVLE

NVLQFLDSRCDRNAASLVCKSWWRVEALTRSEVFIGNCYALSPARLTQRFKRVRSLVLKG

KPRFADFNLMPPDWGANFAPWVSTMAQAYPCLEKVDLKRMFVTDDDLALLADSFPGFKEL

ILVCCEGFGTSGISIVANKCRKLKVLDLIESEVTDDEVDWISCFPEDVTCLESLAFDCVE

APINFKALEGLVARSPFLKKLRLNRFVSLVELHRLLLGAPQLTSLGTGSFSHDEEPQSEQ

EPDYAAAFRACKSVVCLSGFRELMPEYLPAIFPVCANLTSLNFSYANISPDMFKPIILNC

HKLQVFWALDSICDEGLQAVAATCKELRELRIFPFDPREDSEGPVSELGLQAISEGCRKL

ESILYFCQRMTNAAVIAMSENCPELTVFRLCIMGRHRPDHVTGKPMDEGFGAIVKNCKKL

TRLAVSGLLTDQAFRYMGEYGKLVRTLSVAFAGDSDMALRHVLEGCPRLQKLEIRDSPFG

DVALRSGMHRYYNMRFVWMSACSLSKGCCKDIARAMPNLVVEVIGSDDDDDNRDYVETLY

MYRSLDGPRNDAPKFVTIL

>OsTIR1 (LOC_Os05g05800).dna (1785 bp)

MGRGGSRAACAAAAPPWHSLPDEVWEHAFSFLPAAADRGAAAGACSSWLRAERRSRRRLA

VANCYAAAPRDAVERFPSVRAAEVKGKPHFADFGLVPPAWGAAAAPWIAAAADGWPLLEE

LSFKRMVVTDECLEMIAASFRNFQVLRLVSCDGFSTAGLAAIAAGCRHLRELDLQENEIE

DCSIHWLSLFPESFTSLVTLNFSCLEGEVNITVLERLVTRCHNLKTLKLNNAIPLDKLAS

LLHKAPQLVELGTGKFSADYHSDLFAKLEAAFGGCKSLRRLSGAWDAVPDYLPAFYCVCE

GLTSLNLSYATVRGPELIKFISRCRNLQQLWVMDLIEDHGLAVVASSCNKLQELRVFPSD

PFGAGFLTERGLVDVSASCPMLESVLYFCRRMTNEALITIAKNRPNFTCFRLCILEPHTP

DYITREPLDAGFSAIVESCRGLRRLSISGLLTDLVFKSIGAHADRLEMLSIAFAGNSDLG

LHYILSGCKSLKKLEIRDCPFGDKPLLANAAKLETMRSLWMSSCLLTLGACRQLARKMPR

LSVEIMNDPGRSCPLDSLPDETPVEKLYVYRTIAGPRCRMKIHSLFQDMEMHGL

>OsAFB2 (LOC_Os04g32460).dna (1728 bp)

MTYFPEEVVEHIFSFLPAQRDRNTVSLVCKVWYEIERLSRRGVFVGNCYAVRAGRVAARF

PNVRALTVKGKPHFADFNLVPPDWGGYAGPWIEAAARGCHGLEELRMKRMVVSDESLELL

ARSFPRFRALVLISCEGFSTDGLAAVASHCKLLRELDLQENEVEDRGPRWLSCFPDSCTS

LVSLNFACIKGEVNAGSLERLVSRSPNLRSLRLNRSVSVDTLAKILLRTPNLEDLGTGNL

TDDFQTESYFKLTSALEKCKMLRSLSGFWDASPVCLSFIYPLCAQLTGLNLSYAPTLDAS

DLTKMISRCVKLQRLWVLDCISDKGLQVVASSCKDLQELRVFPSDFYVAGYSAVTEEGLV

AVSLGCPKLNSLLYFCHQMTNAALVTVAKNCPNFTRFRLCILEPGKPDVVTSQPLDEGFG

AIVRECKGLQRLSISGLLTDKVFMYIGKYAKQLEMLSIAFAGDSDKGMMHVMNGCKNLRK

LEIRDSPFGDAALLGNFARYETMRSLWMSSCNVTLKGCQVLASKMPMLNVEVINERDGSN

EMEENHGDLPKVEKLYVYRTTAGARDDAPNFVKIL

>OsAFB3 (LOC_Os11g31620).dna (1707 bp)

MVFFPEEVVEHILGFLASHRDRNAVSLVCREWYRVERLSRRSVLVRNCYAARPERVHARF

PGLRSLSVKGRPRFVPAGWGAAARPWVAACVAACPGLEELRLKRMVVTDGCLKLLACSFP

NLKSLVLVGCQGFSTDGLATVATNCRFMKELDLQESLVEDRDSRWLGCFPKPSTLLESLN

FSCLTGEVNSPALEILVARSPNLRSLRLNRSVPLDVLARILCRRPRLVDLCTGSFVRGNI

VGAYAGLFNSFQHCSLLKSLSGFWDATSLFIPVIAPVCKNLTCLNLSSAPMVRSAYLIEF

ICQCKKLQQLWVLDHIGDEGLKIVASSCIQLQELRVFPANANARASTVTEEGLVAISAGC

NKLQSVLYFCQRMTNSALITVAKNCPRFTSFRLCVLDPGSADAVTGQPLDEGYGAIVQSC

KGLRRLCLSGLLTDTVFLYIGMYAERLEMLSVAFAGDTDDGMTYVLNGCKNLKKLEIRDS

PFGDSALLAGMHQYEAMRSLWLSSCNVTLGGCKSLAASMANLNIEVMNRAASINEADNAN

DAKKVKKLYIYRTVAGPRGDAPEFISTF

>OsAFB4 (LOC_Os02g52230).dna (2004 bp)

MSTSPSCSSSSPIPQSLTLASTSSSSSSSGMRDAGEGSDSPPSEMSEDGSGGSGDGDGDG

DGGGGGGDRWMPDLRGGNGGGGGGGGGGGRWAPPDQVLENVLESVLEFLTAARDRNAASL

VCRSWYRAEAQTRRELFIGNCYAVSPRRAVERFGGVRAVVLKGKPRFADFSLVPYGWGAY

VSPWVAALGPAYPHLERICLKRMTVSNDDLALIAKSFPLFKELSLVCCDGFSTLGLAAIA

ERCRHLRVLDLIEDYIDEEEDELVDWISKFPESNTSLESLVFDCVSVPFNFEALEALVAR

SPAMRRLRMNHHVTVEQLRRLMARAPQLTHLGTGAFRSEPGPGGALSVTELATSFAASRS

LICLSGFRDVNPEYLPAIHPVCANLTSLNFSFANLTAEELTPIIRNCVRLRTFWVLDTVG

DEGLRAVAETCSDLRELRVFPFDATEDSEGSVSDVGLQAISEGCRKLESILYFCQRMTNA

AVIAMSKNCSDLVTFRLCIMGRHRPDRITGEPMDDGFGAIVMNCKKLTRLSVSGLLTDKA

FAYIGKYGKLIKTLSVAFAGNSDMSLQSVFEGCTRLQKLEVRDSPFSDKGLLSGLSYFYN

MRFLWMNSCRLTMRGCRDVAQQMPDLVVEVMKDHLDDEGEMETVDKLYLYRSLAGARNDA

PSFVNIL

>OsAFB5 (LOC_Os03g08850).dna (1812 bp)

MSEEDDDQPPPLPAQKRPRASPPPDQVLDNVLETVLQFLDSARDRCAASLVCRSWSRAES

ATRASVAVRNLLAASPARVARRFPAARRVLLKGRPRFADFNLLPPGWAGADFRPWAAAVA

AAAFPALASLFLKRITVTDDDLDLVSRSLPASFRDLSLLLCDGFSSAGLASIASHCRGLR

VLDVVDCEMNDDDDEVVDWVAAFPPGTTDLESLSFECYVRPVSFAALEALVARSPRLTRL

GVNEHVSLGQLRRLMANTPRLTHLGTGAFRPGDGPEDVGLDIEQMASAFASAGRTNTLVS

LSGFREFEPEYLPTIAAVSGNLTNLDFSYCPVTPDQFLPFIGQCHNLERLYVLDSVRDEG

LQATARTCKKLQVLHVLPLNALEDADELVSEVGLTAIAEGCRGLRSTLYFCQSMTNAAVI

AISQNCVDLKVFRLCIMGRHQPDHVTGEPMDEGFGAIVRNCSKLTRLSTSGHLTDRAFEY

IGKYAKSLRTLSVAFAGDSNLALQHILQGCSKLEKLEIRDCPFGDAGLLSGMHHFYNMRF

LWMSGCNLTLQGCKEVARRLPRLVVELINSQPENERTDSVDILYMYRSLEGPREDVPPFV

KIL

>PtrFBL1 (Potri.014G134800)

MLRKANSFPEEVLQHVLSFITNDKDRNAVSLVCKSWYEIERWCRKRIFVGNCYAVRPEMV

IRRFPELRSVELKGKPHFADFSLVPDGWGGCVYPWIAALATAYPWLEEISLERMVVSDES

LKVIAKSFKNFKVLVLSSCEGFSTDGLAAVAANCRNLRGLDLRESEVDDPSGQWLSRFPD

SFTSLASLNISCLGAEVSFSALERLVGRCPDLKTLRLNHAVPLDKLANLLRGAPQLVELG

TGAYSAELQPDVFSNLAGAFSGCKELRSLSGFWNVFPGYLPAVYPVCSGLTSLNLRYANI

QGADLIKLVSQCPSLQRLWVLDYIEDIGLEALAACCKDLTELRVFPSDPYGAEPNVSLTE

RGLVSVSEGCPKLHSVLYFCRQMTNAALVTIAKNRPSMTCFRLCIIEPRAPDYQTLQPLD

LGFGAIVENYKDLRRLSLSGLLTDRVFEYIGTYAKKLEMLSVAFAGDSDLGLHHVLSGCE

KLCKLEIRDCPFGDKALLANAAKLETMRSLWMSSCSVSFRACKLLGQKMPRLNVEVIDER

GPPDLRPESCPVEKLYIYRTIAGPRFDMPGFVWTMDEDSVSRFS

>PtrFBL2 (Potri.002G207800)

MPNKASTFPEEVLEHVLSFITNDKDRNAVSVVCKSWYEIERWCRKRIFVGNCYAVRPDMV

IRRFPELRSVELKGKPHFADFNLVPDGWGGYFYPWIAALATAYPWLEEIRLKRMVISDES

LEFIAKSFKNFKVLVLSSCEGFSTDGLSAIAADCRNLRELDLRESEVDDPSGQWLNSFPD

SFTSLVSLNISCLGAELSFSALERLVGQCPDLKNLQLNHAVPVERLAKLIRQAPQLVELG

TGEFSAKLQPEIFSNLAGAFSVCKELRSLSGFWDVNPAYLPAVYPVCSGLTSLNLRYANI

QSADLIKLVSQCSNLQRLWVLDYIEDVGLEALAACCKDLTELRVFPSDPFAAEPNVSLTE

RGLVSVSEGCPKLQSVLYFCRQMTNAALVTVAKNRPSMTCFRLCIIEPQAPDYQTLQPLD

LGFGAIVENYKDLRRLSLSGLLTDRVFEYIGTYGKKIEMLSVAFAGDSDLGLHHVLSGCE

RLCKLEIRDCSFGDKALLANAAKLETMRSLWMSSCSVSFGACKLLGQKMPRLNVEVIDER

GPPESRPESCPVEKLYIYRTIAGPRLDMPGFVRTMDADSVSRFC

>PtrFBL3 (Potri.001G323100)

MNYFPDEVLEHIFDFVTSQRDRNSVSQVCKPWYKIESTSRQKVFVGNCYAISPERVIERF

PGLKSITLKGKPHFADFNLVPHDWGGFVYPWIEAFARNNMGLEELKLKRMIISDECLELI

SRSFANFKSLVLVSCEGFSTDGLAAIASNCRFLRELDLQENDVEDHRGHWLSFFPDTCTS

LVSLNFACLKGDVNLAALERLVARSPNLRSLRLNHAVPLDILQKILMRAPHLVDLGVGSY

VHDPDSETYNKLVTALQKCKSVKSLSGFLEAAPQCLSAFHLICPNLTSLNLSYAPGIHGT

ELIKLIRHCRKLQRLWILDCIGDEGLEVVASTCKHLQEIRVFPSDPFVGNAAVTEVGLVA

LSSGCRNLHSILYFCQQMTNAALITVAKNCPNFTRFRLCILDPTKPDADTNQPLDEGFGA

IVHSCKGLRRLSMSGLLTDQVFLYIGMYAEQLEMLSIAFAGDTDKGMQYLLNGCKKLRKL

EIRDCPFGNAALLMDVGKYETMRSLWMSSCDITLGGCKSLAKKMPRLNVEIINESDQMDI

TADDGQKVEKMFLYRTLAGRRKDAPEFVWTL

>PtrFBL4 (Potri.017G061600)

MNYFPDEVLEHIFDFVTSQRDRNSVSQVCKPWYKIESSSRQKVFVGNCYAISPQRVIERF

PGLKSITLKGKPHFADFNLVPNDWGGFVYPWIEAFARNSVGLEELKLKRMIISDECLELI

SRSFPNFKSLVLVSCEGFTADGLAAIASNCRFLRELDLQENDVEDHRGHWLSCFPDTCTS

LVSLNFACLKGEVNVAALERLIARSPNLRSLRLNHAVPLDVLQKILIRAPHLVDLGVGSY

VNDPDSETYNKLVMAIQKCMSVKSLSGFLEVAPHCLSAFHLICPNLTSLNLSYAPGIHGA

ELIKLIRHCMKLQRLWILDCIGDQGLEVVASTCKDLQEIRVFPSDPHVGNAAVTEVGLVA

LSSGCRKLHSILYFCQQMTNVALITVAKNCPNFTRFRLCILDPTKPDAVTNQPLDEGFGA

IVHSCKGLRRLSMTGLLTDKVFLYIGMYAEQLEMLSIAFAGDTDKGMQYLLNGCKKLRKL

EIRDCPFGNAALLMDVGKYETMRSLWMSSCEVTLGGCKSLAKKMPRLNVEIINENDQMDA

SADDRQKVEKMFLYRTLAGRREDAPEFVWTL

>PtrFBL5 (Potri.004G033900)

MGPNPKMKREFLDSTRSSPFPDEVLERVLSLLKSHKDRSAVSLVCKDWYNAESWSRTHVF

IGNCYSVSPEIVARRFPIIKSVTLKGKPRFSDFNLVPENWGADVHPWLVVFATKYPFLEE

LRLKRMAVSDESLEFLAVNFPNFKVLSLLSCDGFSTDGLAAIATHCKSLTQLDIQENGID

DKSGGWLSCFPENFTSLEVLNFANLNTDVNFDALERLVSRCKSLKVLKVNKSISLEHLQR

LLVCAPQLTELGTGSFTPELTTRQYAELESAFNQCKNLHTLSGLWEATALYLPVLYPVCS

NLTFLNLSYTFLQSLELASLLRQCPRLRRLWVLDTVGDKGLEAVGSNCPLLEELRVFPAD

PFDEEIIHGVTEAGFVAVSYGCRRLHYVLYFCRQMTNAAVATIVQNCPDFTHFRLCIMNP

GQPDYLTNEPMDEAFGAVVRTCTKLQRLSVSGLLTDLTFEYIGQYAKNLETLSVAFAGSS

DRGMQCVLEGCPKLRKLEIRDCPFGNAALLSGLEKYESMRSLWMSACNVTMNGCRLLARE

MPRLNVEVMKEDGSDDSQADKVYVYRSVAGPRRDAPPCVLTLSGL

>PtrFBL6 (Potri.011G042400)

MDSNPKMRKEFLDSTRSSLFPDEVLERVLSLLKSHKDRSAVSLVCKDWYNAESWSRTHVF

IGNCYSVSPEIVARRFPRIKSVTLKGKPRFSDFNLVPENWGADVHPWFVVFAAKYPFLEE

LRLKRMAVSDESLEFLALNFPNFKVLSLLSCDGFSTDGLAAIATHCKNLTQLDIQENGID

DKSGNWLSCFPENFTSLEVLNFANLNTDVNFDALERLVSRCKSLKVLKANKSISLEQLQR

LLVCAPQLTELGTGSFMPELTARQYAELGSSFNQLKNLNTLSGLWEATAPYLPVLYPACT

NLTFLNLSYAFLQSIELASLLCQCPRLRRLWVLDTVGDKGLEAVGSNCPLLEELRVFPAD

PFDEEVIHGVTEAGFLAVSYGCRRLHYVLYFCRQMTNAAVATIVQNCPDFTHFRLCIMNP

GQPDYLTNEPMDEAFGAVVRTCTKLQRLSVSGLLTDLTFEYIGQYAKNLETLSVAFAGSS

DRGMQCMLEGCPKLRKLEIRDCPFGNAALLSGLEKYESMRSLWMSACNVTMNGCRVLARE

MPRLNVEVMKEDGSDDSQADKVYVYRSVVGPRRDAPPCVLTLSGL

>PtrFBL7 (Potri.005G159300)

MITNKKPRSSDTDSNYMRDDRTEMSEDDDRSPPSNSITHDSSPTRTCTPGPGSGSSSVPE

YLAPYPDQVLENVLENVLWFLTSRKDRNAASLVCRSWYRVEALTRSDLFIGNCYAVSPKR

AMSRFTRIRSVTLKGKPRFADFNLMPPNWGAHFAPWVSAMAMTYPWLEKVHLKRMSVTDD

DLALLAESFSGFKELVLVCCDGFGTSGLAIVVSRCRQLKVLDLIESEVSDDEVDWISCFP

DTETCLESLIFDCVDCPIDFDALERLVARSPSLKKLRLNRYVSIGQLYRLMVRAPHLTHL

GTGSFSPSEDVAQVEQGPDYASAFAACKSLVCLSGFRELIPDYLPAINPVCANLTSLNFS

YAEVSAEQLKPIISNCHKLQIFWVLDSICDEGLQAVAATCKELRELRVFPVDPREDIEGP

VSEVGLQAISEGCRKLQSILYFCHRMTNAAVVAMSKNCPDLVVFRLCIMGRHQPDHVTGE

PMDEGFGAIVKNCKKLTRLAVSGLLTDRAFAYIGKYGKIVRTLSVAFAGDSDMGLKYVLE

GCPRLQKLEIRDSPFGDAALLSGLHHYYNMRFLWMSACKLSRQGCQQIAQALPRLVVEVI

KHEDNVDVDEYVDTLYMYRSLEGPRDDAPIFVSIL

>PtrFBL8 (Potri.002G102700)

MITNKKPRSSDTDSNYMRDDRTDMSEDDDRSPPSDSIANDSCPTRTCTPGSGSGSSSIPE

YSAPYPDQVLENVLENVLWFLTSRKDRNAASLVCRLWYRVEAMTRSDLFIGNCYAVSPER

ATSRFTRIRSVTLKGKPRFADFNLMPPNWGAHFAPWVSAMAKAYPWLEKIHLKRMSVTDD

DLALLAESFSGFKELALVCCDGFGTSGLAVVASKCRQLKVLDLIESEVSDDEVDWILCFP

DTETCLESLILDCVECPIDFDALERLVTRSPSLKKLRLNRFVSIGQLYRLMVRAPQLTHL

GTGSFSQSEDVAQGELELDYGSAFAACKSLVCLSGFREIIPDYLPAIYPVCANLTSLNFS

YANISAEQLKPIISNCHKLQTFWVLDSICDEGLQAVATTCKELRELRVFPFEAREDIEGP

VSEVGLQAISEGCRKLQSILYFCPRMTNAAVIAMSKNCPDLVAFRLCIMGLHQPDHVTGE

PMDEGFGAIVMNCKKLTRLAVSGLLTDRAFAYIGKYGKIVRTLSVAFAGDSDMGLKYVLE

GCPKLQKLEIRDSPFGDAALLSGLHHYYNMRFLWMSACKLSHQGCQQIAQALPHLVVEVI

KHEDNVDMDEYVDTLYMYRSLAGRRHDVPRFVSIL
